# Tailored protein encapsulation into a DNA host using geometrically organized supramolecular interactions

**DOI:** 10.1038/ncomms14472

**Published:** 2017-02-16

**Authors:** Andreas Sprengel, Pascal Lill, Pierre Stegemann, Kenny Bravo-Rodriguez, Elisa-C. Schöneweiß, Melisa Merdanovic, Daniel Gudnason, Mikayel Aznauryan, Lisa Gamrad, Stephan Barcikowski, Elsa Sanchez-Garcia, Victoria Birkedal, Christos Gatsogiannis, Michael Ehrmann, Barbara Saccà

**Affiliations:** 1 Centre for Medical Biotechnology (ZMB), Centre for Nano Integration Duisburg-Essen, University of Duisburg-Essen, Universitätstrasse 2, Essen 45117, Germany; 2 Department of Structural Biochemistry, Max-Planck-Institute of Molecular Physiology, Otto-Hahn-Straße 11, Dortmund 44227, Germany; 3 Theory Department, Max-Planck-Institut für Kohlenforschung, Kaiser-Wilhelm-Platz 1, Mülheim an der Ruhr 45470, Germany; 4 Department of Chemistry and Interdisciplinary Nanoscience Center, Aarhus University, Gustav Wieds Vej 14, Aarhus 8000, Denmark; 5 School of Biosciences, Cardiff University, Cardiff CF10 3US, UK

## Abstract

The self-organizational properties of DNA have been used to realize synthetic hosts for protein encapsulation. However, current strategies of DNA–protein conjugation still limit true emulation of natural host–guest systems, whose formation relies on non-covalent bonds between geometrically matching interfaces. Here we report one of the largest DNA–protein complexes of semisynthetic origin held in place exclusively by spatially defined supramolecular interactions. Our approach is based on the decoration of the inner surface of a DNA origami hollow structure with multiple ligands converging to their corresponding binding sites on the protein surface with programmable symmetry and range-of-action. Our results demonstrate specific host–guest recognition in a 1:1 stoichiometry and selectivity for the guest whose size guarantees sufficient molecular diffusion preserving short intermolecular distances. DNA nanocontainers can be thus rationally designed to trap single guest molecules in their native form, mimicking natural strategies of molecular recognition and anticipating a new method of protein caging.

## Abstract (web summary)

Current strategies for protein encapsulation in DNA vessels for controlled enzymatic catalysis or therapeutic delivery rely on formation of covalent complexes. Here, the authors design a system that mimics natural reversible non-covalent host–guest interactions between a DNA host and the protein DegP.

Through the physical separation of molecular species into specialized compartments, nature achieves control of matter distribution both in space and time^1^,^2^,^3^. Multienzyme complexes^4^,^5^, protein cages^6^,^7^ and bacterial microcompartments^8^ are only few examples of host systems evolved by the cell to metabolize specific guest molecules. Despite their diversity, host–guest complexes rely on non-covalent interactions between complementary shapes: a concave host surface, displaying convergent ligands, and a convex guest surface, exposing divergent binding sites^9^. Applying this basic principle, scientists succeeded in building synthetic hosts to stabilize reactive intermediates^10^, catalyse reactions^11^ and affect peptide conformations^12^. Along this line, a promising field of research deals with the design of artificial containers for protein encapsulation. Confining proteins in a chemically engineered environment may be used to modulate protein properties, allowing for example to enhance their stability, alter their function, induce conformational changes and facilitate structural elucidation by single-molecule methods. Examples reported until now are very limited and include protein immobilization within polymeric media^13^,^14^,^15^,^16^, porous solids^17^,^18^, proteinaceous capsules^19^ and organic coordination complexes^20^. Often, supramolecular chemistry methodologies have been also used as a powerful means to control protein association, organization and dynamics^21^,^22^,^23^,^24^. An emerging approach uses the DNA molecule as the building block of self-assembled objects with molecular addressability and predictable shape^25^, thus allowing to construct DNA cages^26^,^27^,^28^ and DNA origami nanochannels^29^,^30^,^31^,^32^ of variable size, mechanical rigidity and enveloping capability. Besides their unique design versatility, DNA-encapsulating agents provide a negatively charged layer that has been recently reported to enhance the stability and enzymatic activity of the internalized protein^30^, thus disclosing a primordial ‘chaperone-like'^33^ role of polyphosphate-based nanocages that promises exciting future applications.

Despite notable progresses in this field, full exploitation of synthetic DNA nanostructures as programmable protein hosts is still limited by the chemical strategies used for DNA–protein conjugation. Indeed, protein loading into DNA nanocontainers mostly relies on the chemical cross-linking of functionalized DNA strands to reactive cysteine or lysine side chains exposed on the protein surface and further hybridization of the resulting DNA–protein conjugate to complementary handles appended to the DNA–host surface. This method presents at least two disadvantages: first, a lack in the regioselectivity and stoichiometric control of protein modification, which leads to a heterogeneous mixture of products; and second, a permanent modification of the protein surface, which may interfere with molecular recognition events. Although alternative strategies have been reported^26^,^34^,^35^,^36^,^37^, all current methods require extensive protein engineering and/or manipulation with consequent alteration of protein structure and function. Therefore, a method is needed for the encapsulation of a single copy of a desired protein in its unmodified state.

Here we show an architectural strategy for the selective encapsulation of a protein guest into a synthetic DNA host, using supramolecular interactions of programmable symmetry and range-of-action. In our approach, the collective and convergent action of multiple ligands pre-oriented towards a common protein target is exploited to strengthen the binding affinity, circumventing the need for covalent protein tagging and favouring the formation of a 1:1 host–guest complex. Inspired by natural strategies of molecular recognition, DNA shells can be thus engineered to specifically identify the chemistry and geometry of a guest surface for its controlled caging with minimal human intervention.

## Results

### The DegP protein guest

**Figure 1:**
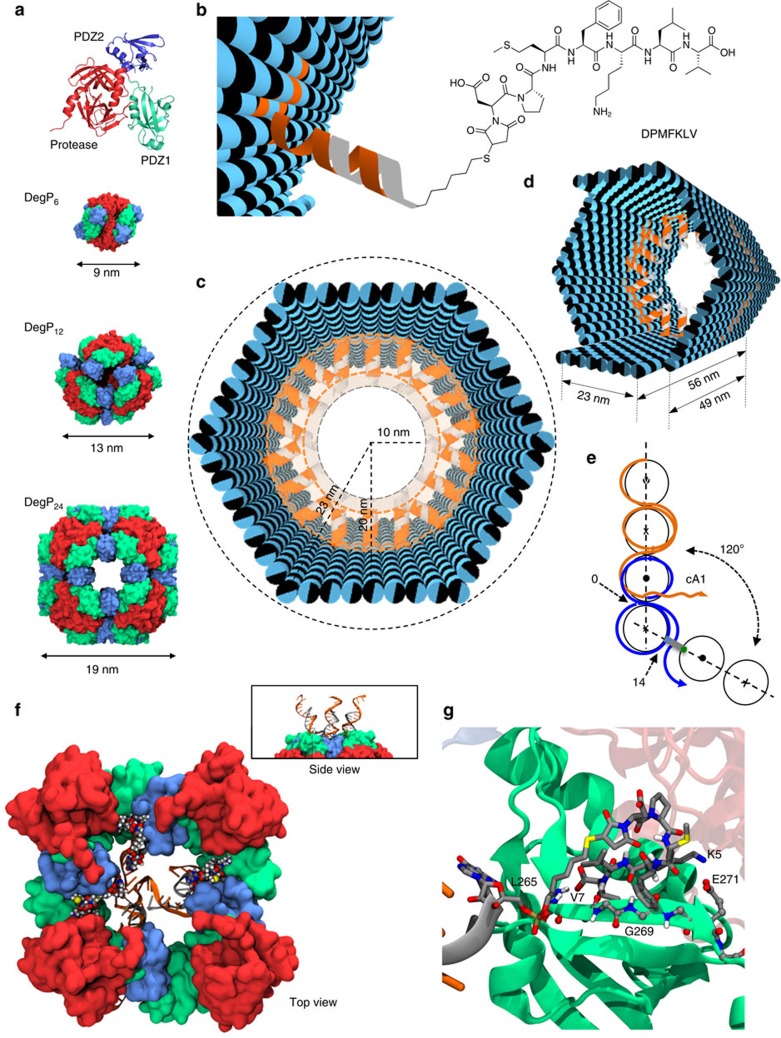
The host–guest system and the encapsulation strategy. (**a**) The DegP protein guest in its monomeric form is constituted by three domains: a protease (red), a PDZ1 (green) and a PDZ2 (blue) domain. The 6-, 12- and 24-mer DegP states have different symmetries and sizes (PDB codes are, respectively: 1KY9, 3OTP and 3CS0). (**b**) The DNA origami host is internally decorated with protruding DNA strands (orange helices) for further hybridization to complementary DNA–peptide conjugates (grey helices). (**c**) The host is made of six planar faces connected into a hexagonal prism with an edge and outer radius of 23 nm and a free inner room of *ca.* 10 nm in radius. (**d**) The host has two different lengths 49 and 56 nm, associated, respectively, to four short and two long opposite edges. (**e**) Schematic representation of the design strategy used to link adjacent faces at a fixed 120° angle, using out-of-plane crossovers. (**f**) Molecular dynamics simulations illustrate the binding of three DNA-DPMFKLV ligands to the PDZ1 domains of DegP_24_ (top view). The side view of a small region of the complex is shown in the panel. (**g**) Detailed view of the binding of the DNA-DPMFKLV ligand to the PDZ1 domain in the interior cavity of DegP_24_ (last frame of the MD simulations).

We chose DegP as the guest of our DNA origami host. DegP is a serine protease^38^,^39^ accessible in many interchangeable oligomerization states of known crystal structure^40^, ranging from the 6-mer (DegP_6_; *ca.* 250 kDa) to the 12-mer (DegP_12_; 500 kDa) till the highest 24-mer (DegP_24_; 1 MDa). Each DegP monomer consists of three domains (Fig. 1a): a serine protease domain containing the active centre (in red), a PDZ1 domain involved in substrate recognition (in green) and a PDZ2 domain that mediates stabilization of the quaternary structure (in blue). To avoid substrate digestion as well as autoproteolysis, the active site of the DegP protein was mutated by substituting the serine residue of the catalytic triad with an alanine (in the following referred to as DegP-SA; Supplementary Figs 1–3). This ensured a sufficiently long protein lifetime for experimental handling and analysis (all DegP variants used in this work are listed in Supplementary Table 1). The structural properties of the DegP protein are ideal to test our approach. Indeed, the high symmetry of the DegP oligomers ensures the equivalent display of several identical binding sites over the protein surface, allowing their targeting by a radial arrangement of convergent ligands anchored to the inner surface of the host. However, whereas symmetry clearly favours the occurrence of binding events, the selectivity of the host towards one of the three DegP forms becomes more challenging. Thus, by designing a DNA container capable of encapsulating protein guests of similar chemical affinities but different sizes and shapes, several binding scenarios can be explored and compared, eventually providing an additional parameter to control host–guest complexation, namely the geometric complementarity of the interacting surfaces.

### Design of the DNA origami host

We captured the DegP protein inside the cavity of the host by targeting its PDZ1 domains with the heptapeptide of sequence DPMFKLV (*K*_d_=*ca.* 5 μM), which acts as a specific substrate mimics (Fig. 1b)^41^. To apply multivalent binding at short intermolecular distances, we placed a layer of peptide motifs in close proximity of the binding sites exposed on the protein surface. For this purpose, the N-terminal residue of the peptide was bridged to the inner surface of the origami host through a C6 linker and a 16 bp DNA spacer (Supplementary Figs 4–6). The former provided some degree of orientational freedom for easier positioning of the ligand into the protein-binding pocket, while the latter ensured stable ligand anchoring to the host surface, resulting in a host-to-guest bridge of ∼10 nm in length (a detailed geometric model of the inner DNA–peptide corona is illustrated in Supplementary Fig. 7). Thus, to provide sufficient space for encapsulation of DegP_24_ (radius *ca.* 9.5 nm, Fig. 1a), the host cavity should have a total inner radius of ∼20 nm. This eventually required using the entire M13 scaffold for folding the hollow shape illustrated in Fig. 1c,d: a hexagonal DNA prism (in the following referred to as 6p) composed of six planar sheets connected by rigid hinges relatively oriented at 120°, with a vertex-to-centre distance of ∼23 nm (*R*_out_), an inner radius of 20 nm (*R*_in_) and a free accessible room of *ca.* 10 nm radius (*R*_free_). The prism measured 49 nm in length, except for two opposite faces with slightly longer edges (56 nm), which offered a useful topographical feature for atomic force microscopy (AFM) characterization (Fig. 1d; molecular models and design details are provided in Supplementary Figs 8a and 9). The interior of each origami face has been decorated with up to three DNA-protruding arms of identical sequence (cA1, orange helices in Fig. 1b–d), leading to a maximum of 18 attachment points radially distributed around the centre of the cavity. Such handles served for functionalization of the host cavity with the peptide ligands previously conjugated to a partially complementary 16 bases long DNA sequence (A1, in grey in Fig. 1b–d).

We chose a regular hexagonal shape of the DNA cage to attain a large quasi-radial distribution of peptide ligands, compatible with the symmetry of the PDZ1 domains in all three oligomeric forms. To improve the mechanical stiffness of the structure, we imposed dihedral angles of 120° (or 240°) through out-of-plane crossovers between the helices of adjacent edges (Fig. 1e, Supplementary Fig. 10 and Supplementary Discussion). This allowed to realize host chambers with a predictable orientation of protruding arms (PAs) in respect to their inner cavity, which is of course of upmost importance for encapsulation purposes. In the following, a fixed angle of 120° between adjacent origami faces will refer to a configuration of the host whose PAs point towards the inner cavity (indicated as 6p^120^), a 240° angle will be instead associated to PAs directed outwards (6p^240^), and finally, edge-to-edge connections by flexible T-hinges (180°) will refer to a structure with an undefined direction of the arms (6p^180^). In addition, the number of protruding arms available for attachment of peptide ligands will be indicated as 0cA1, 6cA1 or 18cA1, corresponding, respectively, to zero, one or three PAs per origami face (Supplementary Fig. 11a). Successful achievement of the desired structures has been proven by gel electrophoresis analysis and AFM characterization of biotin-modified constructs loaded with streptavidin molecules (Supplementary Figs 12–19). To better investigate the role of the inner cavity size, we constructed another channel of different geometry, that is, a triangular prism (3p; Supplementary Figs 8b, 11b, 20 and 21), having the same surface area but an inner spherical volume of ∼9 × 10^3^ nm^3^, which is about 3.5-fold smaller than the volume included within the 6p structure (32 × 10^3^ nm^3^; the nomenclature of all structures used in this work is given in Supplementary Table 2).

### Structural studies and theoretical considerations

In the three oligomerization states discussed here, DegP monomers are arranged around a central hole of increasing size (Fig. 1a). In DegP_6_, this arrangement places the PDZ1 domains pointing towards the solvent. The PDZ1 domains in DegP_12_ are instead located at the entrance of the central hole and are grouped in units of three arranged in a tetrahedral orientation. Conversely, DegP_24_ features a cube with six possible entrances, formed each by four PDZ1 domains. In both DegP_12_ and DegP_24_ the binding sites of the PDZ1 domains are oriented towards the inner cavity of the protein. Our Molecular Dynamics (MD) simulations suggest that up to three DNA helices can point into a quartet of PDZ1 domains without compromising the binding of the incoming peptide (Fig. 1f, Supplementary Fig. 22 and Supplementary Table 3). Thus, for the 6p construct, the presence of three ligands per origami face, that is, a total of 18 ligands, should provide the maximal probability of binding. Peptide interaction to the PDZ1 domain is driven by the placement of the side chain of V7 in the hydrophobic pocket of the PDZ1 and the hydrogen bonds established between the backbone atoms of the peptide (residues 5 to 7) and the corresponding residues of the PDZ1 domain (residues 265–269, Fig. 1g). Although the size and high degree of rotational symmetry of the host should guarantee best geometric matching with a single copy of the 24-mer, alternative binding situations cannot be excluded. Atomistic geometrical models show indeed that all DegP forms, although with distinct space-filling capabilities, can be hosted inside the DNA 6prism according to three binding modes and that even more than one protein molecule could in principle fill the cavity (Supplementary Figs 23–33). The scenario becomes even more complicated when considering the diffusion properties of the protein cargos. Assuming that *r*_prot_ is the radius of the encapsulated protein and *R*_free_ is the radius of the available space within the cavity, the flux (**I**) of protein particles traversing the host in a typical experiment (30 μl of a 20 nM origami solution, at room temperature and in aqueous buffer) is given by **I**=166 *R*_free_/*r*_prot_ (particles per s; Supplementary Note 1). This implies that the number of protein particles diffusing through the host channel per unit of time is proportional to the inverse of protein size in a 4:2:1 ratio, for the 6-, 12- and 24-mer, respectively. In other words, from a merely statistical perspective, smaller DegP oligomers should have a higher chance of diffusing and thus binding inside the DNA origami host. On the other hand, filling the host cavity with a single copy of the largest oligomer should maximize the efficiency of binding due to better host–guest interface matching (estimated values of **I** for different DegP oligomers/host pairs are reported in Supplementary Table 4).

### Gel electrophoresis analysis of protein encapsulation

**Figure 2:**
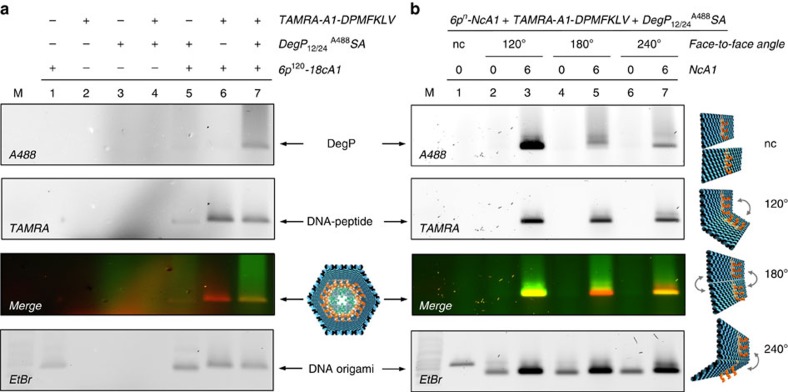
Gel electrophoresis analysis of DegP_12/24_^A488^ SA binding to the DNA host. (**a**) Analysis of the single components of the complex (lanes 1–3, corresponding, respectively, to the DNA origami host, the TAMRA-labelled DNA–peptide conjugate and the DegP protein) as well as of their mutual interactions (lanes 4–7) indicates specific DNA–protein binding only in presence of the peptide (lane 7). (**b**) Binding of DegP_12/24_ to the 6p construct occurs only in presence of PAs (NcA1=6 in lanes 3, 5 and 7) and is dependent on their convergent (120°), randomly oriented (180°) or divergent (240°) arrangement. Only a convergent design of ligands (lane 3) leads to satisfactory yields of binding, whereas undefined (lane 5) or divergent (lane 7) ligand orientation is poorly efficient (yields are in a 8:1.4:1 ratio). As control, a DNA structure lacking the face-to-face connections (nc) has been used (lane 1). Lanes M contained 1 kbp DNA ladder (Roth). The DNA origami structures migrate between the 1.5 and 2.0 kbp bands of the ladder. Gel running conditions: 0.75% agarose in 1 × TBEMg buffer, 4 °C, 3 h at 80 V. Gel imaging was performed with a Typhoon FL900 upon illumination at selected wavelengths to allow detection of the protein (Alexa488), peptide ligand (TAMRA) and DNA (upon ethidium bromide staining). Full gels are shown in Supplementary Figs 34 and 43.

Successful and specific binding of DegP_12/24_ to the DNA origami host has been demonstrated by agarose gel electrophoresis (Fig. 2a). The co-localization of the fluorescence signals associated, respectively, to the DegP_12/24_^A488^SA (labelled at exposed lysine residues with Alexa488 fluorophores) and the DNA host, functionalized with 18 protruding arms in its inner cavity (6p^120^-18cA1), occurs only in presence of the TAMRA-labelled peptide ligands (cfr. lanes 5 and 7; see also Supplementary Fig. 34). It is worth noting that, before protein addition, all DNA origami structures were purified to remove the excess of staples in solution as well as the unbound DNA–peptide conjugates. This ensured that protein–ligand interaction could occur only within the cavity of the host. Functionalization of the DNA origami structure with the peptide ligands led, as expected, to a slight decrease in the migration rate (Fig. 2a, cfr. lane 5 with lanes 6 and 7 in ethidium bromide staining). Interestingly, binding of the protein to the peptide-modified origami did not result in any remarkable gel mobility shift, although their co-migration clearly proved a mutual interaction (Fig. 2a, cfr. lane 6 with 7; see also Supplementary Discussion). Binding specificity was also confirmed for the DegP_6_^A633^SA (labelled at genetically introduced cysteine residues with Alexa633 fluorophores) and the wild-type form DegP_6_^A488^WT (respectively, in Supplementary Figs 35 and 36), indicating the general validity of our approach independently of the protein oligomerization state and labelling chemistry. We also observed that the efficiency of protein loading increases with the number of peptide ligands available within the inner cavity of the host (Supplementary Fig. 37) and that this reaches a maximal value already after *ca.* 3 h incubation time (Supplementary Fig. 38).

Contrarily to DegP, the electrophoretic mobility of other host/protein complexes of comparable total mass was expectedly slower than that of the unloaded DNA host (Supplementary Figs 12 and 39; a list of ligand/protein pairs analysed in this work is given in Supplementary Table 5). We attributed the ‘anomalous' migration rate of the DNA/DegP complex to a partial suppression of the positive net charge of the protein upon labelling: full encapsulation of DegP into the DNA host would eventually lead to a negligible variation of the surface charge density of the origami structure and thus to its almost unaffected electrophoretic mobility (see also Supplementary Discussion). To verify this hypothesis and better understand the role of the protein surface charge on its binding to the DNA origami host, we performed a series of loading experiments, in which unlabelled DegP was incubated at different pH values (Supplementary Fig. 40) or small ‘charge-quenchers', that is, lysine-specific molecular tweezers^42^, have been added to the reaction mixture (Supplementary Fig. 41). The results indicate that screening the positive charges exposed on the protein surface notably enhances the yield of protein encapsulation and simultaneously reduces unspecific electrostatic interactions with the negatively charged DNA host, thus enabling the specificity of peptide ligand binding to emerge. This leads to the formation of a well-defined product with a migration rate similar to the unloaded DNA origami structure. Similar results were obtained for encapsulation of DegP_12/24_^A488^SA into a 3p host construct (Supplementary Fig. 42), confirming again the general applicability of our approach. In this case, however, the protein size is larger than the inner free room of the host (Supplementary Fig. 7b), implying that a partial deformation of the DNA cage must occur to allow accommodation of large guest molecules.

We then investigated the effect of distinct face-to-face connections on the loading of DegP_12/24_ both in absence and presence of protruding arms (Fig. 2b). Again, protein binding was observed only in presence of the PAs, which are complementary to the DNA–peptide conjugates, thus confirming the specificity of the binding interaction (Fig. 2b, lanes 3, 5 and 7). Comparing the bands associated to the DNA–protein complexes in the different connection designs, we noticed that, although the constructs displayed an almost identical DNA and TAMRA content, the yield of protein loading was extremely dependent on the angle imposed between adjacent faces. Gel analysis using ImageJ (Supplementary Fig. 43) led to yields of protein binding in a 8:1.4:1 ratio, for the 120°, 180° and 240° designs, respectively (Fig. 2b, lanes 3, 5 and 7). The higher loading efficiency observed for the host structure whose PAs are directed inwards (120°) suggests that convergent ligands within a restricted environment promote protein caging, presumably as a consequence of their high local concentration and spatially coordinated interactions towards a common target. This condition is indeed not met when the PAs are oriented either stochastically (180°) or outwards (240°). In thermodynamic terms, this implies that in our system the entropic cost of loading the protein inside the cage must be compensated by a larger energetic gain of ligand binding. This hypothesis is confirmed by the almost negligible role of ligands orientation in the biotin/streptavidin system, where the high affinity of binding ensures formation of the complex already at substoichiometric ligand concentrations (Supplementary Fig. 12).

**Figure 3:**
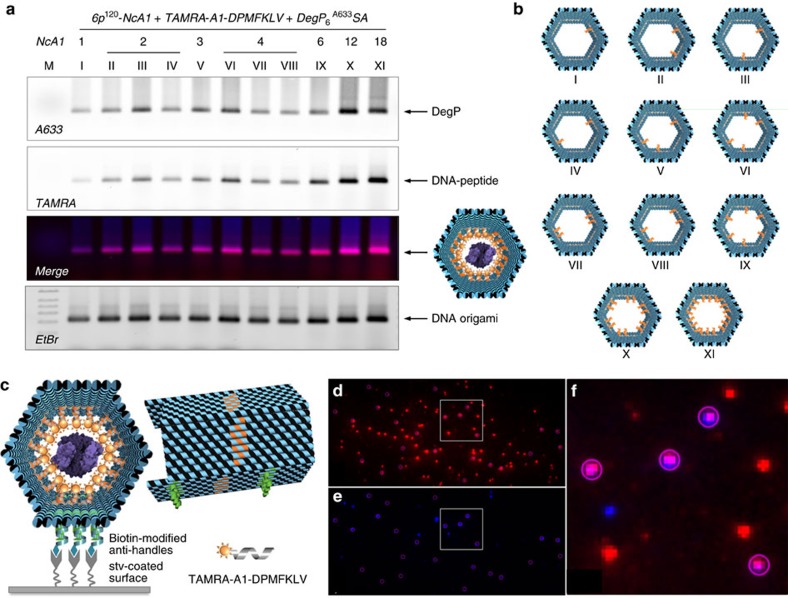
Binding of DegP_6_^A633^ SA to diverse 6p^120^ constructs. (**a**) Agarose gel electrophoresis characterization of the binding of DegP_6_^A633^SA (labelled at genetically introduced cysteine residues with A633) to diverse 6p^120^ constructs, differing in the number (NcA1; from 1 to 18) and spatial arrangement of the ligands within the cavity (constructs I to XI, **b**). The results indicate successful binding for all constructs, with maximal efficiency for a radial distribution of ligands (constructs X and XI). Lane M contained 1 kbp DNA ladder (Roth). The DNA origami structures migrate between the 1.5 and 2.0 kbp bands of the ladder. Gel running conditions: 0.75% agarose in 1 × TBEMg buffer, 4 °C, 3 h at 80 V. Gel imaging was performed with a Typhoon FL900 upon illumination at selected wavelengths to allow detection of the protein (Alexa633), peptide ligand (TAMRA) and DNA (upon ethidium bromide staining). (**c**) Single-molecule fluorescence characterization of the 6p construct, bearing 18 convergent PAs hybridized with TAMRA-tagged peptide ligands and loaded with a DegP_6_^A647^SA protein. Molecules were immobilized on a coverslip surface and were measured using TIRF microscopy. TAMRA (red spots in **d**) and Alexa647 (blue spots in **e**) detection channels have been overlapped, indicating clear co-localization of the two species (violet spots showing energy transfer from the donor to the acceptor fluorophore in **f**, which shows a zoom-in view of the highlighted region in **d**,**e**).

To explore the effect of ligands multiplicity, we prepared distinct host constructs differing in the number and spatial arrangement of PAs within the cavity (Fig. 3a,b). Two facts emerged: first, protein loading, although with low yields, occurred already in presence of one single ligand (Fig. 3a,b, construct I). Second, the efficiency of protein binding was proportional to the number of internalized peptide ligands and assumed a maximal value for a radial distribution of 12 or 18 ligands (Fig. 3a,b, constructs X and XI). Altogether, these results suggest that protein confinement within an artificial environment allows for molecular recognition events to take place even at nanomolar concentrations and that host–guest complexation is favoured by a higher number of ligands radially distributed around the protein surface. One should note that the protein sample used in our experiments contained a mixture of DegP_12_ and DegP_24_, which are extremely difficult to isolate in pure form (Supplementary Fig. 3). Although not affecting our conclusions, we employed single-molecule techniques to discriminate between the different oligomerization states and investigate more in detail the loading capability of our DNA host system in relation to the size of the encapsulated guest.

### Single-molecule analysis of protein encapsulation

**Figure 4:**
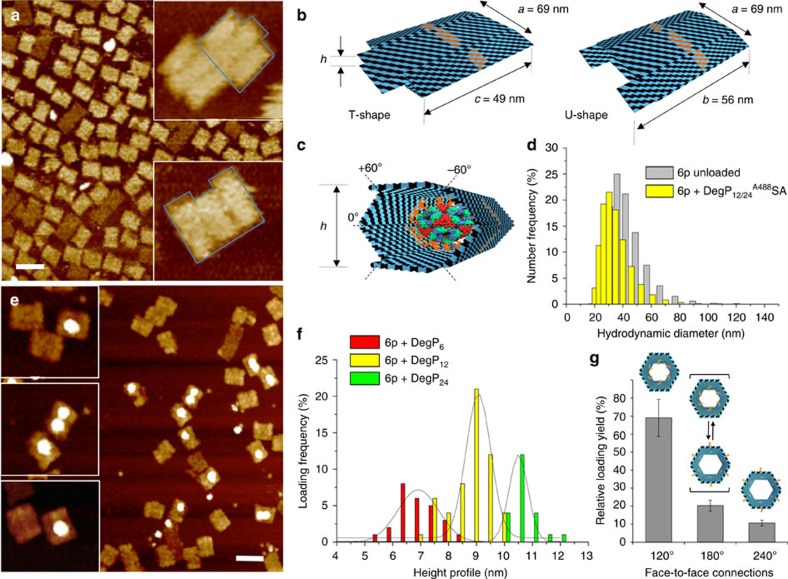
AFM characterization of the unloaded and DegP-loaded host. (**a**) The unloaded host deforms during AFM imaging in air, leading to a double-layered structure with one of two possible shapes: either a T or a U shape (**b**). Whereas the former results from the bending of the structure along the symmetry axis indicated as 0°, the latter is obtained from compression of the structure along the ±60° axes (**c**). Width and length of the obtained structures are as expected when compressing a correctly formed construct. (**d**) The hydrodynamic size distribution of the gel-purified DNA origami host either unloaded (grey bars) or loaded (yellow bars) with DegP_12_ protein was measured by dynamic light scattering, demonstrating correct formation of the hollow structure in solution. (**e**) Loading of the host with DegP protein mostly results in the appearance of single brighter dots in the centre of the structures, thus indicating successful protein binding in a 1:1 ratio. Scale bars, (**a**,**e**) 100 nm. (**f**) Analysis of the height profile of the structures revealed three distributions centred at *ca.* 7, 9 and 10.5 nm, corresponding, respectively, to encapsulation of the 6-, 12- and 24-mer (red, yellow and green bars, respectively). Preferential binding selectivity was observed for the 12-mer (more than 50% of the whole population). (**g**) Systematic analysis of the loading yield revealed most efficient protein encapsulation for a convergent design of multiple ligands (120°). Error bars indicate s.d.'s.

**Figure 5:**
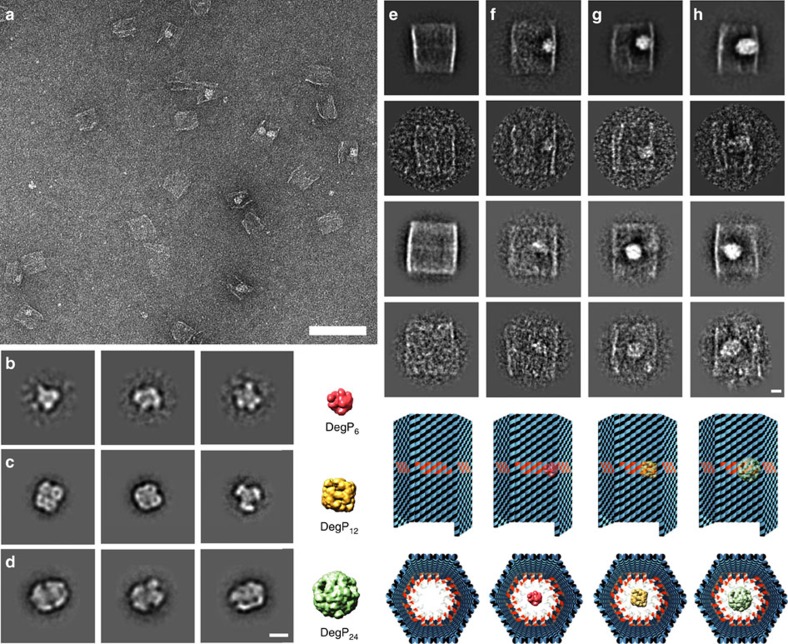
Negative stain EM of DegP and DegP-loaded DNA hosts. (**a**) Representative digital micrograph area of negatively stained DegP_12_ and DegP_24_-loaded DNA cages. Scale bar, 100 nm. Representative class averages, each containing ∼50–100 particles, for DegP_6_ (**b**), DegP_12_ (**c**) and DegP_24_ (**d**). Scale bar, 10 nm. The simulated 3D models from the respective crystal structures downfiltered to a resolution of 15 Å are also indicated. PDB-ID codes for DegP_6_ (**b**), DegP_12_ (**c**) and DegP_24_ (**d**) are, respectively, 1ky9, 2zle and 3cS0. Two-dimensional analysis of empty (**e**), DegP_6_ (**f**), DegP_12_ (**g**) and DegP_24_ (**h**) loaded DNA origami hosts bearing 18 convergent PAs in their cavity (6p^120^-18cA1). Representative class averages, each containing ∼25–100 particles, and raw particle images of the corresponding classes are reported, together with a model of each construct, both in top and front view. Scale bar, 10 nm.

We performed single-molecule characterization of gel-purified compounds using total internal reflection fluorescence (TIRF) microscopy (Fig. 3c–f), AFM (Fig. 4) and transmission electron microscopy (TEM, Fig. 5). A 6p DNA host was modified with biotin handles appended on the outer surface of one of its six faces for further immobilization on a streptavidin-coated glass support (Fig. 3c). TIRF imaging of the gel-purified complexes obtained upon loading the host with TAMRA-labelled peptides and DegP_6_^A647^SA yielded fluorescence spots from both the TAMRA donor (Fig. 3d) and the Alexa647 acceptor (Fig. 3e) following donor excitation. Binding of the two partners resulted in the observation of energy transfer from the donor to the acceptor fluorophore (Fig. 3f), thus clearly indicating that the two binding partners were in close proximity.

AFM imaging in air led to deformation of the origami structures, probably as a consequence of dehydration effects and mechanical forces applied by the AFM tip during probe scanning (Fig. 4a). In absence of a loaded cargo, compression of the hexagonal prism along one of the three possible symmetry axes resulted in formation of rectangular-shaped structures with three-faced features and a height profile of ∼3.1 nm, that is, *ca.* twofold the height of a monolayer of double helices (Fig. 4b). Two kinds of rectangular shapes could be identified: a T-shape (35%) and a U shape (65%), caused by the bending of the prism, respectively, along the axis at 0° or relatively oriented at ±60° to it (Fig. 4c). Despite the observed flattening, formation of the correct structure was almost quantitative with experimental values of size deviating less than 3% from the theoretical expectations (Supplementary Fig. 44). To confirm correct three-dimensional (3D) folding of the structures in solution, we performed dynamic light scattering (DLS) experiments on the 6p^120^-18cA1 both in absence and presence of DegP_12/24_^A488^SA (Fig. 4d). The results obtained indicate that the host structure may be approximated to a spheroidal particle of ∼40 nm in diameter (Fig. 4d, grey bars), well corresponding to the expected theoretical value of 46 nm. Protein encapsulation led to a slight decrease in the size of the loaded complex (Fig. 4d, yellow bars) that may be attributed to a partial squeezing of the structure when grasping the protein guest, thus corroborating proper internalization.

AFM analysis of the host–guest complexes disclosed in most cases binding of one single-protein molecule. A representative image for encapsulation of DegP_12/24_ in a 6p^120^-18cA1 host is given in Fig. 4e (additional AFM images are provided in Supplementary Figs 45–51). Statistical analysis of the AFM images allowed to distinguish three different populations with a height profile centred at ∼7, 9 and 10.5 nm, which we attribute to encapsulation of the DegP protein, respectively, in its 6-, 12- and 24-mer states (Fig. 4f, red, yellow and green bars, respectively). The data obtained demonstrate preferential binding of the host for DegP_12_ and almost a twofold lower selectivity for DegP_6_ and DegP_24_ (Supplementary Fig. 52). This suggests that, in case of similar binding affinity, higher loading efficiency occurs for guest molecules, which are small enough to diffuse through the host channel but sufficiently large to allow for short-range interactions between the exposed binding sites on the protein surface and the ligands attached to the inner side of the host. Finally, the effect of inwards, outwards and stochastically oriented PAs on the loading efficiency of DegP_6_ and DegP_12/24_ was systematically investigated, confirming again the importance of multiple convergent ligands for successful protein encapsulation (Fig. 4g). Similar results were obtained for the unloaded and DegP-loaded 3p host (Supplementary Fig. 53), confirming once again the general applicability of our approach.

Unlike AFM, TEM characterization better preserved the 3D structure of the protein and the complex (Fig. 5). A representative large view micrograph of the 6p host bound to the DegP_12/24_ is given in Fig. 5a (additional TEM images are provided in Supplementary Figs 54–57). The three protein species could be easily distinguished and revealed the expected structural features (Fig. 5b–d). Class averages, as well as representative raw TEM images and molecular models of the origami structures, either lacking or hosting the DegP in the three oligomerization states, are given in Fig. 5e–h. The DNA host appeared as a rectangular shape of ∼44 nm × 48 nm, confirming correct formation of the hexagonal prism structure (Fig. 5e and Supplementary Fig. 52). In line with the AFM results, most of the complexes analysed by TEM showed encapsulation of one single-protein molecule (Fig. 5f–h), thus confirming that, in the experimental conditions used, our approach efficiently leads to formation of 1:1 host–guest complexes. Finally, statistical analysis of the TEM images revealed preferential encapsulation of the larger oligomers (Supplementary Table 6), thus validating the capability of the DNA chamber to selectively host a desired protein guest on the basis of chemical affinity and geometric constraints.

### Preliminary studies on protein delivery and stability

**Figure 6:**
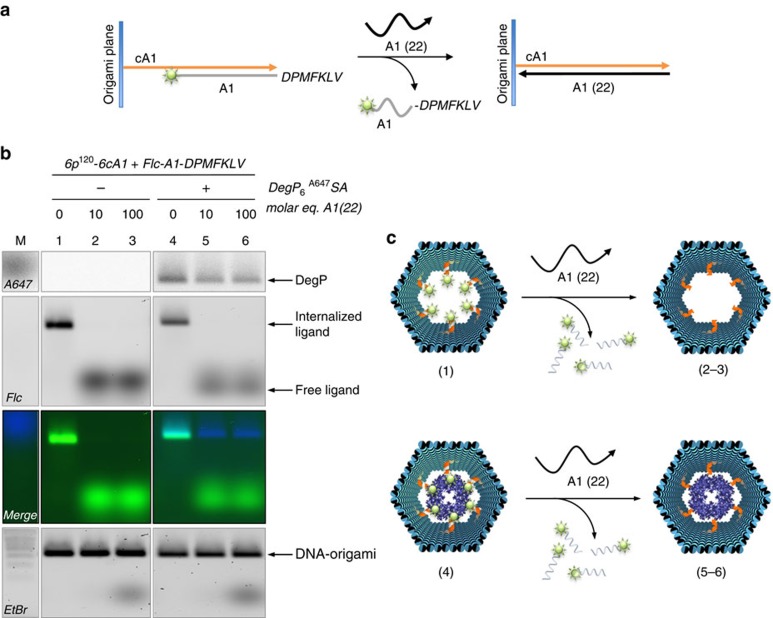
Electrophoretic analysis of single-strand displacement reactions. (**a**) The A1-peptide ligands (grey strands) are only partially complementary to the cA1 strands (orange) protruding out of the origami plane, leaving six nucleobases available for attachment of a fully complementary sequence A1(22) (black), with consequent displacement of the A1-peptide conjugate. (**b**) Gel electrophoresis analysis of purified DNA cages, either unloaded (lanes 1–3) or DegP-loaded (lanes 4–6), upon treatment with 0, 10 or 100 equimolar amounts of A1(22). The reaction was let at 30 °C overnight. The results indicate protein-trapping despite successful displacement of the peptide ligands. Lane M contained 1 kbp DNA ladder (Roth). The DNA origami structures migrate between the 1.5 and 2.0 kbp bands of the ladder. Gel running conditions: 0.75% agarose in 1 × TBEMg, at 80 V for 2 h at 4 °C. The gel was scanned with a Typhoon FLA 9000 at different wavelengths to record the presence of DegP protein (Alexa647), peptide ligands (Flc) and DNA (upon ethidium bromide staining). Full gel is shown in Supplementary Fig. 58. (**c**) Schematic representation of the products obtained in each gel lane, before and after single-strand displacement reactions.

Once the ligand-specific encapsulation of the target protein has taken place, we envision two possible applications of our method: first, the triggered delivery of the cargo upon an external signal and, second, the use of the DNA envelope to affect the stability and activity of the internalized protein. We thus applied a single-strand displacement mechanism to the inner protruding arms of the DNA host (cA1 strands) in order to induce the release of the partially complementary A1-peptide linkers after addition of fully complementary A1 sequences (Fig. 6a). The reaction was monitored by labelling the A1-peptide ligands with a fluorophore molecule: displacement of the ligands was accompanied by the disappearance of the fluorescence signal from the DNA origami cage and simultaneous appearance of a fluorescence signal in solution (Fig. 6b, lanes 1–3 and corresponding products schematically represented in Fig. 6c). Unexpectedly, removing the protein-anchoring ligands from the inner side of the DNA host did not cause the loaded cargo to escape the cage, as no free protein was found in the solution mixture and protein–DNA co-migration still persisted (Fig. 6b, lanes 4–6 and corresponding products in Fig. 6c). We therefore performed a series of experiments to test whether protein delivery could be favoured in specific conditions (Supplementary Figs 58–62). In all cases analysed, we reached the same conclusion: once the protein is bound inside the DNA host, its binding persists even upon removal of the ligands (see also Supplementary Discussion). Only by enzymatic digestion of the surrounding DNA cage we succeeded in liberating the protein back in solution (Supplementary Figs 63 and 64), although this clearly prevents the reuse of the host architecture. Thus, whereas the ligands are necessary to drive protein encapsulation, once the complex is formed, additional forces emerge that overcome both the strength and specificity of ligand binding. The reason for this behaviour is not yet fully understood; however, we presume that the electrostatic interactions taking place between the external protein surface and the inner side of the DNA origami host may play a key role.

Consistent with the model recently proposed by Yan and co-authors^30^, the high density of negative charges of the DNA cage might induce the formation of a well ordered hydration layer in the proximity of the protein surface. This compact shell of hydrogen-bonded water molecules has been shown to exhibit dynamical properties markedly different from those of bulk water, mediating long-range interactions between polar or charged groups, which are crucial for protein folding and activity^43^,^44^. This hydration layer could be responsible of the persistent encapsulation effect observed, as well as of the increased lifetime of the DegP protein when loaded inside the DNA cage (Supplementary Figs 65–67 and Supplementary Discussion). Clearly, further investigations are necessary to elucidate this point and better understand the molecular mechanisms involved; nevertheless, the premises are encouraging and the foundation is set for the construction of new biomaterials with controlled stability and enzymatic activity.

## Discussion

In this study, we have shown that merging DNA nanotechnology and supramolecular chemistry, a spatially defined envelope of weak non-covalent interactions can be placed around the surface of a single protein to drive its encapsulation in a programmable way. The arrangement of a distinct number of ligands in the vicinity of their corresponding binding sites on the protein surface can be geometrically and stoichiometrically controlled, thus allowing to modulate local concentration effects and multivalent short-range interactions of the host–guest system. This method results in the ligand-specific and size-selective encapsulation of one protein molecule on the basis of a fine compromise between optimal host–guest intermolecular distance and sufficient molecular diffusion of the guest through the host. In other words, both the chemical affinity and the geometric compatibility of the interacting species are taken into consideration for efficient binding and, most importantly, without any previous covalent modification of the protein. One can easily envisage using such structures as molecular sieves for trapping single guest molecules from a mixture of compounds of similar binding affinity, avoiding chemical conjugation strategies and thus preserving the native state of the target protein. Interestingly, we observed that the partial suppression of the DNA–protein electrostatic attractions through lysine-selective molecular tweezers enhances the specificity of host–guest recognition thus confirming the important role of the molecular shell surrounding the protein surface for DNA-caging purposes^30^. This dense hydration layer around the protein might be also responsible of the persistent trapping effect observed after removal of the ligands and could partially explain the enhanced protein lifetime detected in presence of the DNA host.

In principle, the method proposed here is applicable to every protein-recognition motif and enables to overcome the low affinity of ligand binding with a multiplicity of recognition events occurring in a confined space at predictable distances. Finally, one can foresee that modifying the inner cavity of the host with distinct ligands, multiple enzymes can be arranged into synthetic protein scaffolds, allowing, for example, to improve metabolic pathways in a programmable manner^45^. We therefore believe that our strategy may open the way to exciting new applications in which proteins can be efficiently isolated within synthetic compartments with minimal human intervention, mimicking natural strategies of host–guest complexation and enabling to affect protein properties in a completely new manner.

## Methods

### Design and characterization of the DNA origami hosts

DNA origami structures were designed with caDNAno (www.cadnano.org) and assembled using a 1:10 molar ratio between the M13mp18 ssDNA scaffold (20 nM) and each of the staple strands, in 1 × TEMg buffer (20 mM Tris, 2 mM EDTA, 12.5 mM MgCl_2_, pH 7.6). Thermal annealing was performed on a Thermocycler Mastercycler nexus gradient (Eppendorf) by decreasing the temperature from 90 to 20 °C at −1 °C min^−1^ for the 6prism construct. The 3prism structure was folded instead using the following gradient: from 90 to 45 °C at −1 °C min^−1^ and from 44 to 20 °C with −1 °C every 2.5 h. The DNA origami structures were decorated in their inner cavity with a distinct number of DNA strands of identical sequence (cA1: 5′-CTTCACGATTGCCACTTTCCAC-3′) and hybridized with A1-tagged peptides (A1: 5′-GTGGAAAGTGGCAATC-3′; peptide: DPMFKLV). Purified peptide-modified origami structures were reacted with 25 molar equivalents of DegP protein for at least 3 h (till max 12 h) at room temperature. The reaction mixtures were then analysed by agarose gel electrophoresis and desired bands were excised with a clean scalpel from an identical gel lacking ethidium bromide (to avoid structure deformation due to dye intercalation). The DNA ladder used was 1 kbp (Roth). Products were recovered using a Freeze ‘N Squeeze spin column' and directly used for AFM, TEM and TIRF microscopy characterization. All DNA sequences are provided as Supplementary Data File. Detailed experimental procedures are given in the Supplementary Methods.

### Synthesis of the DPMFKLV

The peptide ligand was synthesized according to standard solid-phase Fmoc/tBu chemistry, functionalized with a maleimide group and conjugated in solution through Michael addition with the 5′-thiol-modified A1 oligonucleotide. The 3′-terminus of the DNA strand was instead functionalized with a fluorophore (either Flc or TAMRA) for further spectroscopic tracking (Supplementary Methods).

### Genetic engineering and purification of the DegP protein

All plasmids were derivatives of pCS20 expressing wild-type *degP* with a C-terminal His tag^46^. All constructs were verified by DNA sequencing and point mutants were constructed by oligonucleotide-directed mutagenesis according to standard procedures. Protein purification was carried out under non-denaturing conditions^46^ (Supplementary Methods).

### MD simulations

The missing parameters for the MD simulations were generated using Swissparam^47^. Missing loops were built with Modeller9.10 (ref. 48). All simulations were performed using NAMD2.9 (ref. 49) with the temperature set to 300 K and a time step of 2 fs for simulation times of up to 10 ns. The CHARMM36 force field, which has shown to reproduce well the properties of DNA origami structures, was used together with the TIP3P water model^50^,^51^. The simulations were carried out in the NPT ensemble^52^. The resulting systems contained about 1.5 million atoms (more details are provided in the Supplementary Methods).

### Single-molecule TIRF microscopy

The sample was immobilized on a BSA–biotin streptavidin-coated quartz coverslip for prism-based TIRF microscopy. Fluorescence was measured using an inverted wide-field optical microscope and alternate laser excitation at 514 and 630 nm of the donor and acceptor fluorophore, respectively. Fluorescence was divided into a red and blue spectral channel (corresponding to fluorescence from TAMRA and Alexa647, respectively) and movies were recorded with an EMCCD camera (Andor, iXON 3) using a 0.5 s integration time per image and a total length of 100 s. Measurements were performed in TAE (pH 8.0) buffer (20 mM Tris-acetate and 1 mM EDTA) containing 12.5 mM MgCl_2_ and supplemented with an oxygen-scavenging system composed of 2 mM Trolox (Sigma-Aldrich), glucose oxidase (Sigma-Aldrich, 0.92 mg ml^−1^), catalase (Sigma-Aldrich, 0.04 mg ml^−1^) and β-D-(+) glucose (Sigma-Aldrich, 4.5 mg ml^−1^). Data analysis was performed by the home-made software package iSMS^53^.

### Dynamic light scattering

Size measurements based on DLS were carried out using a *Malvern Zetasizer Nano ZS*. A disposable cuvette ZEN0040 was used for the measurements. Parameters were set to a refractive index of 2.1 and an absorption of 0.184 for the sample. For the TEMg buffer a refractive index of 1.331 was entered to the software (*Zetasizer Software 7.03*). Samples were equilibrated for 30 s at 25 °C and the measurement, done using a 173° backscatter angle, was set to an automatic duration. Measurements were repeated three times for each sample.

### Atomic force microscopy

The sample was deposited on freshly cleaved mica surface (Plano GmbH) and adsorbed for 3 min at room temperature. After washing with ddH_2_O, the sample was dried under gentle argon flow and scanned in ScanAsyst Mode using a MultiMode microscope (Bruker) equipped with a Nanoscope V controller. Force (0.4 N m^−1^) constant cantilevers with sharpened pyramidal tips (ScanAsyst-Air tips, Bruker) were used for scanning. After engagement the peak force setpoint was typically 0.02 volt and the scan rates ∼1 Hz. All images were analysed using the Gwyddion software (more details are given in the Supplementary Methods).

### Electron microscopy

Protein and protein–DNA origami samples were prepared for electron microscopy (EM) characterization as previously described^54^,^55^. All images were taken with a FEI Tecnai G2 Spirit electron microscope equipped with a Lab_6_ cathode at an operation voltage of 120 kV. Digital micrographs were recorded with a 4 k × 4 k CMOS Camera F416 (TVIPS) using low-dose conditions. Single particles were boxed out manually using e2boxer^56^ and aligned using either reference-free alignment or a user-defined rectangular mask, excluding the interior of the DNA origami host. Classification was performed by k-means clustering procedures as implemented in the SPARX software package^57^. A second round of classification was then performed within each subset using the ISAC approach of the SPARX software package^58^. Details about grid preparation and image analysis are provided in the Supplementary Methods.

### Data availability

The data that support the findings of this study are available from the corresponding author on request.

## Additional information

**How to cite this article:** Sprengel, A. *et al*. Tailored protein encapsulation into a DNA host using geometrically organized supramolecular interactions. *Nat. Commun.* **8,** 14472 doi: 10.1038/ncomms14472 (2017).

**Publisher's note:** Springer Nature remains neutral with regard to jurisdictional claims in published maps and institutional affiliations.

## Supplementary Material

Supplementary Information Supplementary Figures, Supplementary Tables, Supplementary Discussion, Supplementary Methods and Supplementary References.

Supplementary Data 1 Supplementary dataset one.
