# Supplementary material for: Tailored protein encapsulation into a DNA host using geometrically organized supramolecular interactions
Source: Nat Commun. 2017 Feb 16;8:14472. doi: 10.1038/ncomms14472 (PMC5316895; doi:10.1038/ncomms14472)
Supplement: Supplementary Information — Supplementary Figures, Supplementary Tables, Supplementary Discussion, Supplementary Methods and Supplementary References. [file ncomms14472-s1.pdf]

Supplementary Figures

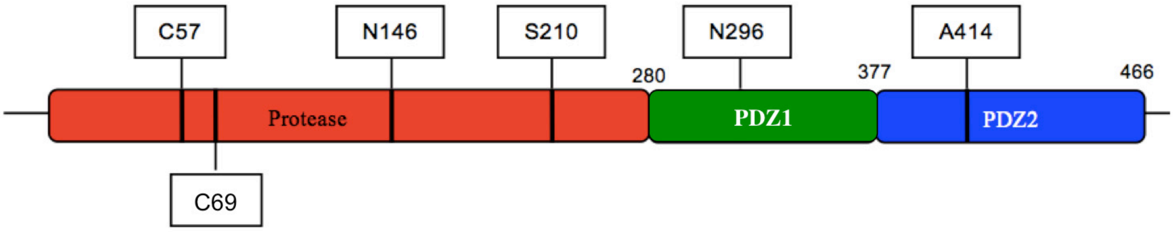

Supplementary Figure 1. Schematic representation of the genetically modified DegP gene. The catalytically inactive mutant of DegP was generated by the substitution of the serine at position 210 by alanine. The amino acids N146, N296, A414 were chosen to be mutated to cysteine for protein labeling with maleimide-activated fluorophores. To avoid mislabeling, native cysteine residues in the LA-Loop (C57 and C69) were also mutated to alanine.

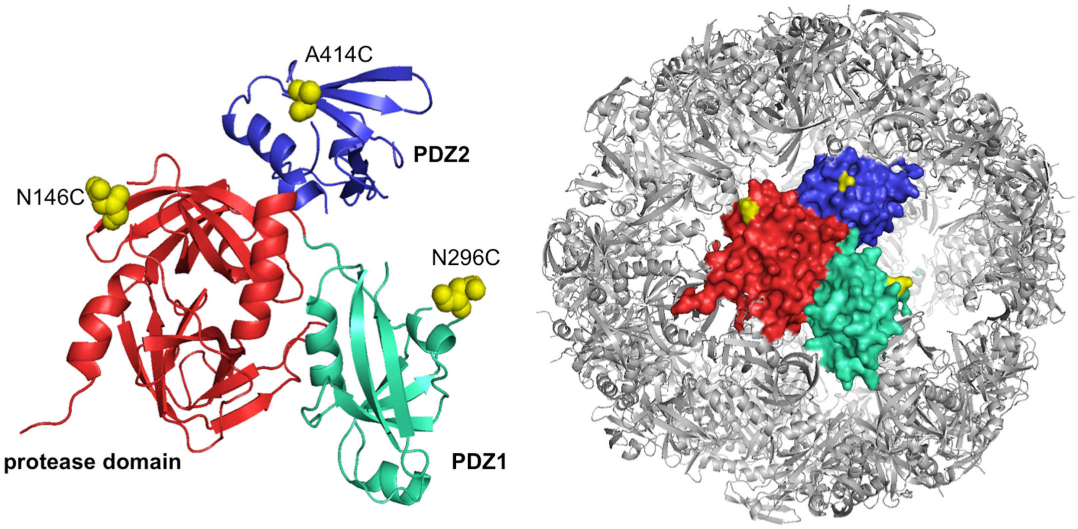

Supplementary Figure 2. Mutated amino acid residues in the DegP structure. One residue for each domain has been mutated into a cysteine for further functionalization with a maleimide-activated fluorophore. The selected residues are located on the protein surface, exposed to the solvent and far from the peptide-binding pocket of the PDZ1 domain.

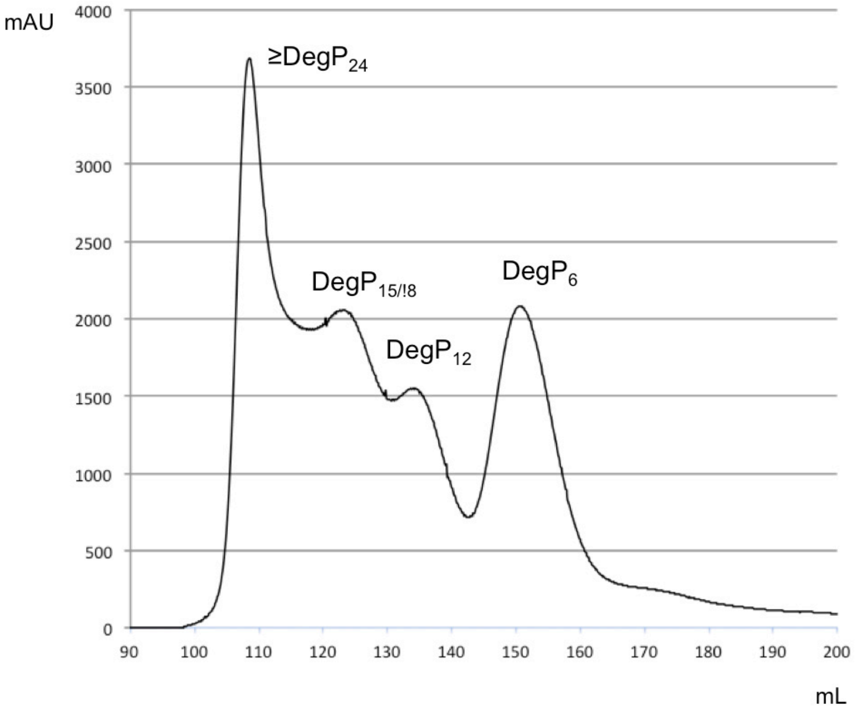

Supplementary Figure 3. Elution profile of DegP-SA. The different oligomerization states of the DegP protein have been isolated by gel filtration chromatography using a Superdex column.

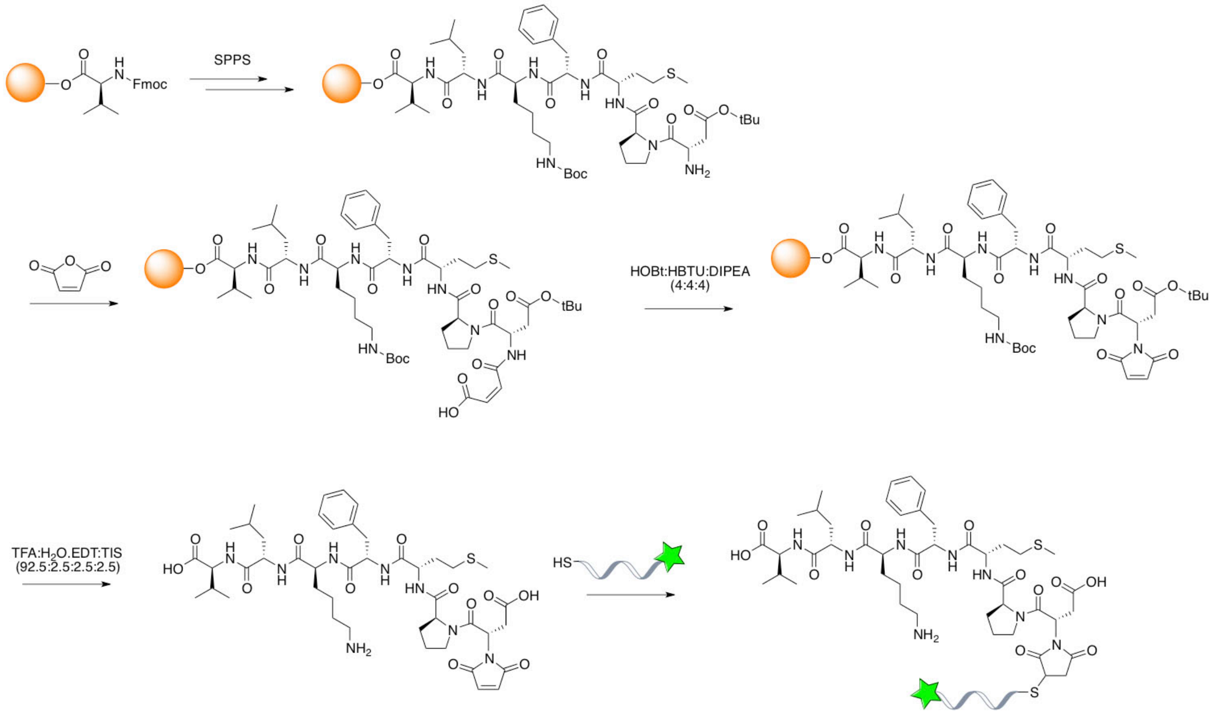

Supplementary Figure 4. Chemical route used for the synthesis of the DNA-DPMFKLV conjugate. The peptide was synthesized according to standard solid-phase Fmoc/tBu chemistry, functionalized with a maleimide group and conjugated in solution through Michael addition with a 5'-thiol-modified oligonucleotide. The 3'-terminus of the DNA strand was instead functionalized with a fluorophore (either Flc or TAMRA) for further spectroscopic tracking.

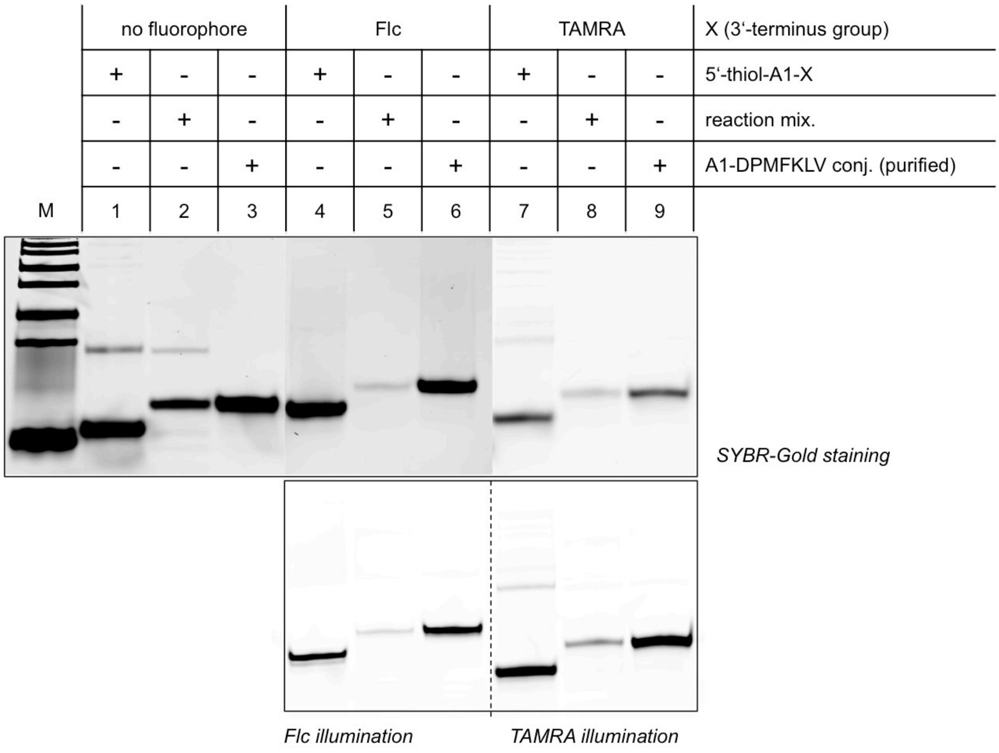

Supplementary Figure 5. Denaturing PAGE characterization of the DNA-DPMFKLV conjugates. The DNA strand (A1) used for peptide conjugation was either unlabeled at the 3'-terminus (lanes 1 to 3), or modified with a Flc (lanes 4 to 6) or a TAMRA fluorophore (lanes 7 to 9). The adduct was loaded before (lanes 1, 4 and 7) and after reaction with the maleimide-activated peptide (lanes 2, 5 and 8) and finally purified by denaturing PAGE (lanes 3, 6 and 9). Running conditions: 25% acrylamide in TBE 1X buffer at 220V for 45 minutes at room temperature, followed by SYBR Gold staining. Flc- and TAMRA-labeled products were additionally visualized under UV illumination at the appropriate wavelength. Lane M is loaded with a 10bp DNA ladder (Thermo).

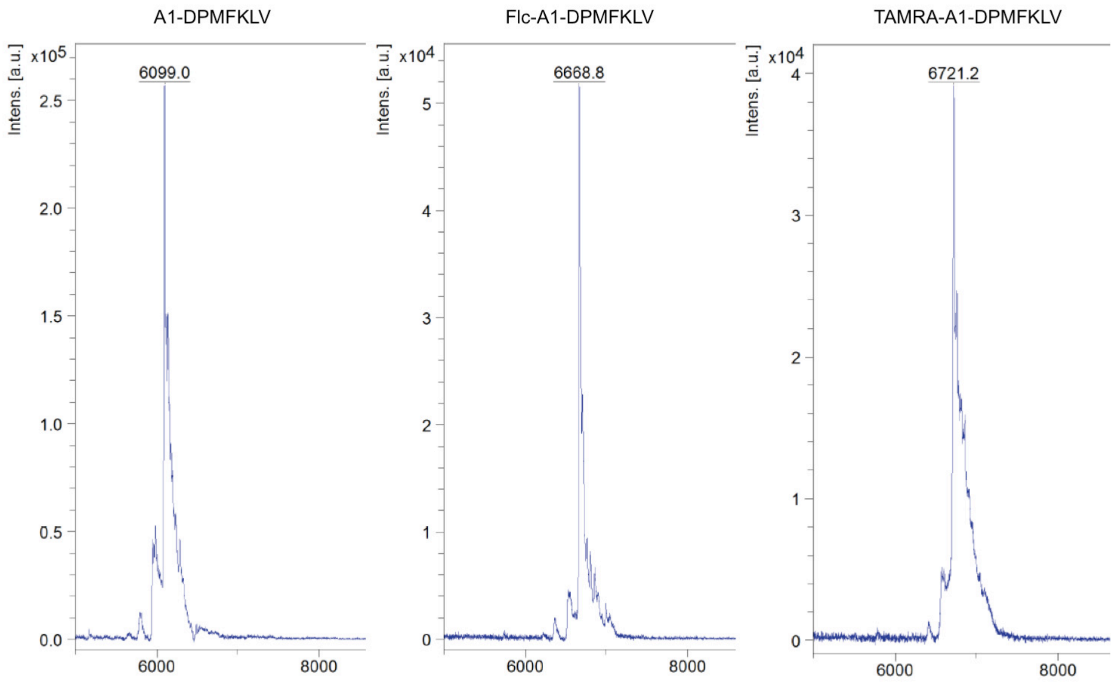

Supplementary Figure 6. MALDI characterization of the DNA-DPMFKLV conjugates. Theoretical masses are: 6099g/mol (A1-DPMFKLV), 6668g/mol (Flc-A1-DPMFKLV) and 6722g/mol (TAMRA-A1-DPMFKLV). Deviations from the experimental values are less than 0.05%.

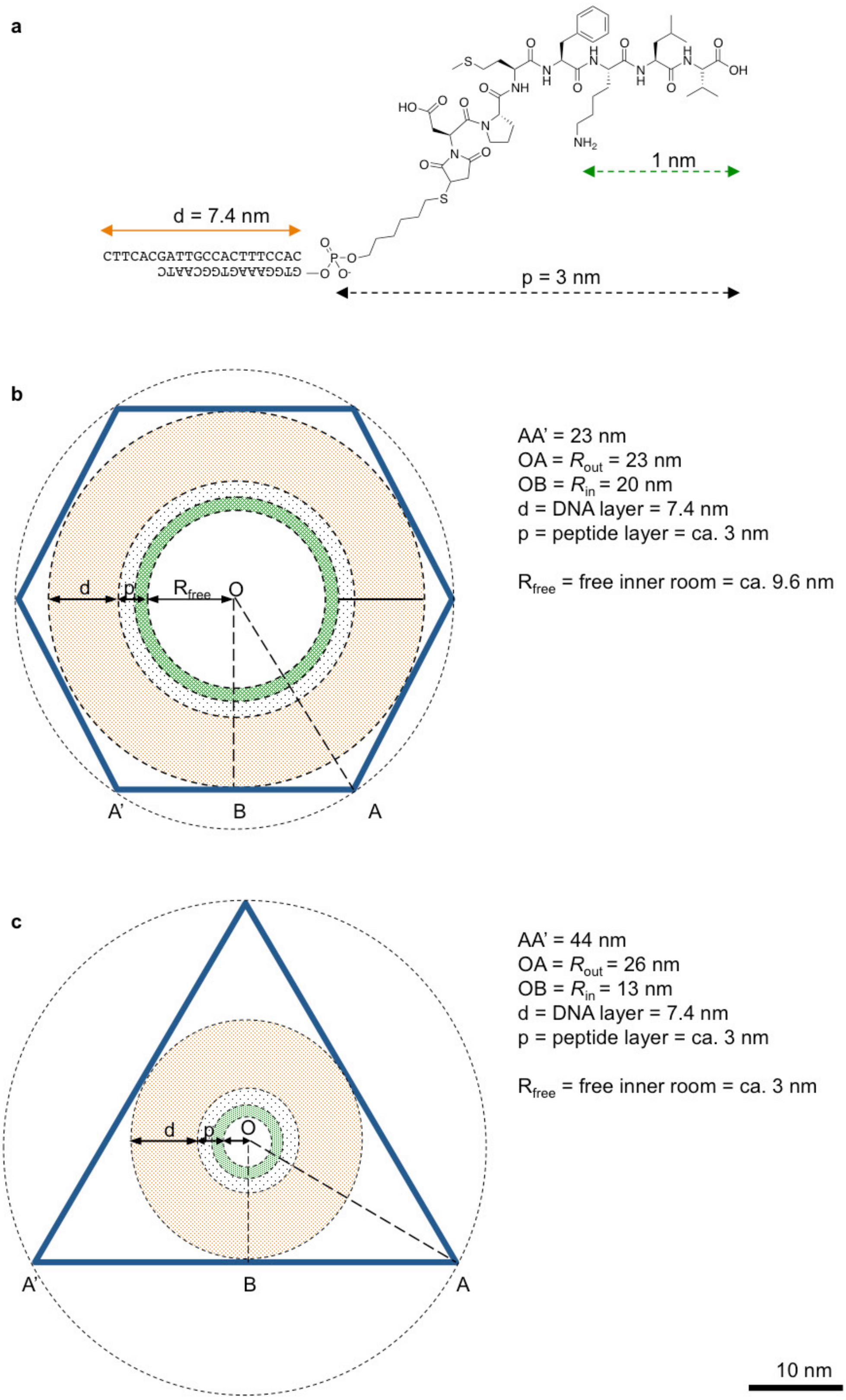

Supplementary Figure 7. Geometric models of the DNA-peptide coronas inside the DNA hosts. The inner surface of the chambers was decorated with DNA strands for further hybridization with complementary DNA-peptide conjugates. (**a**) Only the last 3 aa residues (ca. 1 nm long) interact with the PDZ1 domains of DegP. The full peptide is about 3 nm long (including a C6 spacer) and is connected to a double-stranded DNA corona of about 7.4 nm length, leading to a total host-to-guest bridge of about 10-11 nm. This leaves a free inner room of ca. 10 nm radius in the 6prism chamber (**b**) and only 3 nm radius in the 3prism chamber (**c**). The geometry of the host is expected to affect its loading capability towards a defined guest molecule.

a

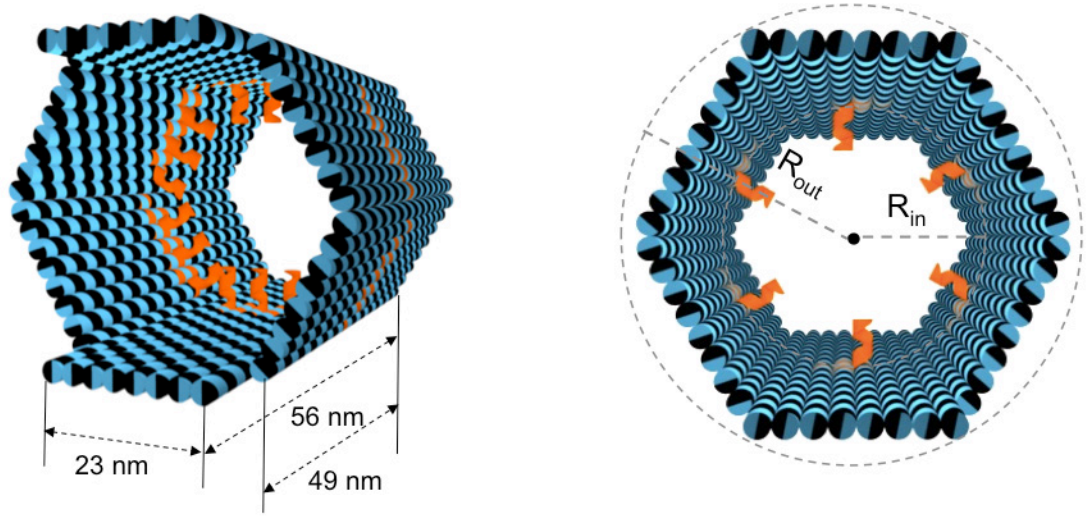

b

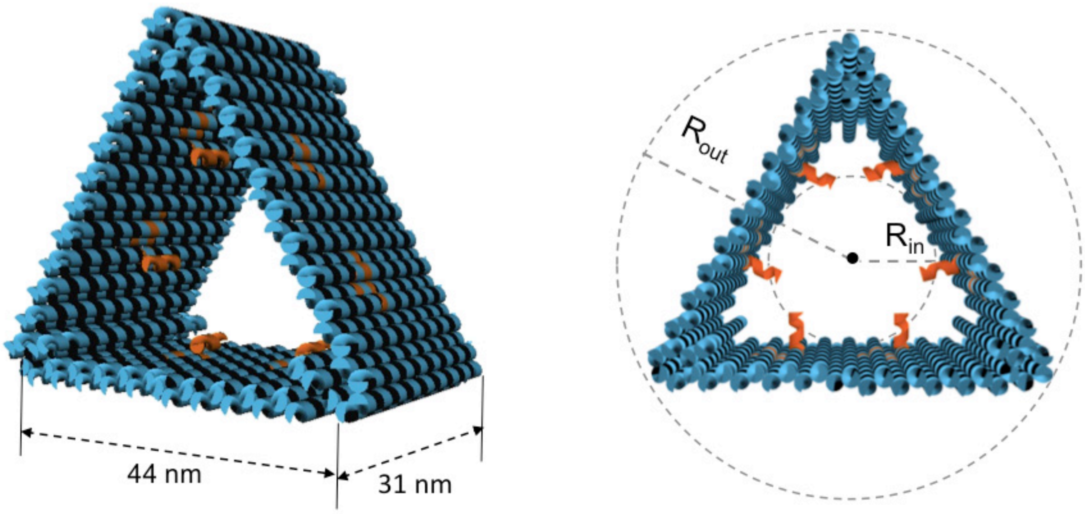

	size (WxLxH) (nm)	R_{in} (nm)	R_{out} (nm)	V_{in} (nm^3)
**6prism**	40 x 49 (56) x 46	20	23	$32x10^3$
**3prism**	44 x 31 x 38	13	25	$9x10^3$

Supplementary Figure 8. DNA host constructs used in this work. A prism with a hexagonal (**a**) or a triangular (**b**) section were prepared from the same M13 scaffold, thus leading to chambers with similar hydrodynamic radius (R_{out} = 23 nm for the 6p and 25 nm for the 3p) but extremely different inner radius (R_{in} = 20 nm for the 6p and 13 nm for the 3p). This results in a total inner volume of ca. 32 x 10^3 nm^3 for the 6p and only $9x10^3$ nm^3 for the 3p. Such a difference in the inner room of the channels is expected to affect the loading capability of the hosts towards the same molecular guests.

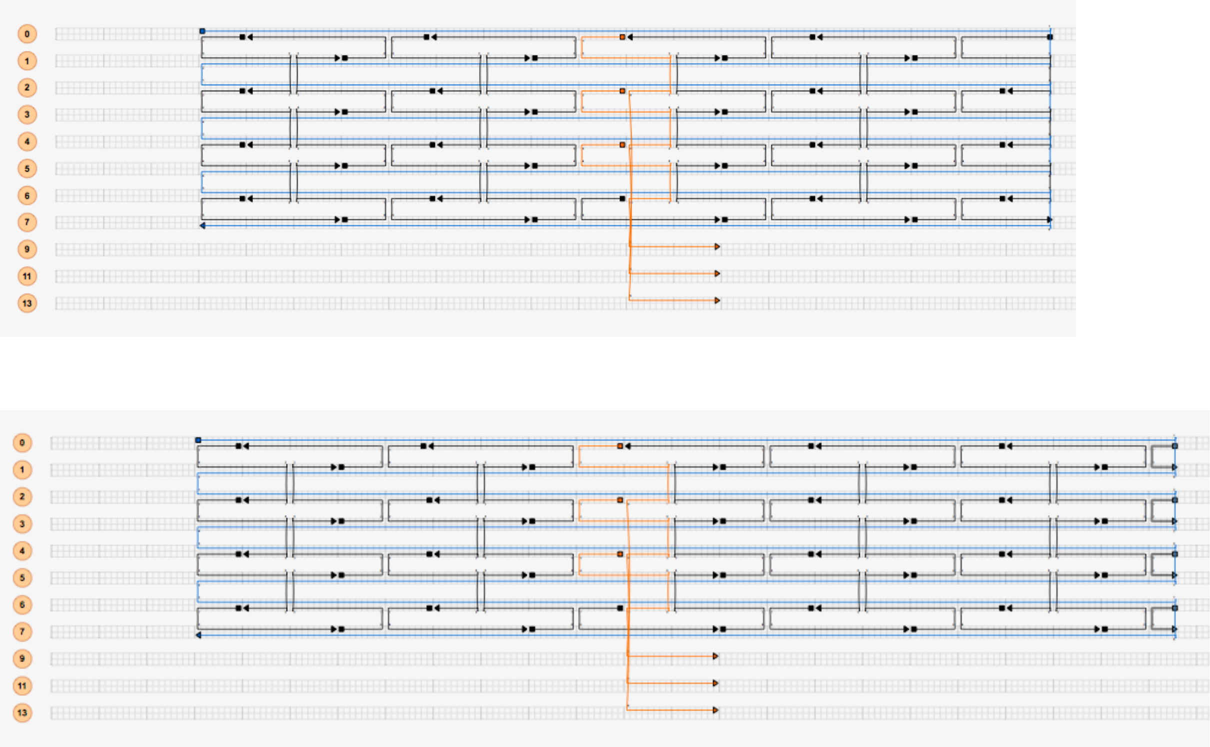

Supplementary Figure 9. Design chart of the four short and two long faces of the 6prism construct bearing 18cA1.

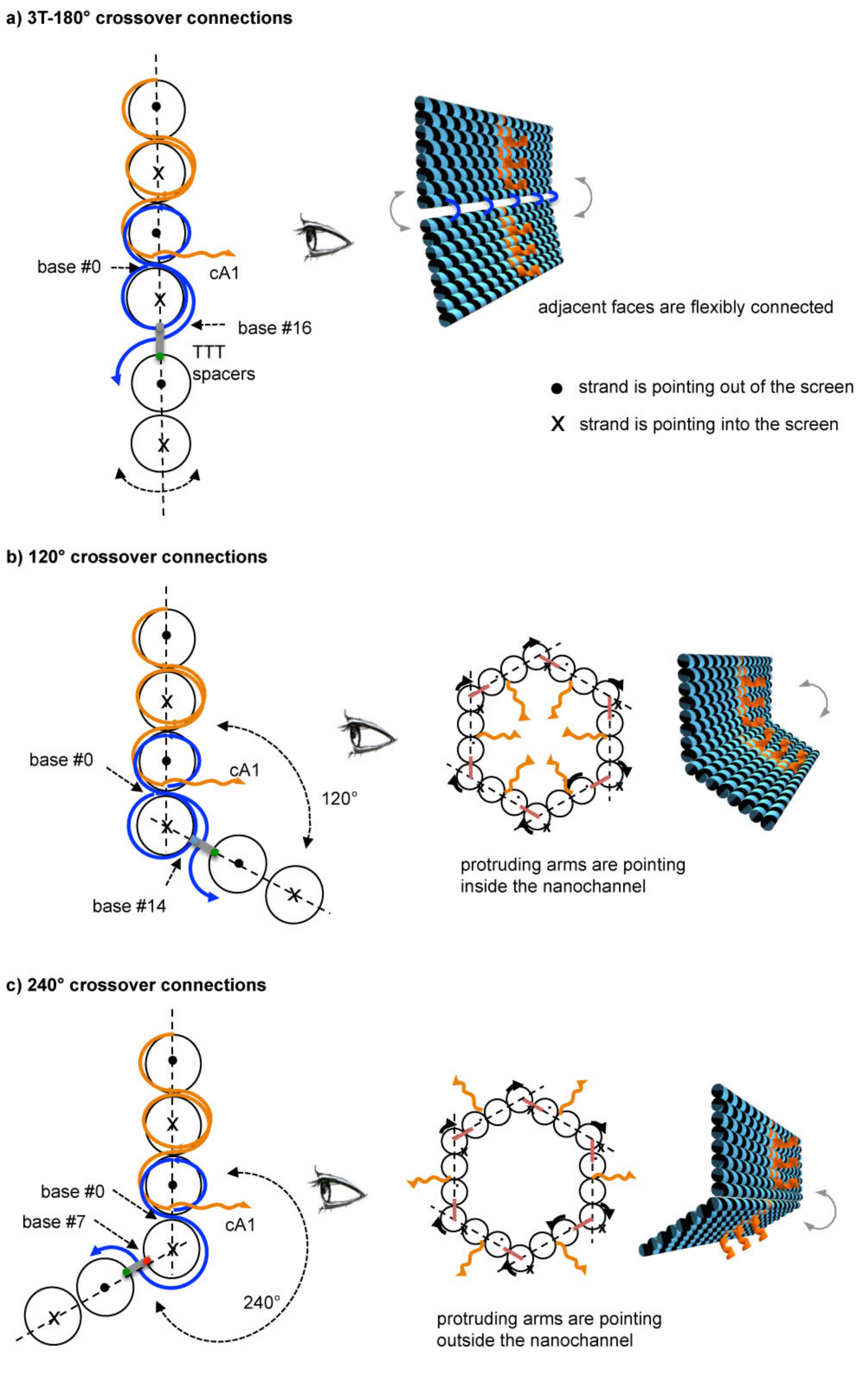

Supplementary Figure 10. Design strategy adopted to connect adjacent faces of the 6prism. The last DNA helix at the bottom of one face has been connected to the first DNA helix on top of an adjacent face through in-plane (**a**) or out-of-plane (**b**, **c**) crossovers. Starting from base #0 of the bottom helix, in-plane crossovers were placed at bases #16 (**a**) and contained 3T spacers to allow for sufficient orientational freedom of adjacent faces. Instead, placing the crossovers after 14 bp- (**b**) or 7-bp (**c**) resulted in a relative orientation of 120° or 240° between adjacent faces. In this way, the orientation of the extended staples in respect to the host cavity can be easily predicted (i.e. inwards or outwards, respectively in b and c), thus allowing to modify both sides of each origami face in a controlled fashion.

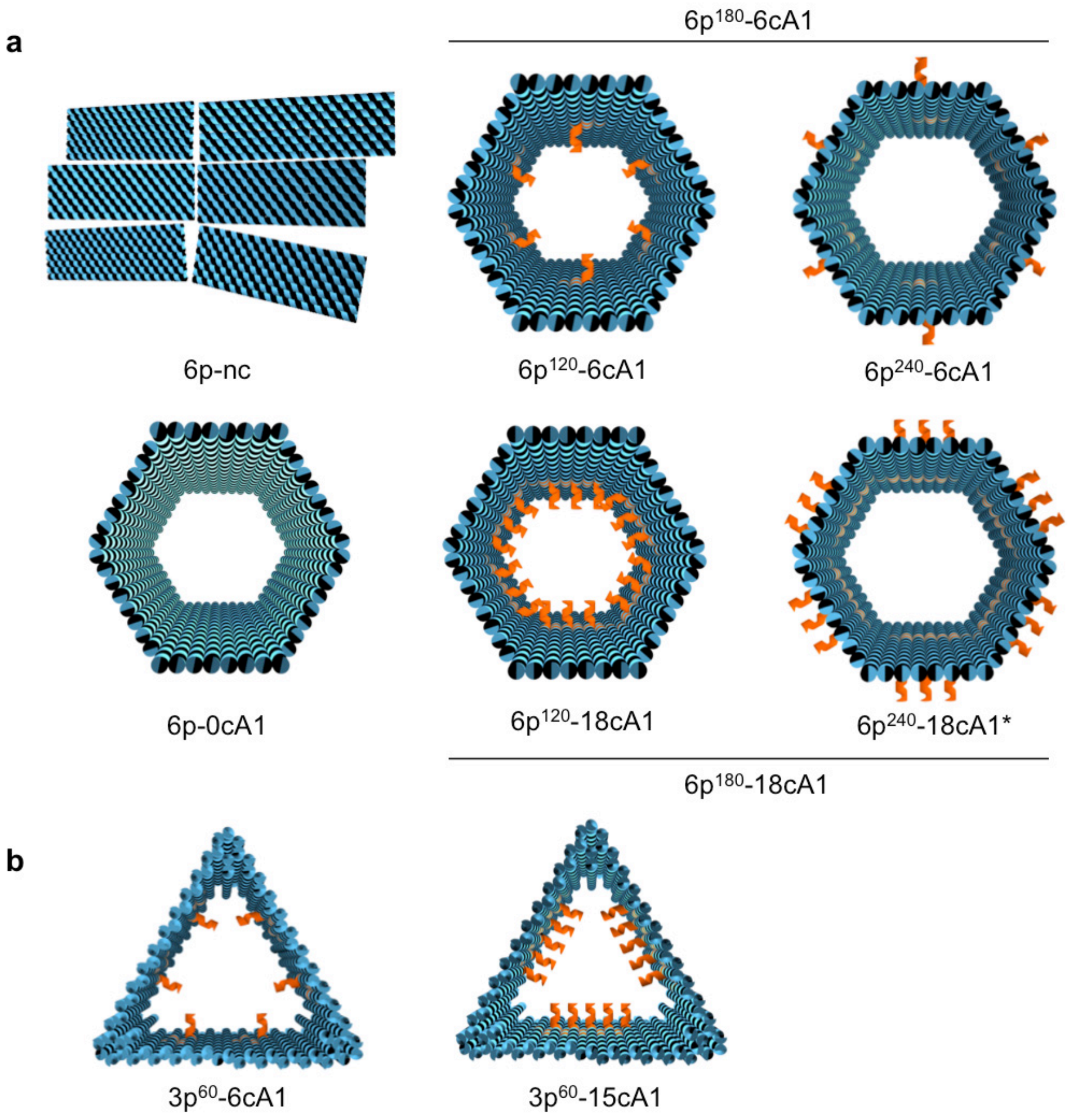

Supplementary Figure 11. Schematic representation of the 6p and 3p constructs. (**a**) The hexagonal prism (6p) has been designed with PAs pointing towards the inner cavity ($6p^{120}$) or outwards ($6p^{240}$). Edge-to-edge connections by flexible T-hinges (180°) refer to a structure with an undefined direction of the arms ($6p^{180}$). The number of protruding arms available for attachment of peptide ligands (or other A1-tagged molecules) is indicated as 6cA1 or 18cA1, corresponding respectively to one or three PAs per origami face. A 6p host lacking the PAs for ligand anchoring is indicated as 6p-0cA1. 6p-nc indicates instead a structure lacking the face-to-face connections. (**b**) The triangular prism was prepared in two possible forms, bearing either 6 or 15 inner protruding arms. Due to the different design strategy used to construct this origami structure, the face-to-face angles in the 3p construct are always fixed at 60°.

*Please note that the construct indicated as $6p^{240}$-18cA1, although present in the 180° design, was not prepared in the 240° form, as all comparative experiments on the edge-to-edge connections gave already clear results for the constructs bearing 6 protruding arms. Therefore, full comparison of constructs bearing 18 protruding arms was not considered necessary.

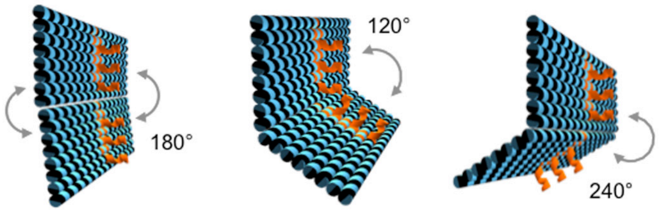

	nc	180°			120°			240°		face-to-face connection
	0	0	6	18	0	6	18	0	6	*N° cA1*
	-	-	+	+	-	+	+	-	+	*biot-A1*
	-	+	+	+	+	+	+	+	+	*STV*
M	1	2	3	4	5	6	7	8	9	

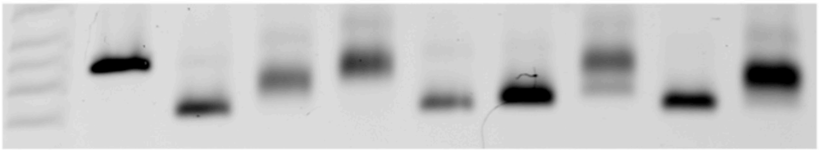

Supplementary Figure 12. Gel electrophoresis characterization of streptavidin loaded 6p hosts. The 6p constructs bearing extended staples (cA1) protruding out of the origami surface and oriented either stochastically (180°, lanes 3-4), inwards (120°, lanes 6-7) or outwards (240°, lane 9) were modified with complementary biotin-tagged DNA strands (biot-A1) and further reacted with 1.5 equimolar amounts of streptavidin. Analog constructs lacking the protruding arms in all three designs were used as controls (lanes 2, 5 and 8). The structure lacking face-to-face connections (nc: non-connected) migrated with a slower rate (lane 1), probably due to lower compactness of the open structure when compared to the analog closed forms. As expected, increasing the number of cA1 protruding arms from 0 to 6 and 18 is accompanied by a corresponding decrease in the migration rate of the complex (cfr. lanes 2-4, for the 180° design; 5-7 for the 120° and 8-9 for the 240° design). Interesting conclusions can be drawn by comparing the structures modified with the same number of cA1 (lanes 3, 6 and 9, for the 180°, 120° and 240° design, respectively). First, the migration rate of the 120°-complex (lane 6) is faster than the corresponding 180° and 240° analogs. This indicates that a convergent design of ligands effectively allows for internalization of streptavidin molecules with minimal perturbation of the surface charge of the host, thus resulting in a compact structure with migration properties similar to the unloaded DNA origami. On the contrary, in the 240° design the streptavidin molecules are placed outwards, leading to a larger complex with slower migration rate. The stochastic orientation of the PAs in the 180° design yields instead a less defined band of intermediate migration rate. In general, we can conclude that, despite the identical total molecular weight and charge, the different face-to-face connections in the 6p constructs clearly affect their structural (and gel migration) properties. Gel conditions: 0.75% agarose in 1X TBEMg, at 80V for 2:15h at 4°C. Lane M contained a 1 kbp DNA ladder. The unmodified DNA origami structures (lanes 2, 5 and 8) migrate between the 1.5 and 2.0 kbp bands of the ladder.

All products, except lanes 4 and 7, have been analyzed by AFM, confirming the successful achievement of the desired structures (see below Supplementary Figs. 13-19).

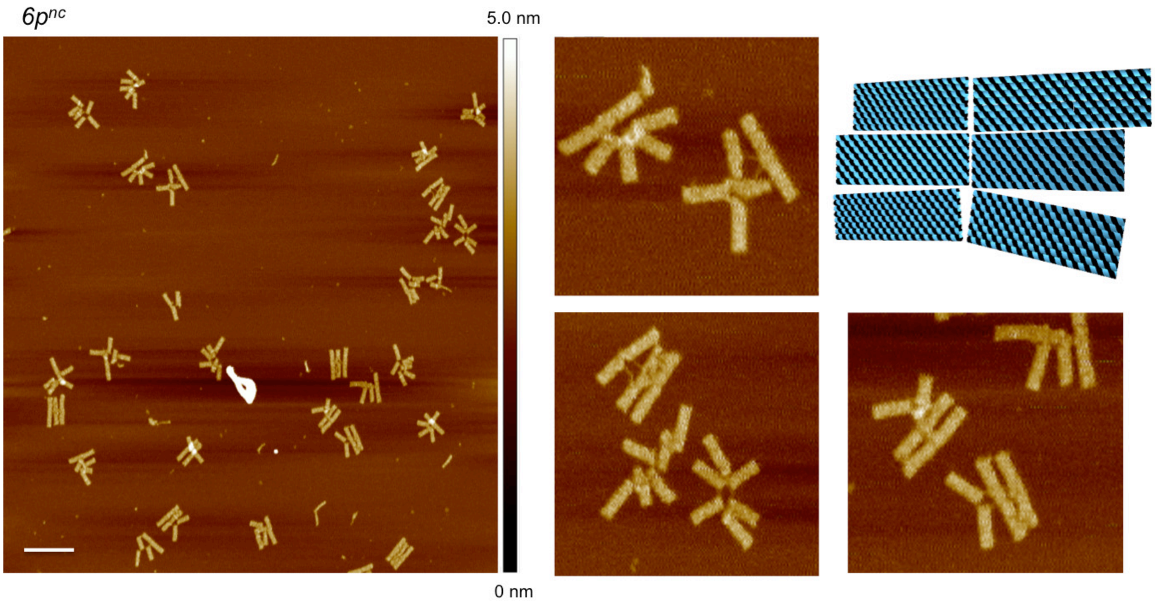

Supplementary Figure 13. AFM imaging of the 6p lacking face-to-face connections. Scale bar is 200 nm.

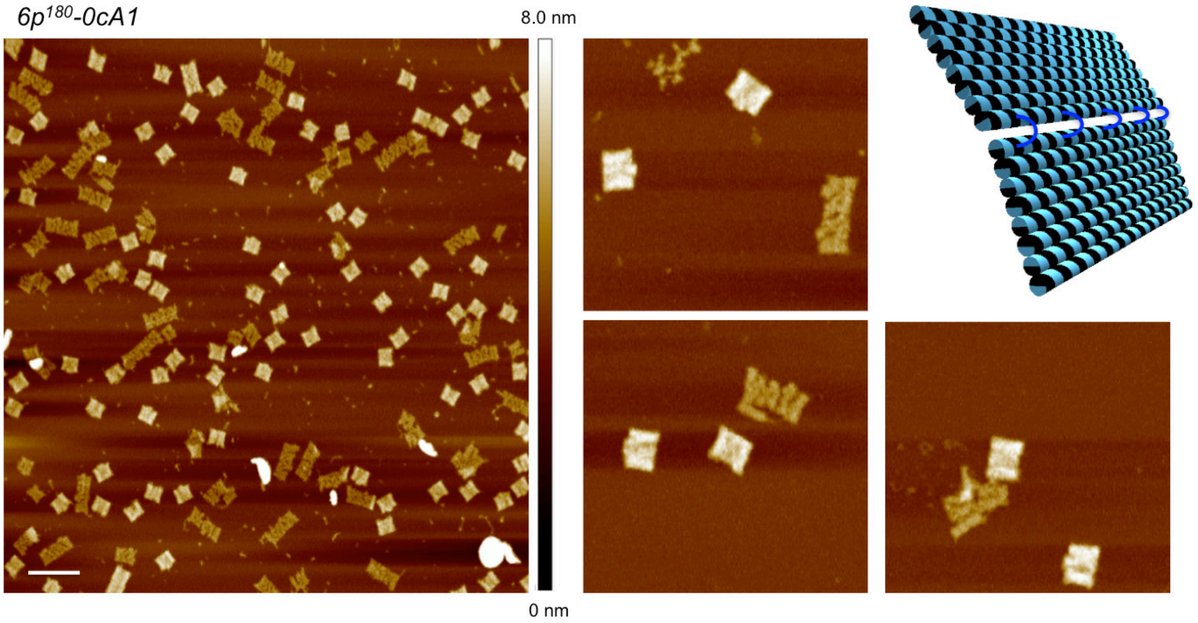

Supplementary Figure 14. AFM imaging of the 6p^{180}-0cA1. In absence of protruding arms, no decoration of the structure is possible. Scale bar is 200 nm.

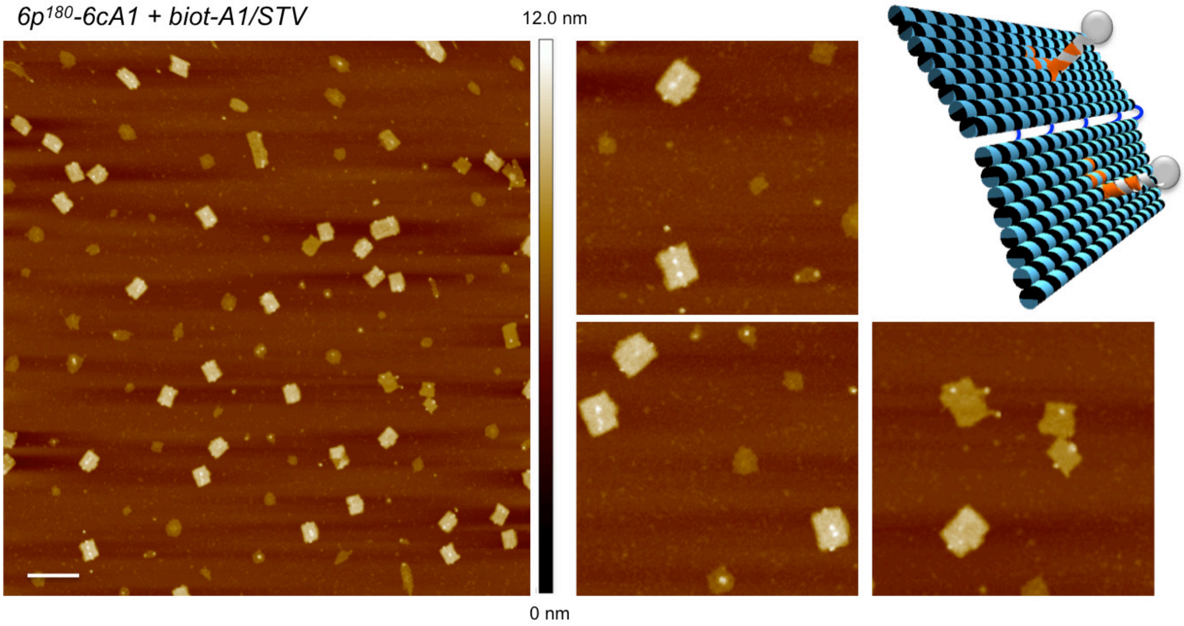

Supplementary Figure 15. AFM imaging of the streptavidin loaded 6p^{180}-6cA1. The structure results from a stochastic mixture of the two possible orientations, with the PAs pointing inwards or outwards. This is evidenced by the appearance of typical rectangular shapes with three brighter spots on top of them and aligned in the center of the structure (associated to PAs pointing outwards). In some cases, instead, encapsulation of streptavidin molecules (associated to PAs pointing inwards) leads to deformation of the structure in its central region with a less evident increase in the height profile. Scale bar is 200 nm.

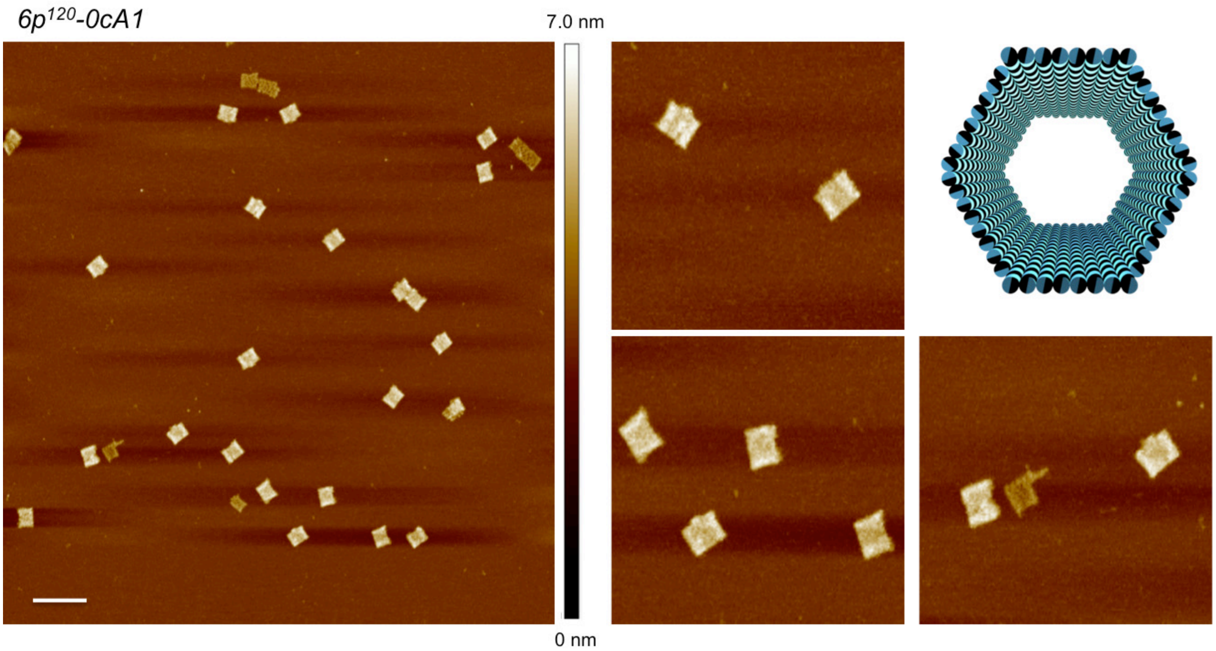

Supplementary Figure 16. AFM imaging of the $6p^{120}$-0cA1. In absence of protruding arms, no decoration of the structure is possible. Scale bar is 200 nm.

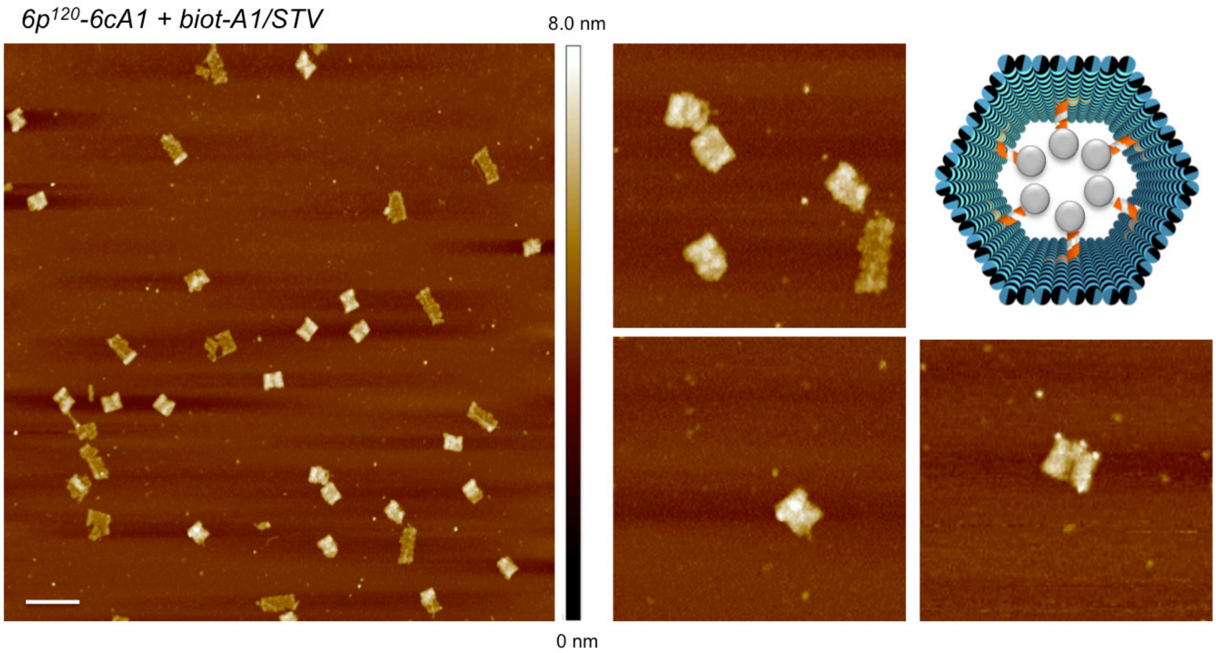

Supplementary Figure 17. AFM imaging of the streptavidin loaded 6p^{120}-6cA1. Correct formation of the structure is evidenced by the appearance of brighter spots in its central region. The poor resolution and broader appearance of such dots indicate increase in the height profile of the structure due to encapsulation of streptavidin molecules. Scale bar is 200 nm.

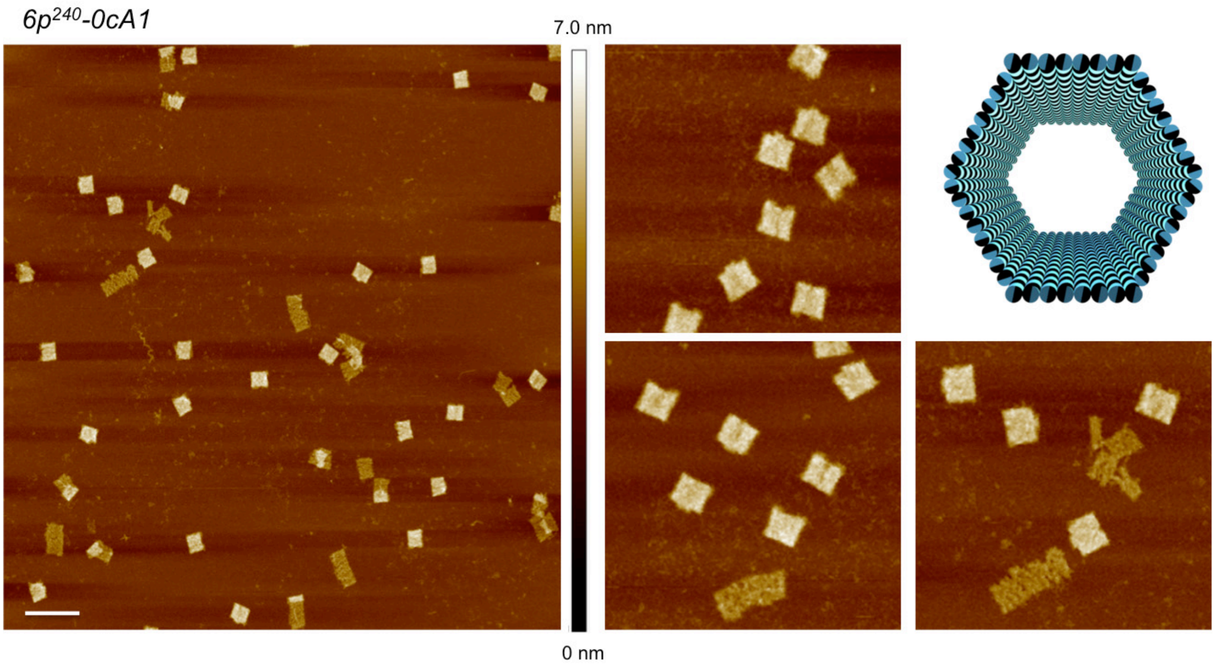

Supplementary Figure 18. AFM imaging of the 6p^{240}-0cA1. In absence of protruding arms, no decoration of the structure is possible. Scale bar is 200 nm.

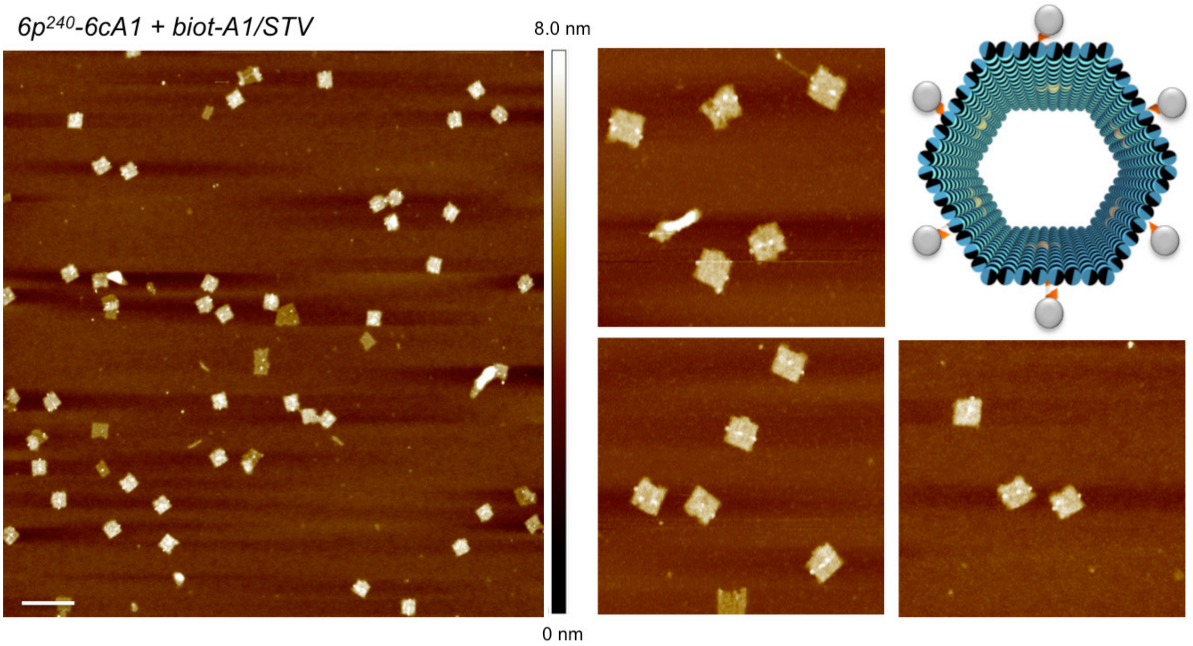

Supplementary Figure 19. AFM imaging of the streptavidin loaded 6p^{240}-6cA1. Correct formation of the structures is evidenced by the clear appearance of three brighter spots aligned in the center of the structure, associated to PAs pointing outwards, which have been modified with streptavidin. Scale bar is 200 nm.

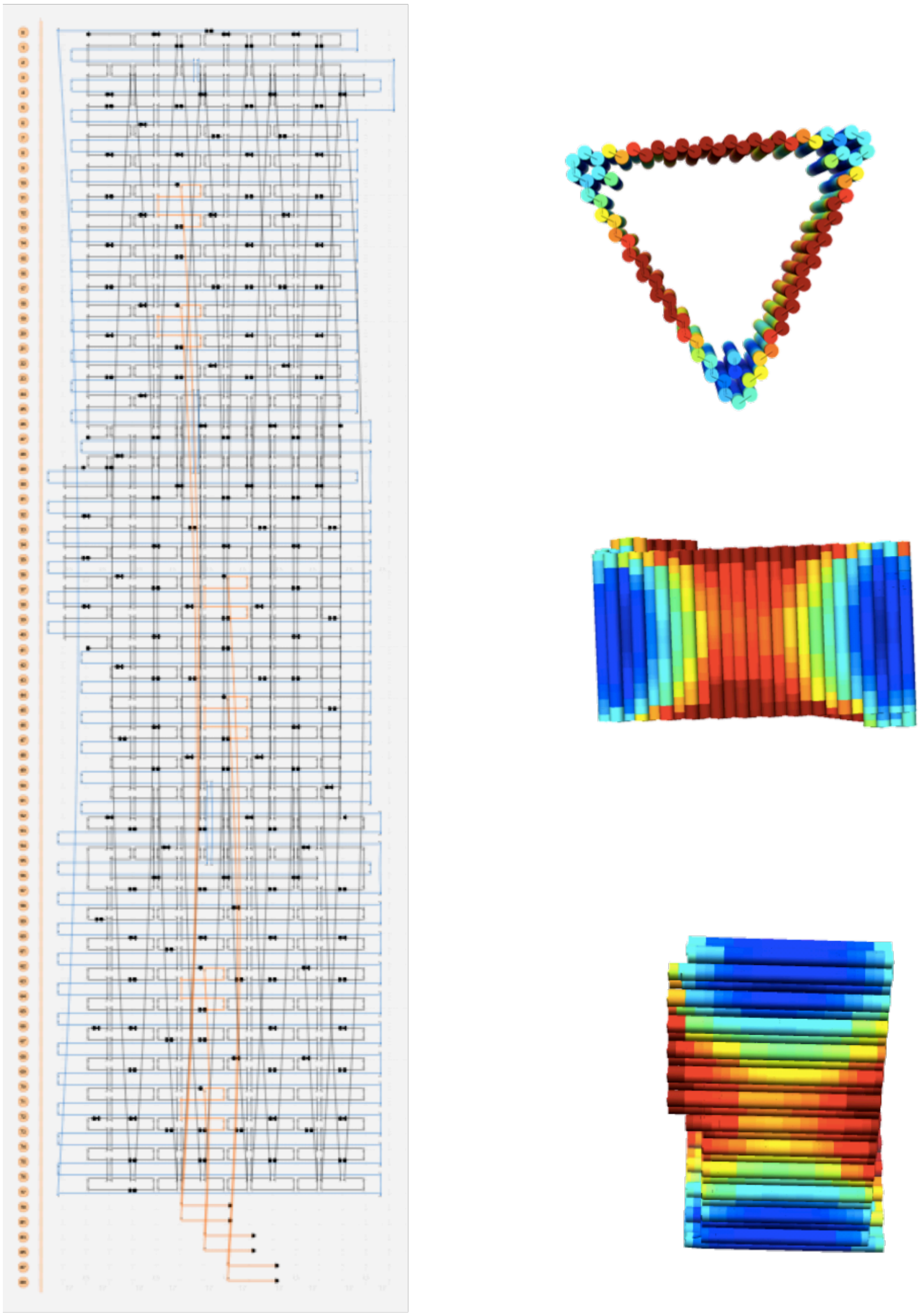

Supplementary Figure 20. Design chart and 3D models of the 3prism construct bearing 6cA1. The models were created by CanDo (http://cando-dna-origami.org).

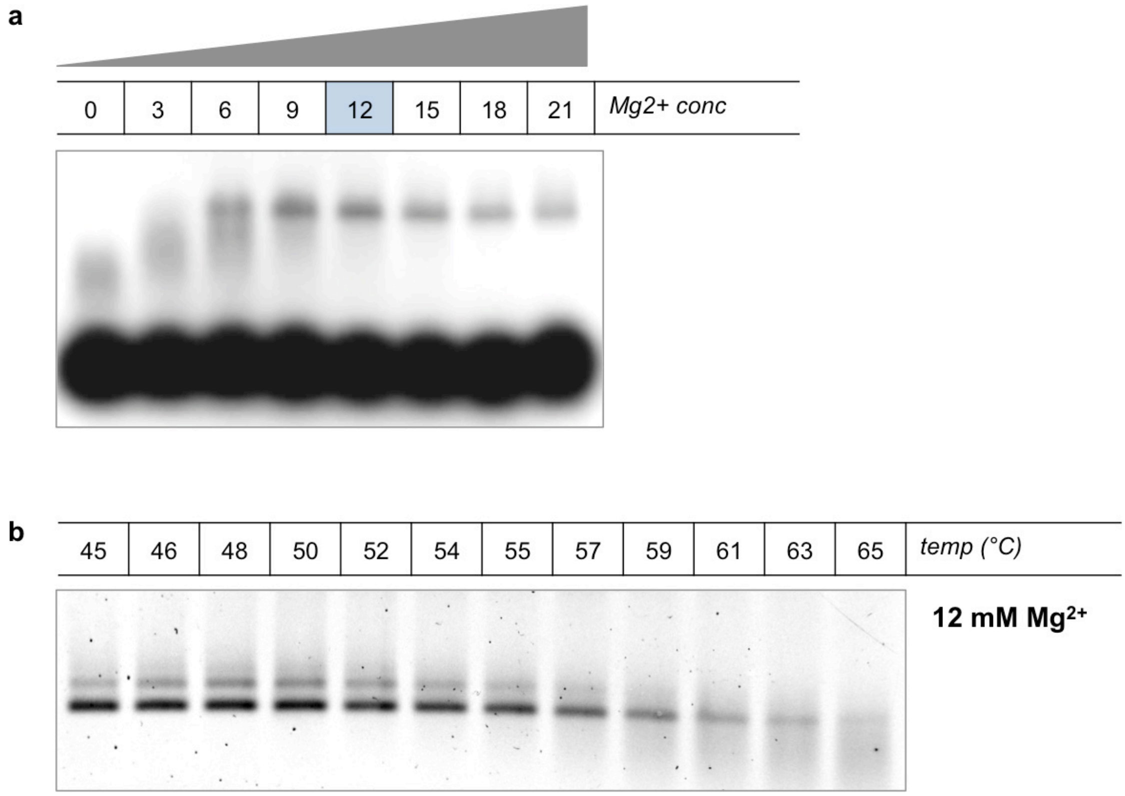

Supplementary Figure 21. Magnesium and temperature screening for the 3prism. Optimal conditions for folding of the 3p construct were found at 12 mM magnesium concentration (**a**). At this concentration, different isothermal annealing assemblies were performed in the range between 45 and 65 °C (**b**). Higher yields were obtained for a long annealing at moderate temperature. Those conditions were then used for assembly of the 3p structure.

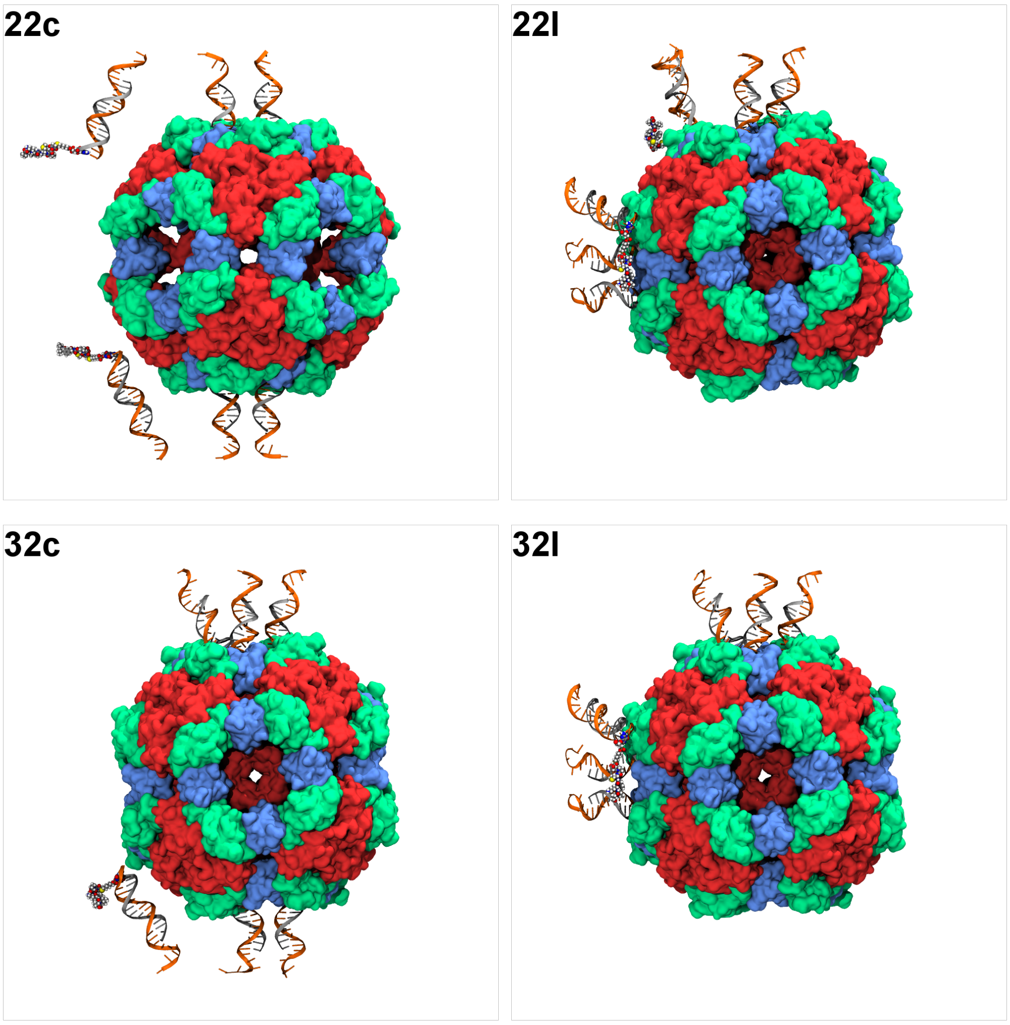

Supplementary Figure 22. MD simulations of DNA-peptide ligands binding to the PDZ1 domains of DegP$_{24}$. The entrances to the cavity of DegP$_{24}$ are big enough to simultaneously host more than one DNA helix. MD simulations were performed to investigate if two or three DNA helices might be hosted simultaneously in one cavity without affecting the binding of the peptide to the PDZ1 domain. Simultaneous binding of two DNA helixes in both entrances (22c and 22l) or three DNA helices to one entrance and two DNA helices to other entrances (32c and 32l) were considered. In addition, the binding to opposite (22c and 32c) or consecutive entrances (22l and 32l) was also modeled. In the figure, the protease domain, the PDZ1 and PDZ2 domains of DegP are shown as red, green and blue surfaces respectively. The DNA strand protruding from the origami is shown in orange and the strand linked to the peptide in grey. The peptide is shown using ball representation.

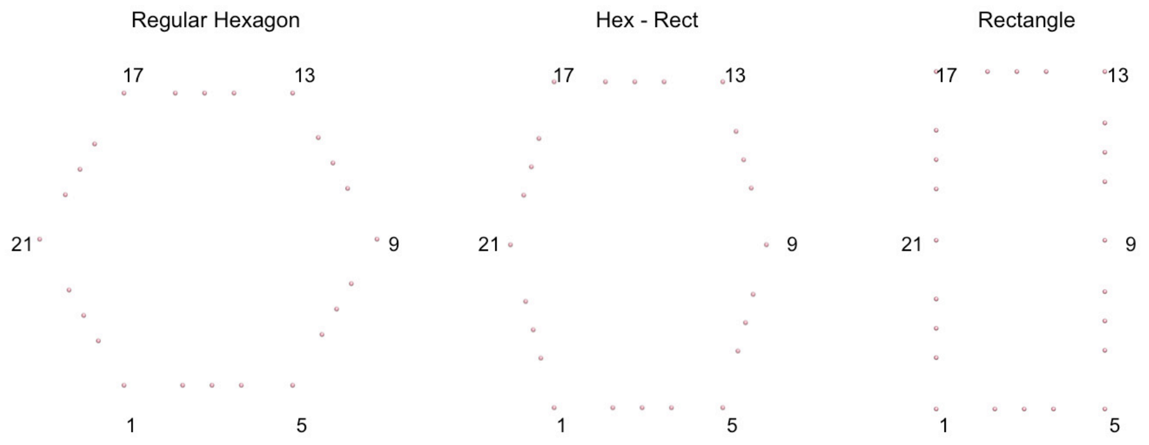

Supplementary Figure 23. The three modes of DNA host configuration. Three geometric models for the origami host were considered: a regular hexagon (h), a distorted hexagon (hr) and a rectangle (r). The origami is depicted by the vertices of the geometric figures describing it and the points where the protruding DNA strands are located. The first point is placed always in the lower left corner and points are numbered counterclockwise. The code for each structure is explained in detail in the Supplementary Methods.

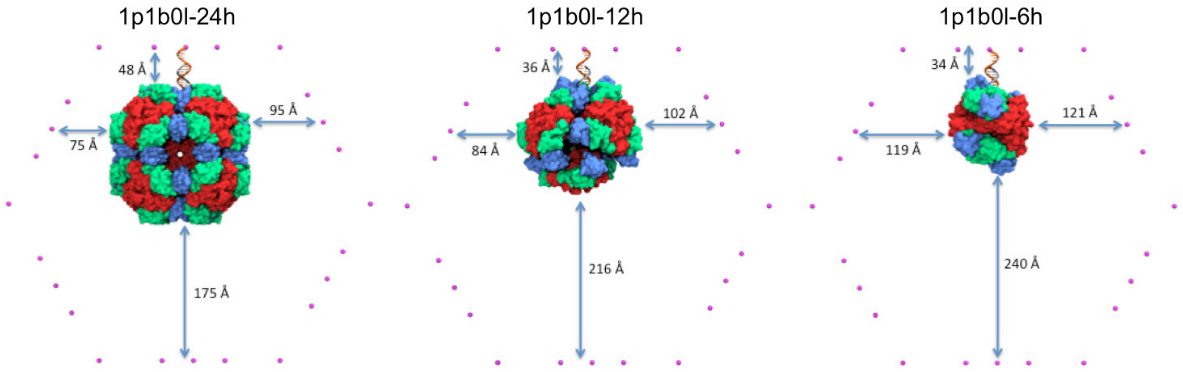

Supplementary Figure 24. Atomistic models for 1p1b0l with h shape.

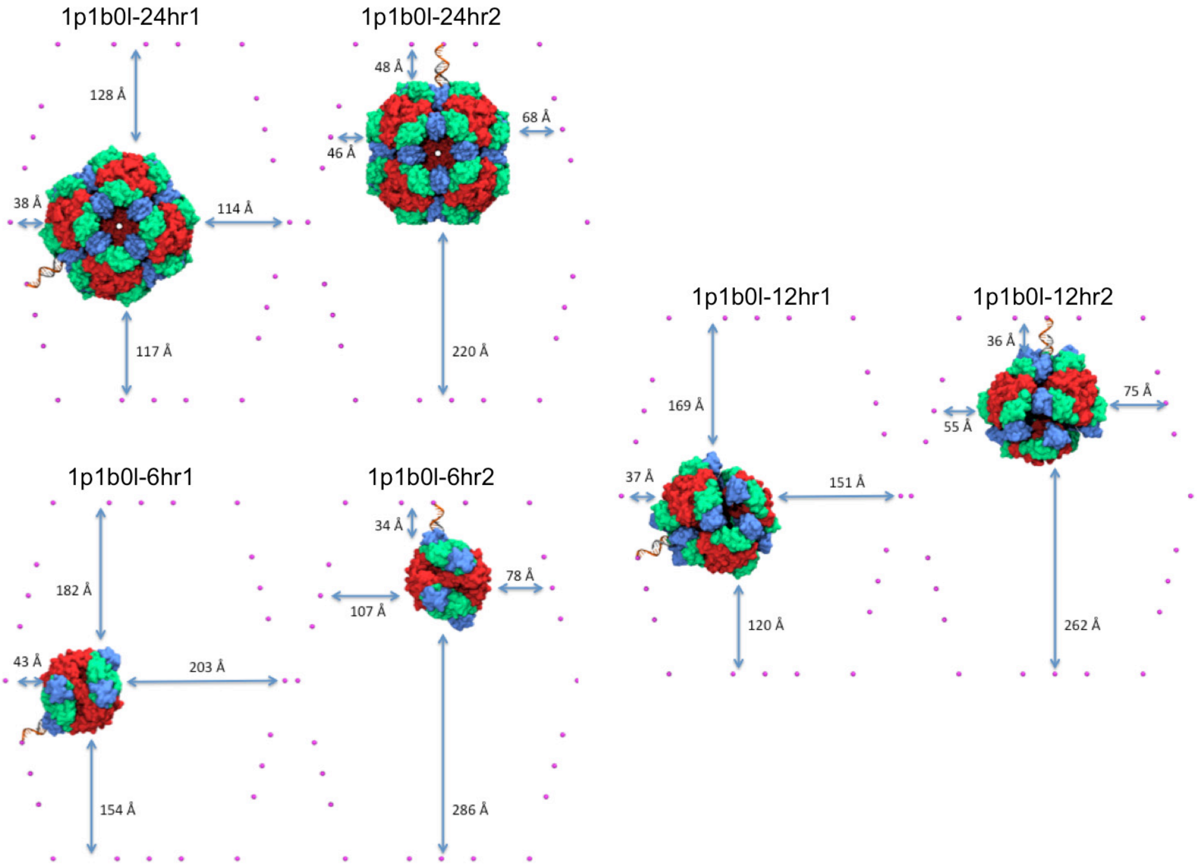

Supplementary Figure 25. Atomistic models for 1p1b0l with hr shape.

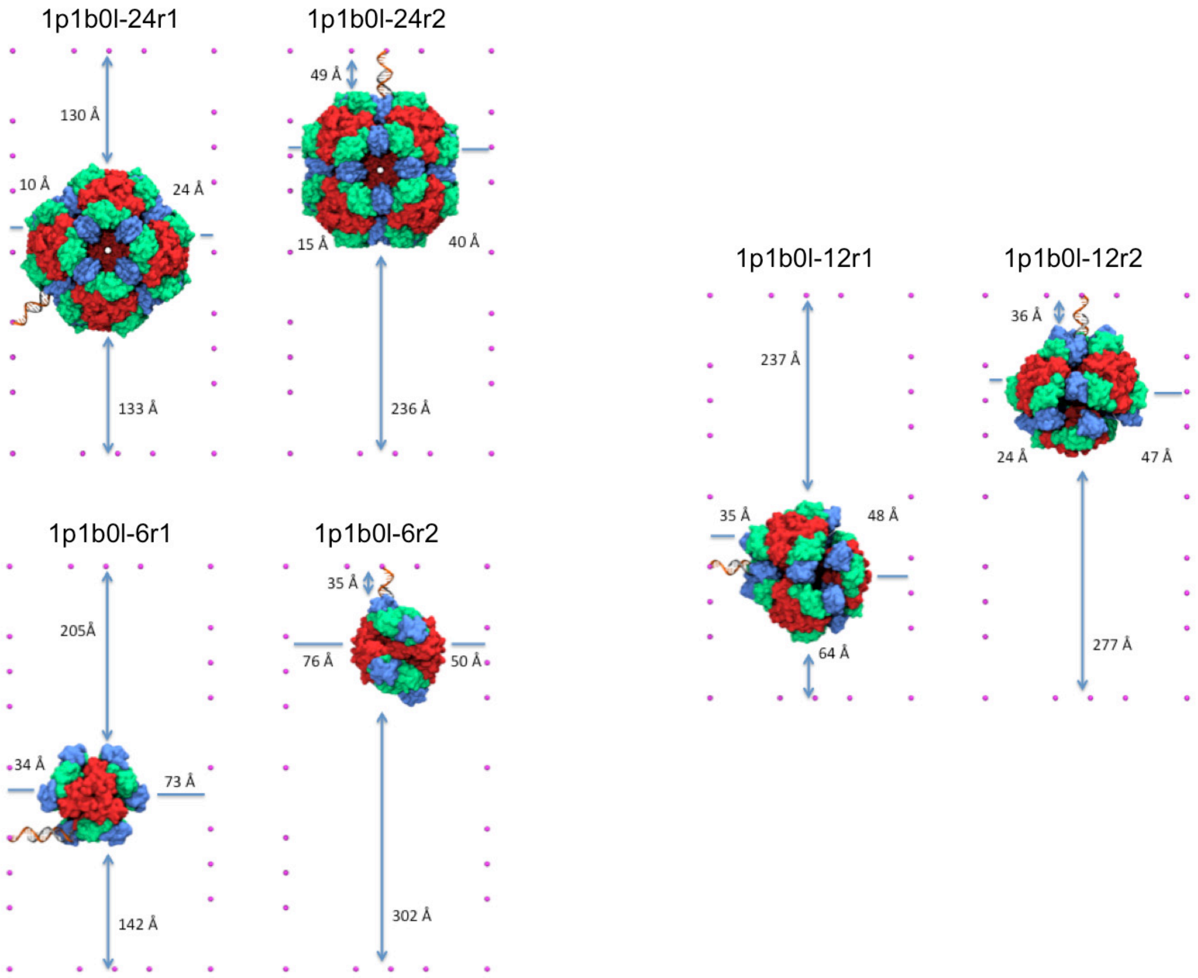

Supplementary Figure 26. Atomistic models for 1p1b0l with r shape.

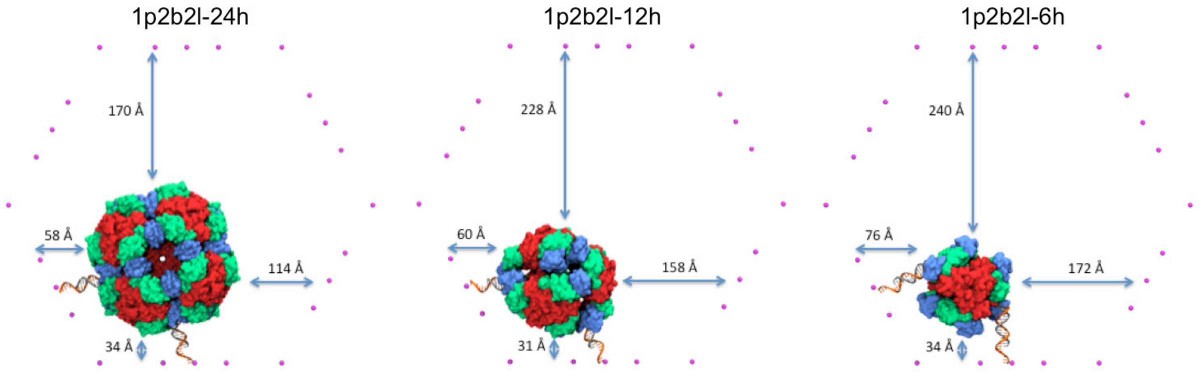

Supplementary Figure 27. Atomistic models for 1p2b2l with h shape.

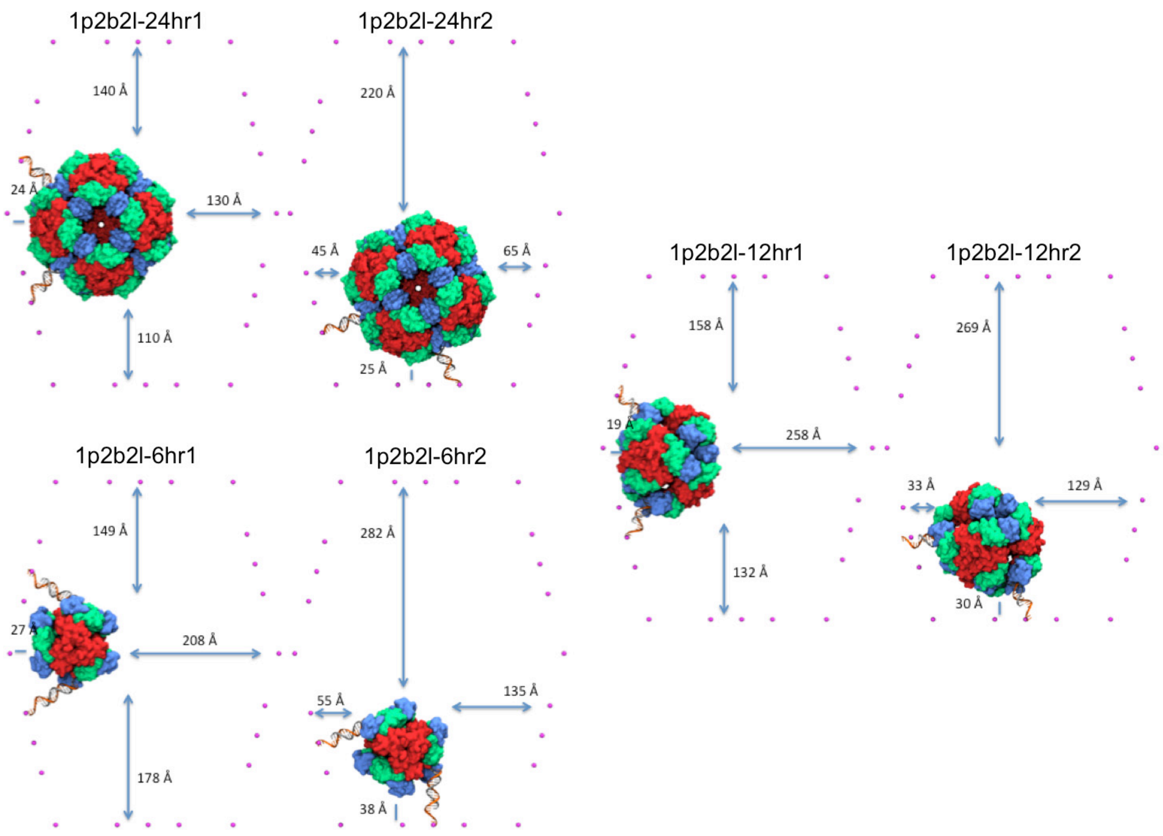

Supplementary Figure 28. Atomistic models for 1p2b2l with hr shape.

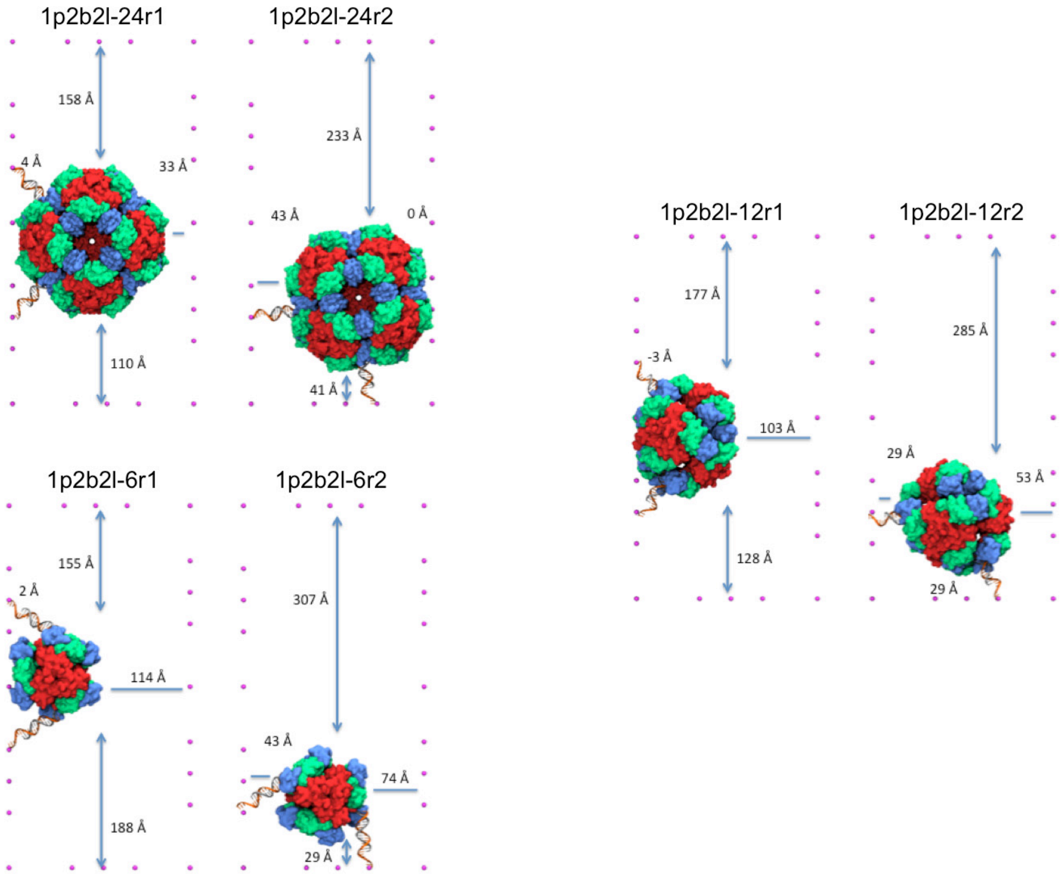

Supplementary Figure 29. Atomistic models for 1p2b2l with r shape.

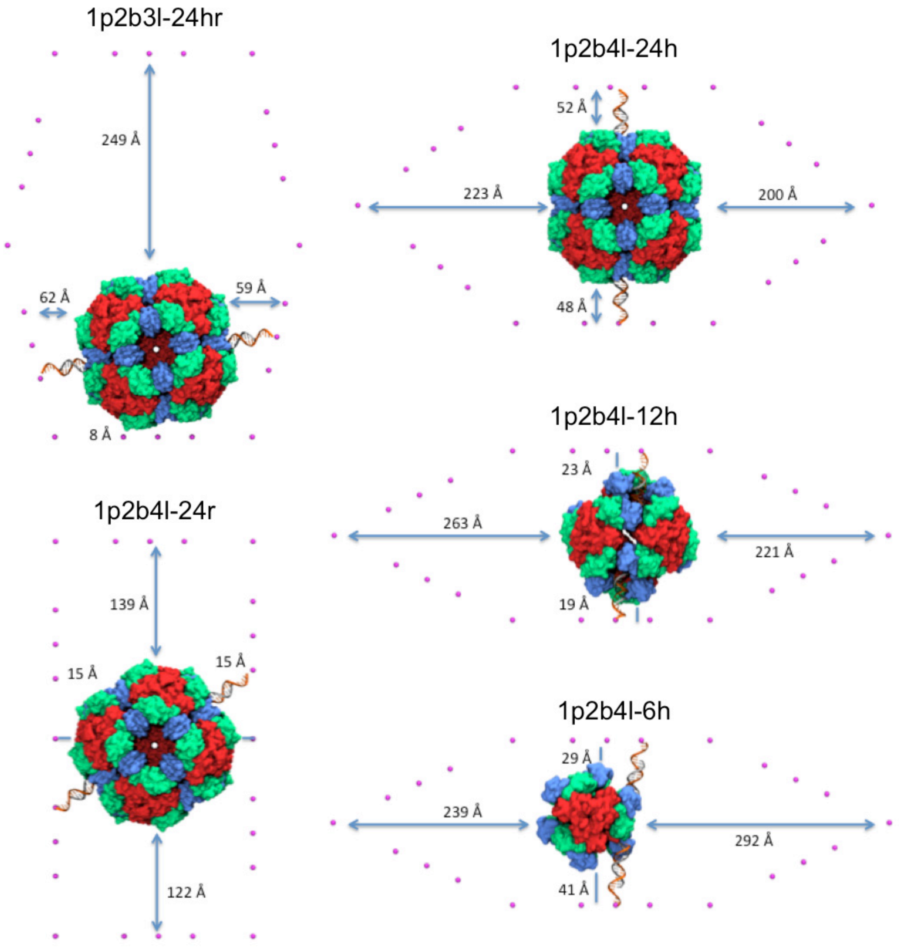

Supplementary Figure 30. Atomistic models for 1p2b with 3l or 4l.

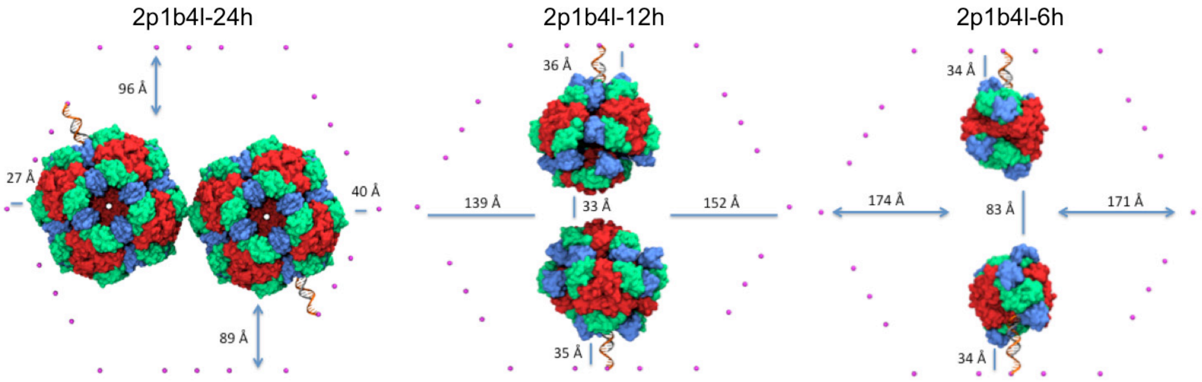

Supplementary Figure 31. Atomistic models for 2p1b4l with h shape.

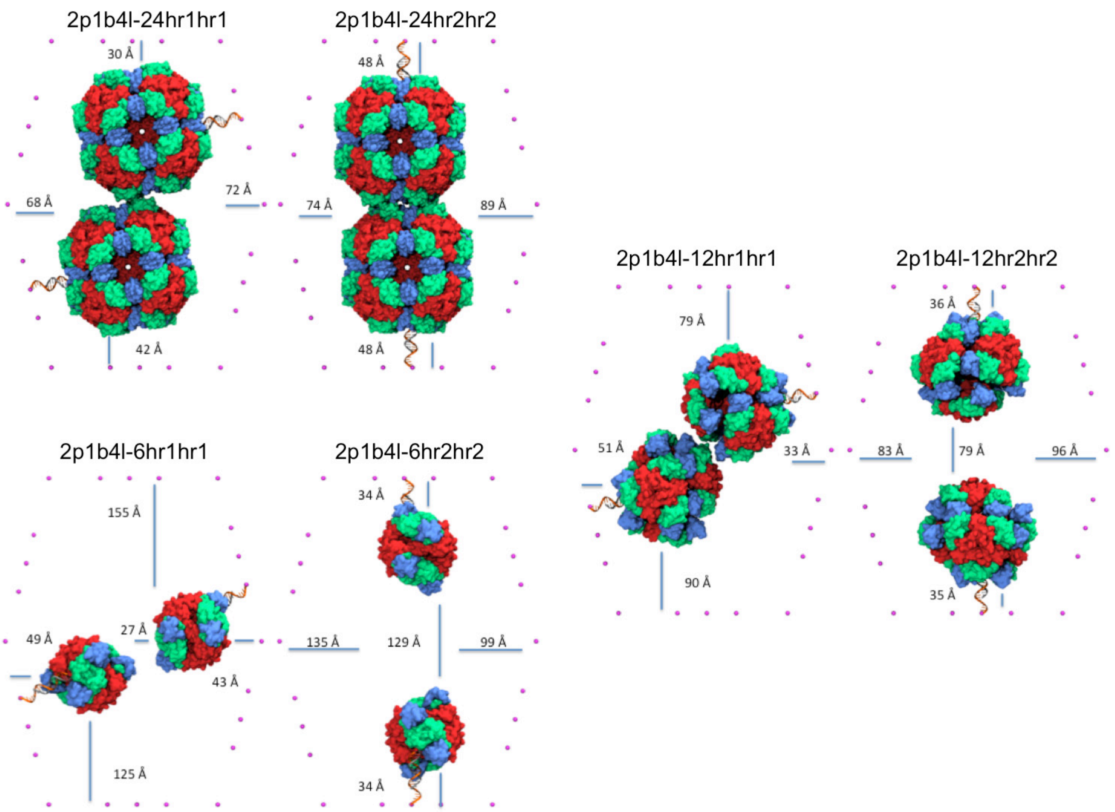

Supplementary Figure 32. Atomistic models for 2p1b4l with hr shape.

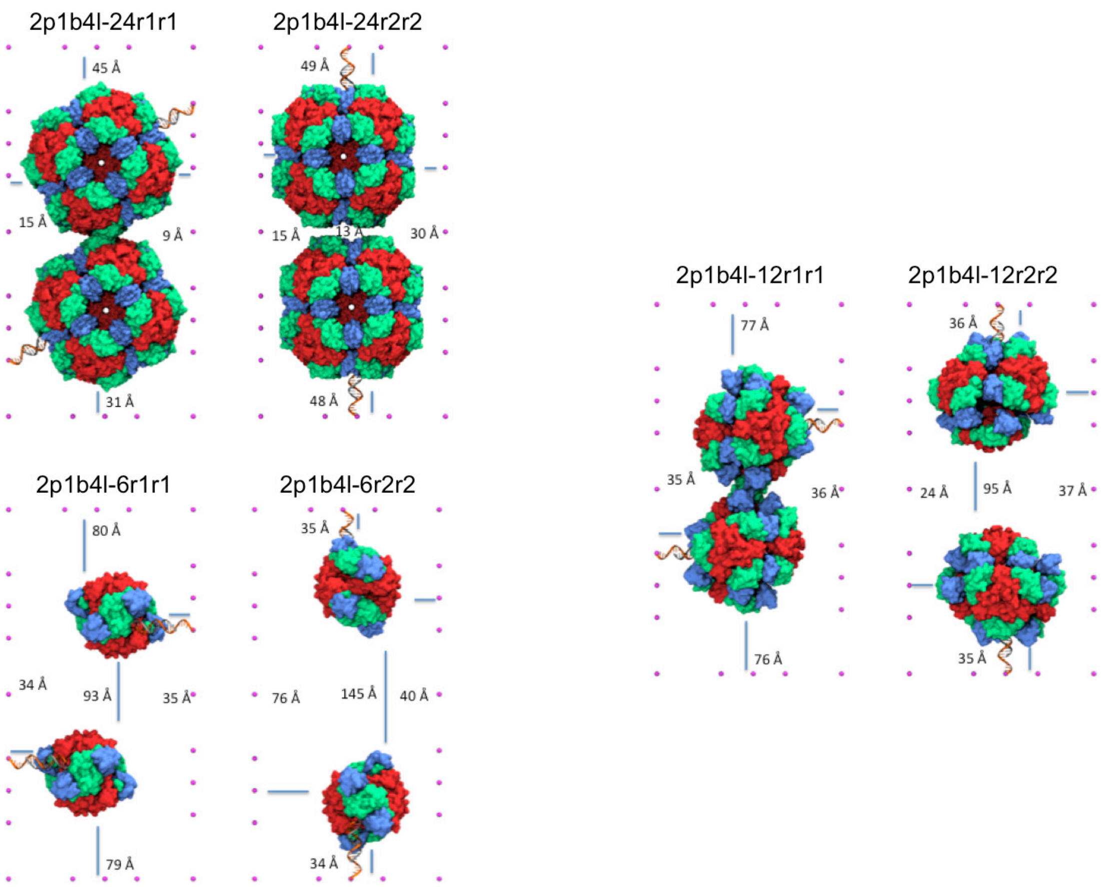

Supplementary Figure 33. Atomistic models for 2p1b4l with r shape.

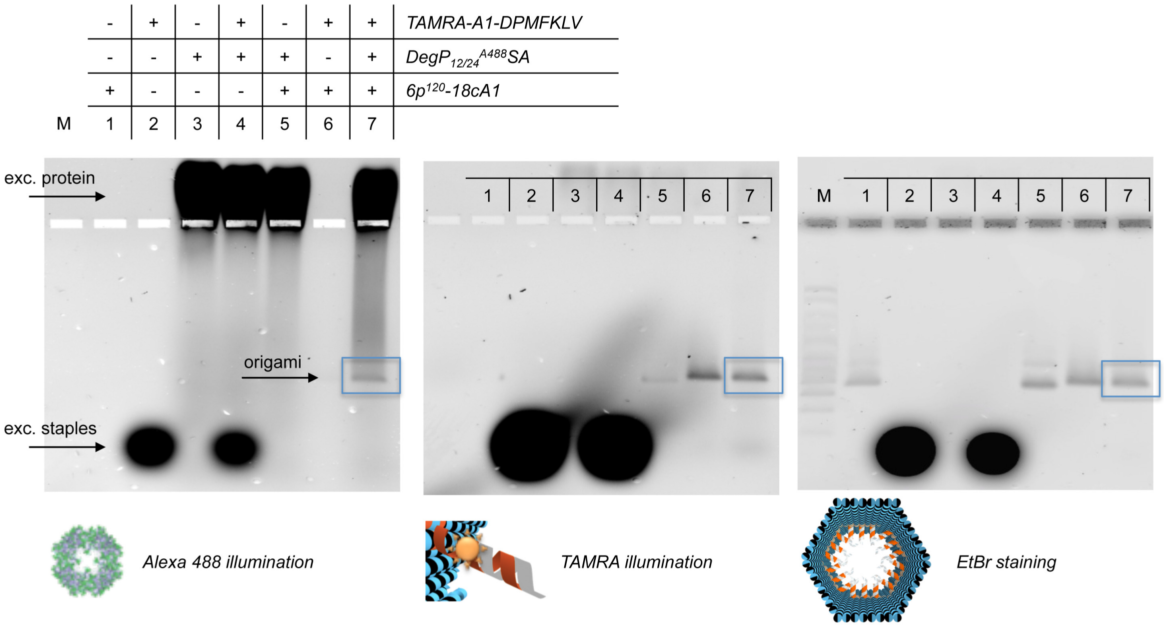

Supplementary Figure 34. Binding of DegP$_{12/24}^{A488}$SA to the 6p host. Only in presence of the peptide ligands (lane 7), the co-migration of the protein, TAMRA-labeled peptides (also visible in lane 6) and DNA origami host occurs, indicating the specificity of binding. The excess of unbound protein migrates towards the negative pole, i.e. opposite to the migration direction of the DNA origami (lanes 3-5 and 7). This suggests a net (but still low) positive charge of the protein; however, *we did not observe any unspecific interactions between the labeled DegP and the negatively charged DNA host*. In fact, as shown in lane 5, no protein signal is present besides the region above the loading pocket and staining with EtBr does not reveal any formation of DNA aggregates. We believe that *the presence of dyes on the protein surface may partially screen the protein positive charge and thus lower unspecific electrostatic interactions with the negatively charged DNA cage*. This in turn might help the recognition-binding event to emerge. This hypothesis has been confirmed by a number of experiments, which aim at analyzing the effect of protein surface modification on its loading efficiency (Supplementary Figs. 35-43). Gel conditions: 0.75% agarose in 1X TBEMg, at 80V for 2:15h at 4°C. The gel was scanned with a Typhoon FLA 9000 at different wavelengths to record the presence of DegP protein (A488-labeled) and peptide ligand (TAMRA-labeled). DNA was visualized upon staining with ethidium bromide. Lane M contained a 1 kbp DNA ladder. The DNA origami (lane 1) migrates between the 1.5 and 2.0 kbp bands of the ladder.

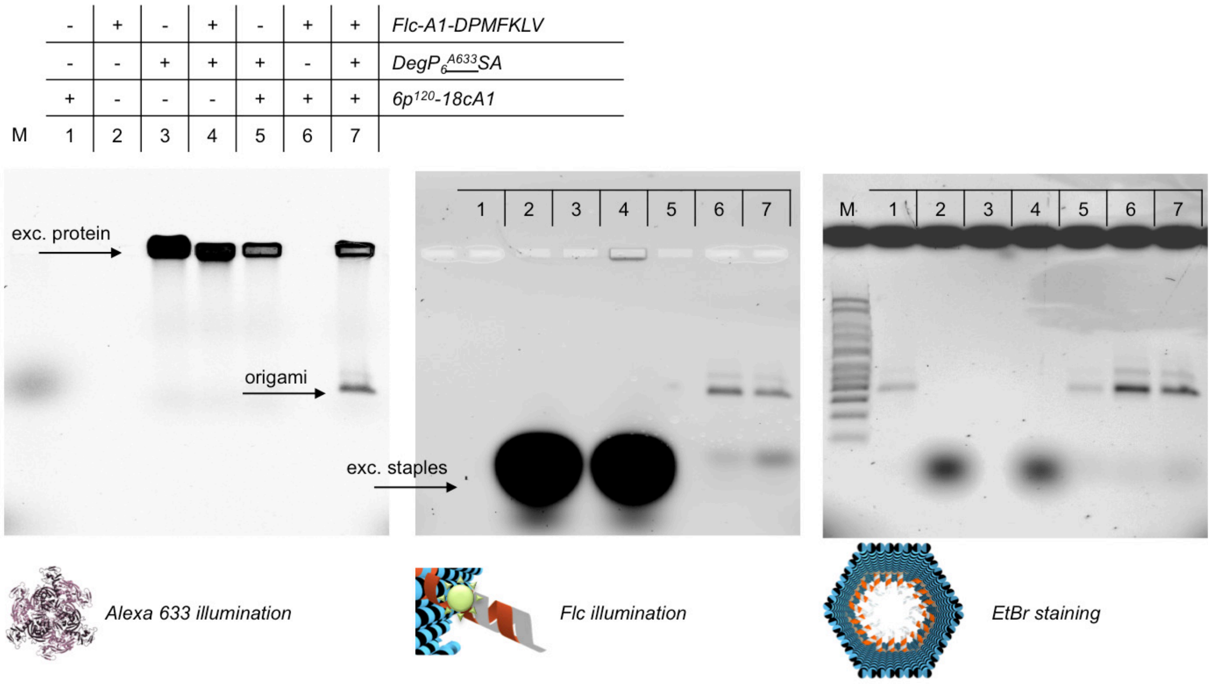

Supplementary Figure 35. Binding of $DegP_6^{A633}$SA to the 6p host. The same conclusions drawn for the gel shown in Supplementary Fig. 34 are valid here. i.e. only in presence of the peptide ligands (lane 7), specific binding occurs. This gel therefore shows that the *encapsulation of DegP works well for distinct protein oligomerization states* (cfr. the 12/24-mer of Supplementary Fig. 34 with the 6-mer here), *as well as different dyes and labeling chemistries*. Indeed, whereas in the $DegP_{12/24}^{A488}$SA the dyes are attached to the lysine residues, in the $DegP_6^{A633}$SA the dyes are attached instead at genetically introduced cysteine residues. At this point, one should note that the huge difference in the total number of Lys or Cys residues/monomeric protein unit results of course in a drastic difference in the labeling grade of the two proteins. In fact, whereas the 12/24-mer has about 26 lysine per unit, resulting in a total of more than 600 possible addressable sites per protein, the 6-mer displays only 3 cysteine residues per unit leading to a total of about 18 attachment points for binding of the dyes. The higher labeling grade of the 12/24-mer when compared to the 6-mer would lead to a higher depletion of the positive surface charge that could also partially explain its higher binding yield, as shown by statistical AFM analysis of the purified products (Figure 4f of the main manuscript). Lane M contained a 1 kbp DNA ladder. The DNA origami band (lane 1) migrates between the 1.5 and 2.0 kbp bands of the ladder. Gel composition and running conditions as in Supplementary Fig. 34.

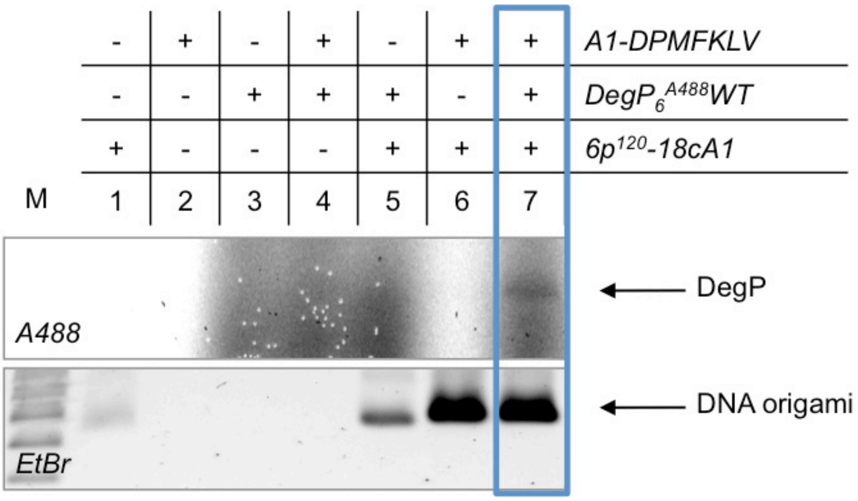

Supplementary Figure 36. Binding of DegP$_6^{A488}$WT to the 6p host. Loading appears to be successful and specific also for the wild-type (WT) protein form, thus confirming the general applicability of the method. That is, *the S210A mutation, as well as all other genetic modifications applied in this work, do not affect the binding capability of the DegP protein to the functionalized DNA host*. Lane M contained a 1 kbp DNA ladder. The DNA origami band (lane 1) migrates between the 1.5 and 2.0 kbp bands of the ladder. Gel composition and running conditions as in Supplementary Fig. 34.

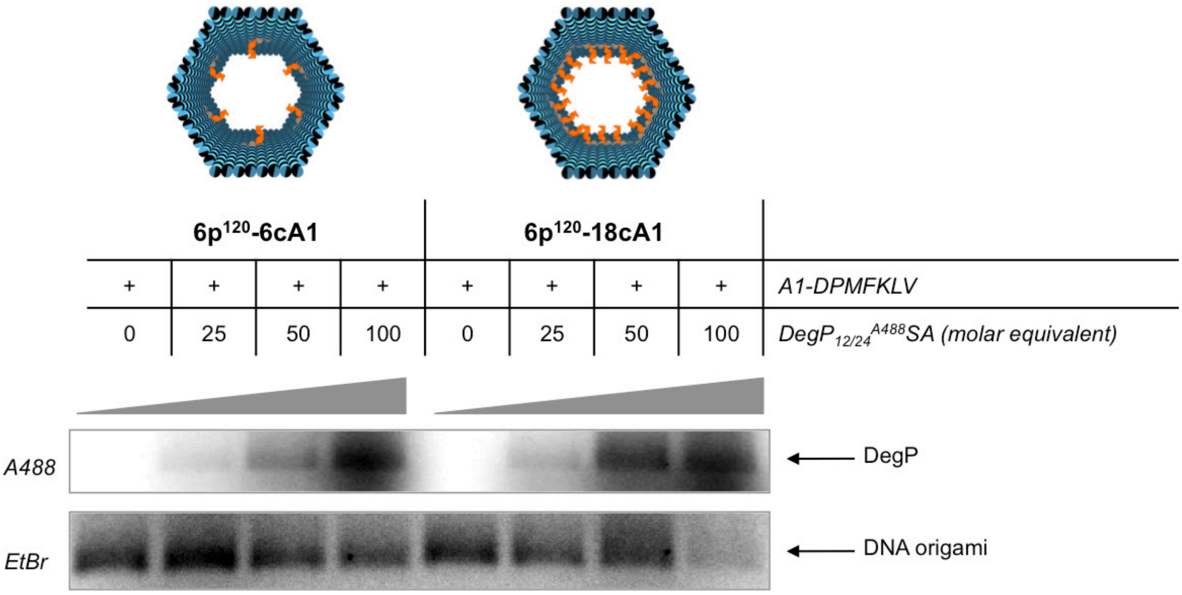

Supplementary Figure 37. Binding of DegP$_{12/24}$^{A488}SA to the 6p host at different stoichiometric ratios. The efficiency of protein loading into the nanochannel has been investigated as a function of protein concentration (from 0 to 100 molar equivalent) and number of protein ligands (either 6 or 18). One can notice that in presence of an equal molar excess of protein, the higher number of ligands in the 6p^{120}-18cA1 leads to a higher binding efficiency, with ca. 36% yield in presence of 25 molar equivalent of protein and almost complete saturation (82%) at 50-fold excess of protein. On the contrary, using the 6-arms structure, saturation is reached at 100 molar equivalents of DegP protein. These results indicate that the *efficiency of protein binding indeed increases with the number of ligands, which are available in the vicinity of the protein surface*. Gel composition and running conditions as in Supplementary Fig. 34.

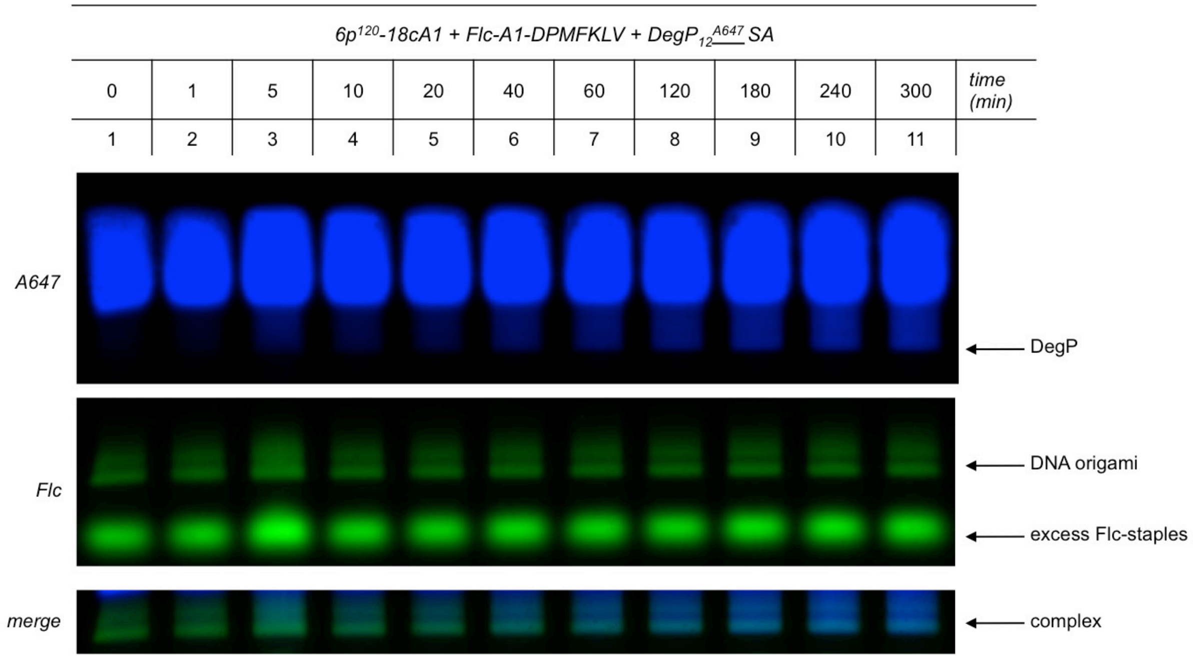

Supplementary Figure 38. Time-course binding of $DegP_{12}^{A647}$SA to the 6p host. Maximal binding efficiency is reached already after ca. 180 min (lane 9), which can turn to be useful when working with unstable proteins. Once again, comparing these results with the gels shown in Supplementary Figs. 34 and 35 (respectively, binding of $DegP_{12/24}^{A488}$SA and $DegP_{6}^{A633}$SA to $6p^{120}$-18cA1) one can conclude that *specific binding occurs independently of the protein oligomerization state, type of fluorescent dye used and labeling chemistry*. Gel composition and running conditions as in Supplementary Fig. 34.

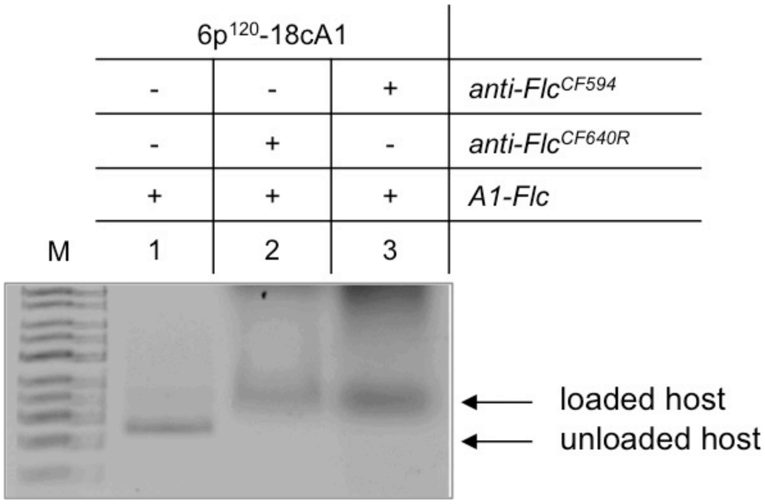

Supplementary Figure 39. Binding of anti-fluorescein antibodies to the 6p host. Contrarily to DegP, binding of the anti-fluorescein antibodies to the Flc-modified DNA origami host leads (as expected) to a remarkable retardation in the electrophoretic mobility of the complex (cfr. lanes 2 and 3 with lane 1). Although more experiments would be necessary to elucidate this issue, these preliminary studies suggest that the *multivalency of ligand binding and the surface charge of the DegP protein both contribute to the yield of protein encapsulation and to the electrophoretic mobility properties of the DNA-DegP complex* (more details on these issues are provided in the Supplementary Discussion). Lane M contained a 1 kbp DNA ladder. The unloaded DNA origami (lane 1) migrates between the 1.5 and 2.0 kbp bands of the ladder. Gel composition and running conditions as in Supplementary Fig. 34.

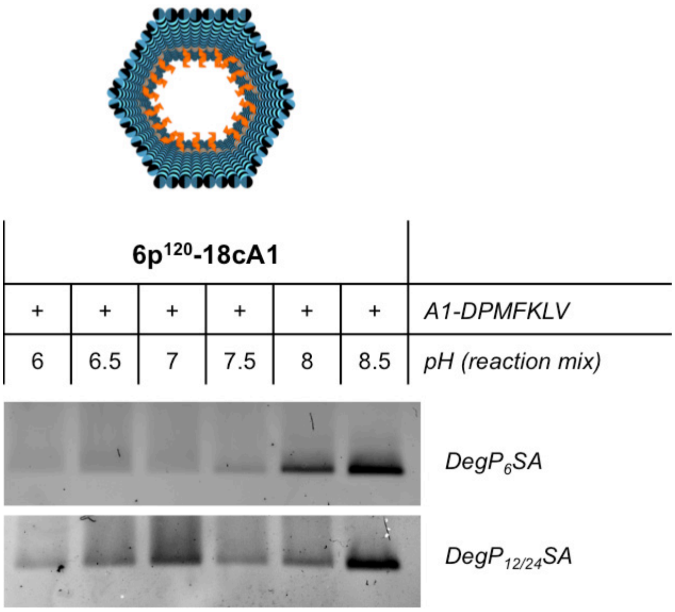

Supplementary Figure 40. Binding of $DegP_6SA$ and $DegP_{12/24}SA$ to the 6p host at different pH values. For both proteins, increasing the pH from 6 to 8.5 results in the appearance of a better-defined band of higher intensity. This suggests that the interaction between the protein and the DNA host is improved when the net charge of the protein is slightly lowered (less positive): we suppose that increasing the pH, the lysine residues exposed on the protein surface will be mostly deprotonated, thus reducing the unspecific electrostatic interactions with the negatively charged DNA surface and allowing for the specificity of the peptide-PDZ1 interaction to emerge. Unspecific binding at lower pH is also evident by formation of a less compact band and a gel smear. Please note that in this experiment, the protein is not labeled, therefore it cannot be visualized by a fluorescence signal. The DNA origami structure is also subject to a migration rate variation at different pH conditions. Nevertheless, the different behavior observed between the two sample sets (i.e. the 6prism in presence of $DegP_6$ or $DegP_{12/24}$) allows us to compare the results obtained at different pH values. Gel composition and running conditions as in Supplementary Fig. 34.

.

6p^120-18cA1									
pH 7.6			pH 6.0		pH 7.6		pH 6.0		
-	-	-	-	-	+	+	+	+	*molecular tweezers*
-	+	+	+	+	+	+	+	+	*DegP$_{12}$SA*
-	+	-	+	-	+	-	+	-	*TAMRA-A1*
+	-	+	-	+	-	+	-	+	*TAMRA-A1-DPMFKLV*
1	2	3	4	5	6	7	8	9	

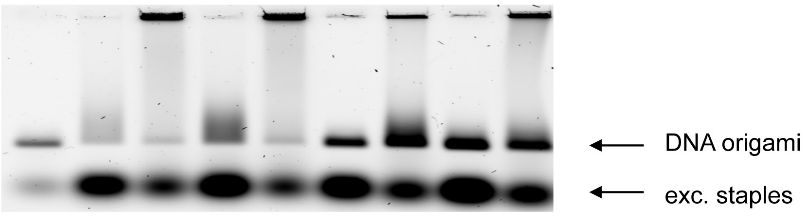

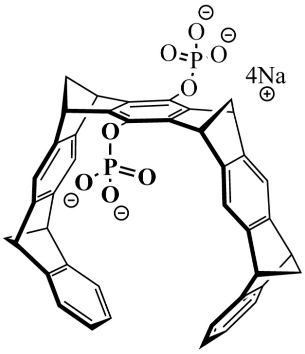

Supplementary Figure 41. Effect of lysine-selective molecular tweezers on DegP binding. In this binding experiments we investigated the effect of three parameters on formation of the DNA-DegP complex: i.e. the presence or absence of the peptide ligand, the pH of the reaction mixture and finally, the presence or absence of Lys-selective molecular tweezers. The DNA host loaded with A1-peptide ligands leads to a well-defined band (lane 1). Addition of the protein leads to formation of a well-defined band of weaker intensity and similar migration rate (lanes 3 and 5). Lack of peptide ligands (lanes 2 and 4) leads instead to an undefined mixture of products of different migration properties, as evidenced by the appearance of long gel smears. This indicates that *in absence of protein ligands, unspecific electrostatic interactions between the positively charged protein surface and the negatively charged DNA host dominate*. As expected, such an effect is more marked at lower pH (cfr. lane 2 with lane 4). Addition of Lys-selective molecular tweezers (lanes 6 to 9) leads in all cases to formation of better-defined bands of higher intensity and almost complete disappearance of gel smears. This suggests a drastic reduction of unspecific electrostatic interactions between the protein and the DNA host due to charge screening of the exposed ε-amino groups of the lysine residues. The action of the tweezers is not markedly affected by pH change (cfr. lanes 6 and 7 with lanes 8 and 9). The lack of labels on the protein surface and the similarity of the gel migration properties between the loaded and the unloaded host would in principle not allow to prove here the specific binding of the protein in presence of the peptide. Nevertheless, one can clearly notice that the unbound protein and the excess of peptide ligands in solution result in formation of large oligomeric forms of DegP visible as bands in the gel pockets (cfr. lanes 3 and 5 with 7 and 9). These are much more intense in absence (lanes 3 and 5) rather than in presence (lanes 7 and 9) of tweezers, thus supporting the hypothesis of tweezers-induced protein internalization. Gel composition and running conditions as in Supplementary Fig. 34.

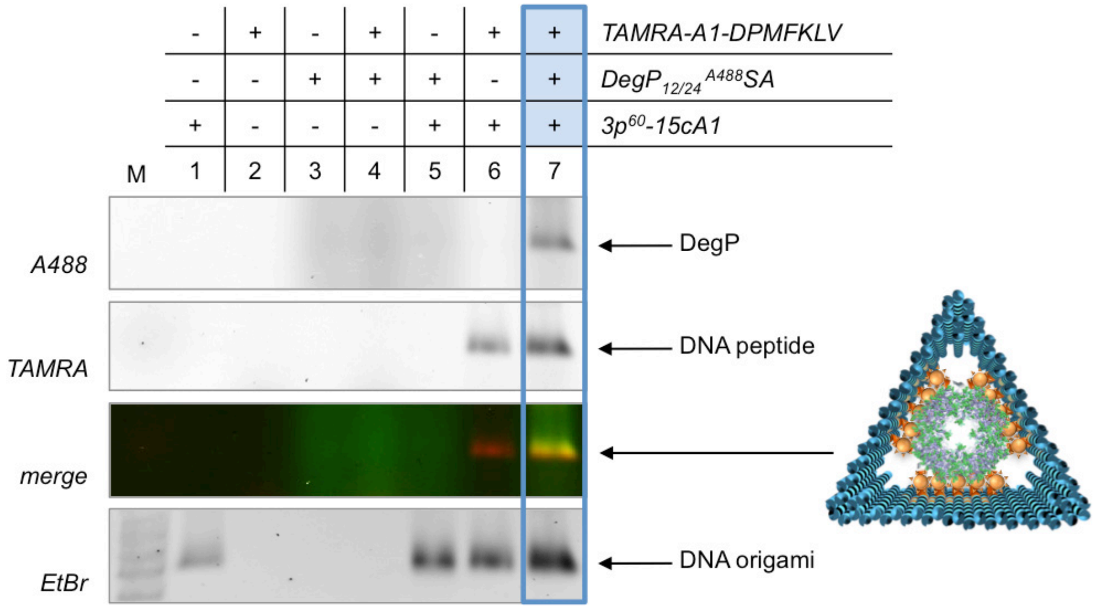

Supplementary Figure 42. Binding of DegP$_{12/24}$^{A488}SA to the 3p host. Also in this case, specific binding occurs only in presence of the peptide ligands (lane 7). Unexpectedly, the protein binds to the 3prism DNA origami cage despite its much smaller inner room when compared to the 6p construct. These results suggest that our DNA cages are not structurally rigid but may be partially deformed, thus allowing accommodation of large proteins inside the inner cavity. This was indeed shown by AFM characterization of the empty cages, although, as confirmed by DLS, such a structural flexibility does not hamper full integrity and folding of the structures in solution (Supplementary Fig. 53). Lane M contained a 1 kbp DNA ladder. The DNA origami (lane 1) migrates between the 1.5 and 2.0 kbp bands of the ladder. Gel composition and running conditions as in Supplementary Fig. 34.

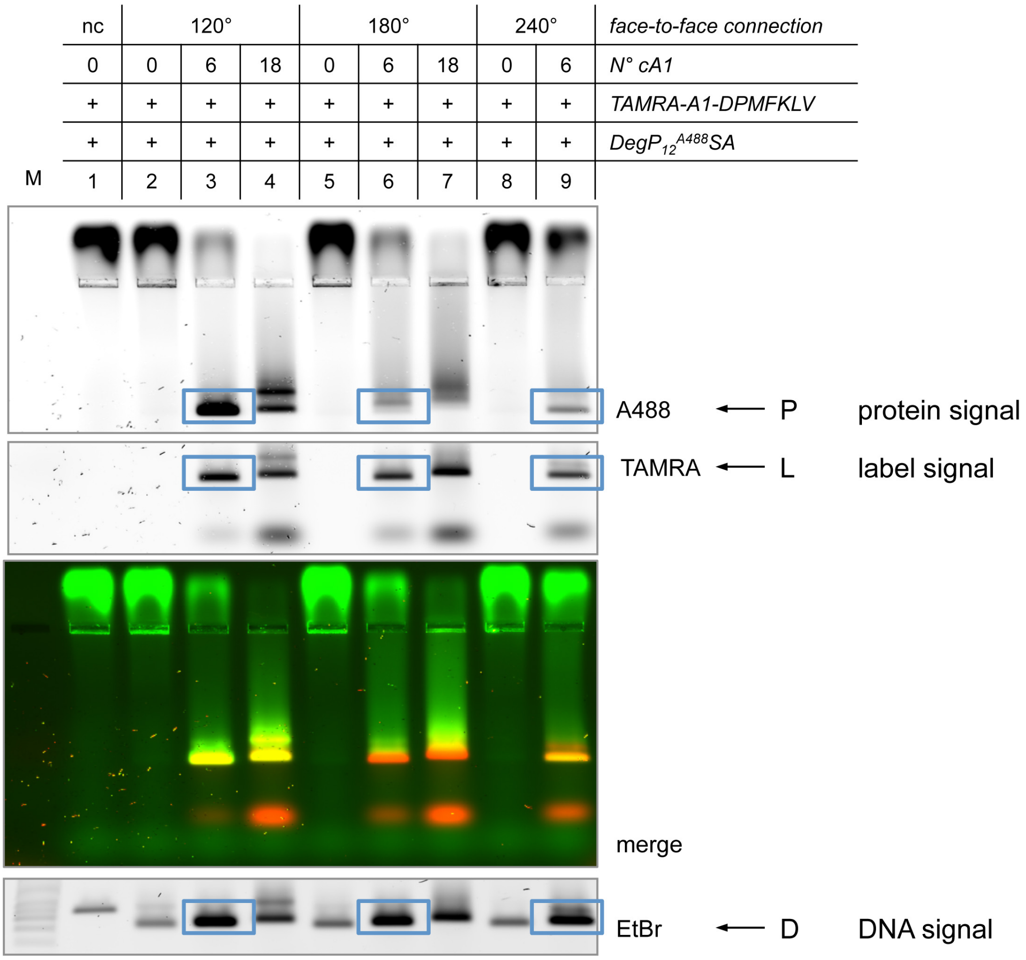

Supplementary Figure 43. Full view of the gel given in Figure 2b of the main manuscript. For a full description, see legend to Figure 2b of the main manuscript. Lane M contained a 1 kbp DNA ladder. The DNA origami (lane 1) migrates between the 1.5 and 2.0 kbp bands of the ladder. The procedure adopted for calculation of the yield by gel electrophoresis is reported in the Supplementary Methods.

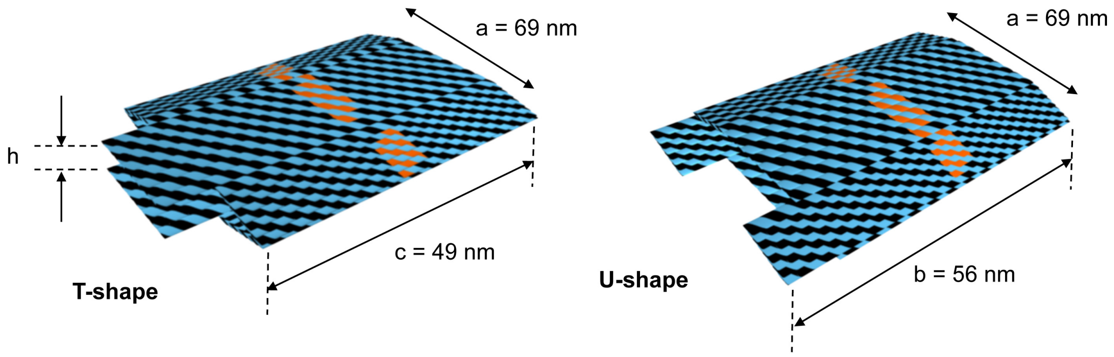

	a	*b*	*c*	*h*
theor. values (nm)	69	56	49	4
exp. values (nm)	71 ± 5	58 ± 4	52 ± 4	3.1 ± 0.4

Supplementary Figure 44. Analysis of the unloaded 6p construct under AFM imaging in air. Deformation of the structure under AFM imaging leads to two possible flat shapes, depending on the axis along which compression is acting: a T-shape (35%) and a U-shape (65%). As expected, 2/3 of possible collapsed structures should assume a U-shape because 2 out of 3 axes may lead to this configuration. Total structures = 703. The dimensions of the observed structures well match with the theoretical values derived from flattening of a correctly formed shape.

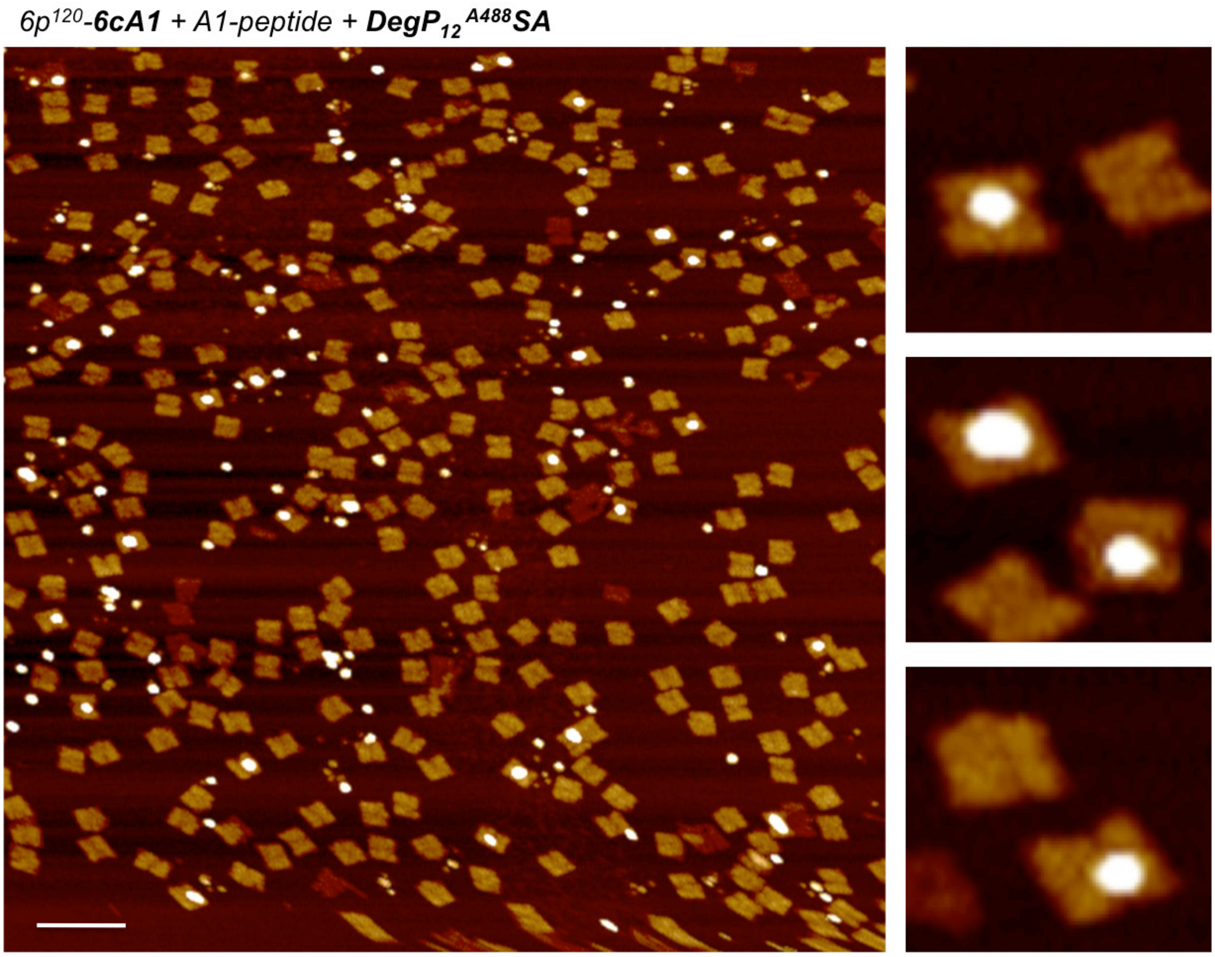

Supplementary Figure 45. AFM imaging of DegP$_{12}^{A488}$SA binding to 6p^{120}-6cA1. Scale bar is 200 nm.

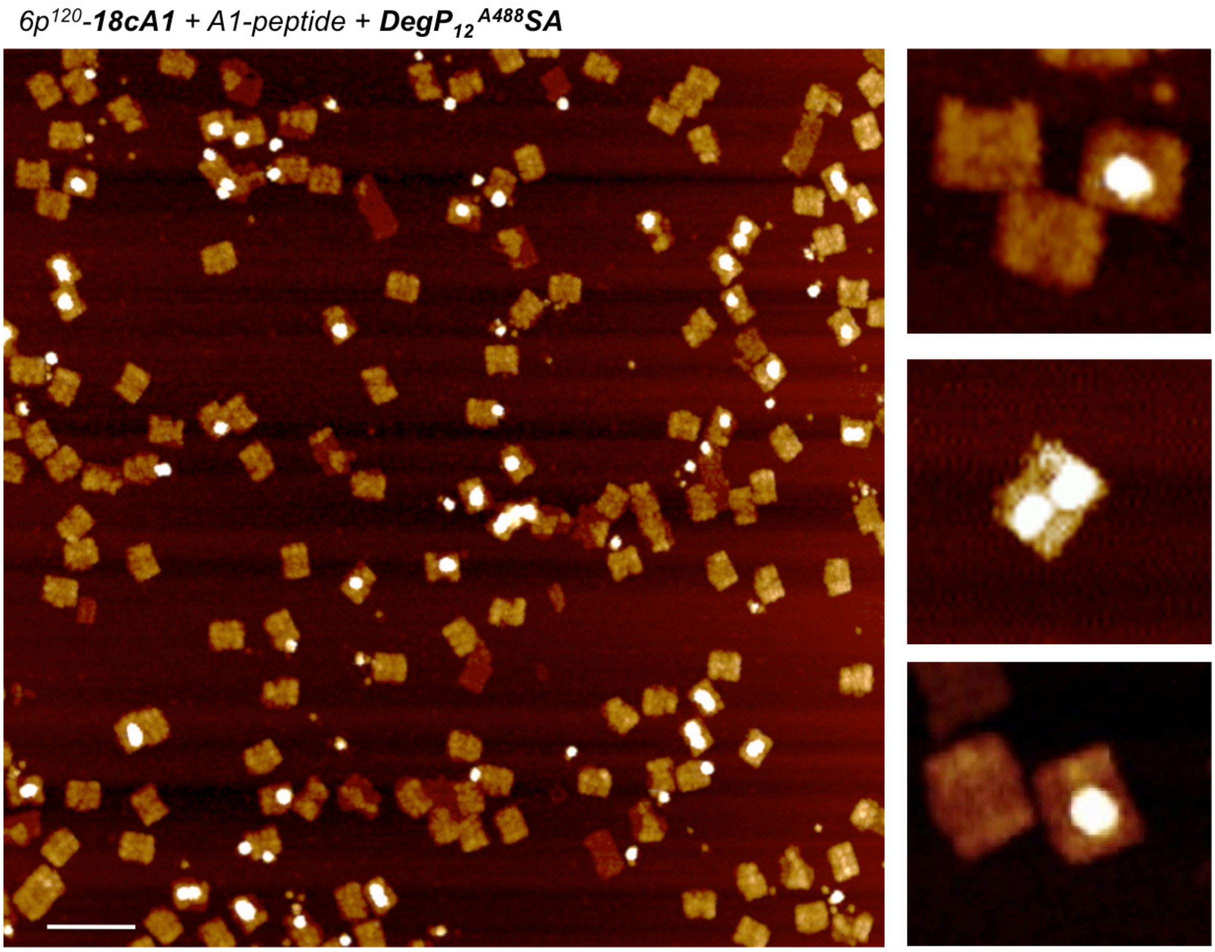

Supplementary Figure 46. AFM imaging of DegP$_{12}$^{A488}SA binding to 6p^{120}-18cA1. Scale bar is 200 nm.

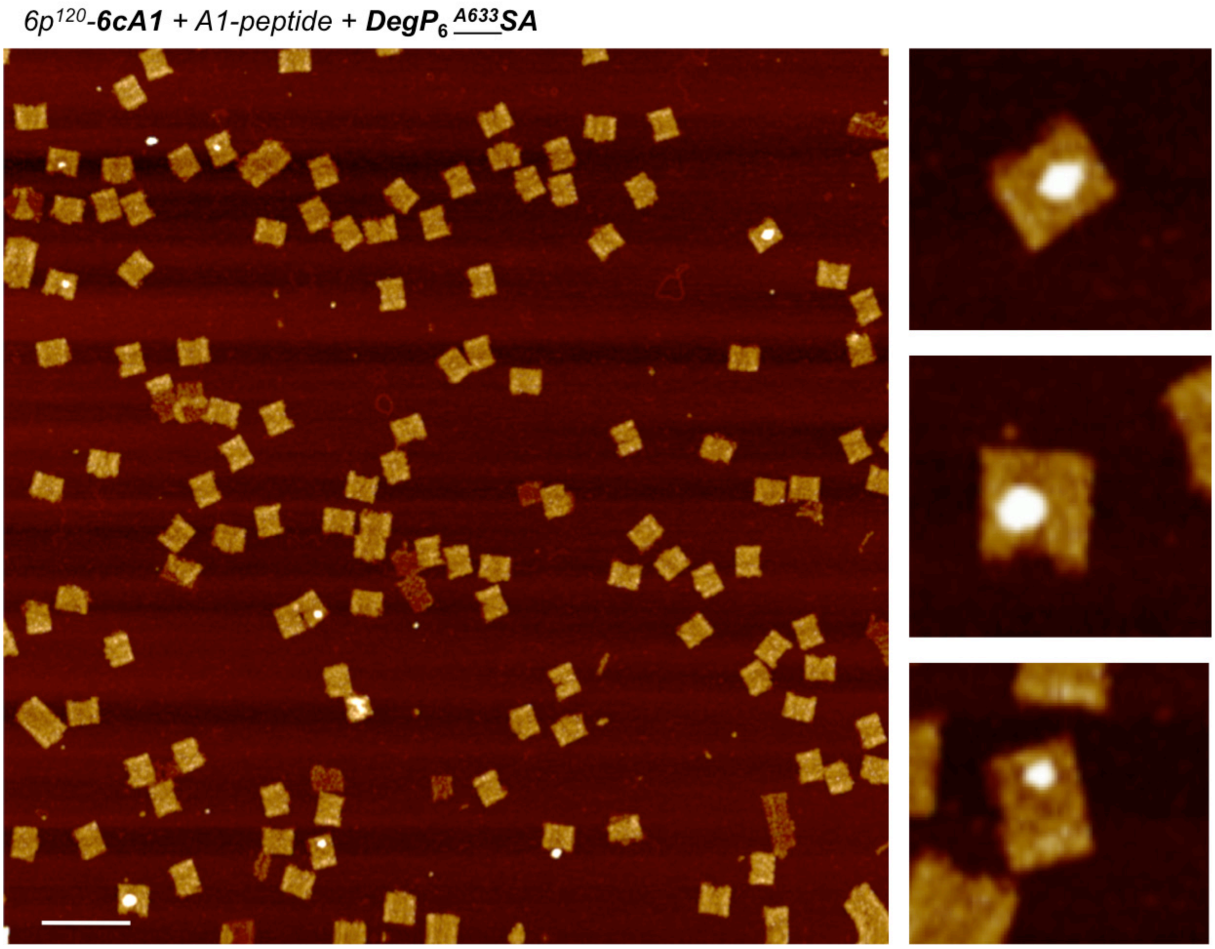

Supplementary Figure 47. AFM imaging of DegP$_6^{\underline{A633}}$SA binding to 6p^{120}-6cA1. Scale bar is 200 nm.

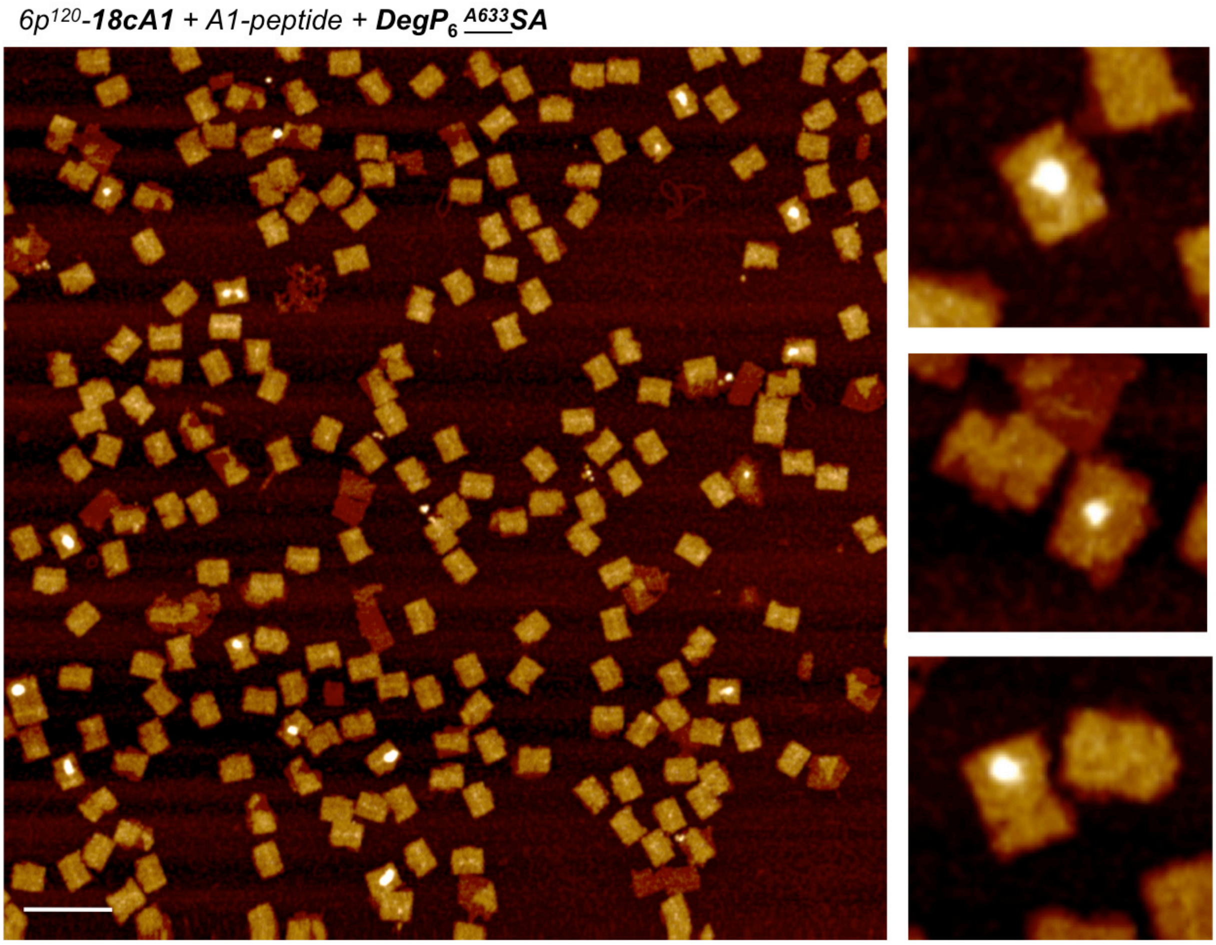

Supplementary Figure 48. AFM imaging of DegP$_6$$\underline{^{A633}}$SA binding to 6p^{120}-18cA1. Scale bar is 200 nm.

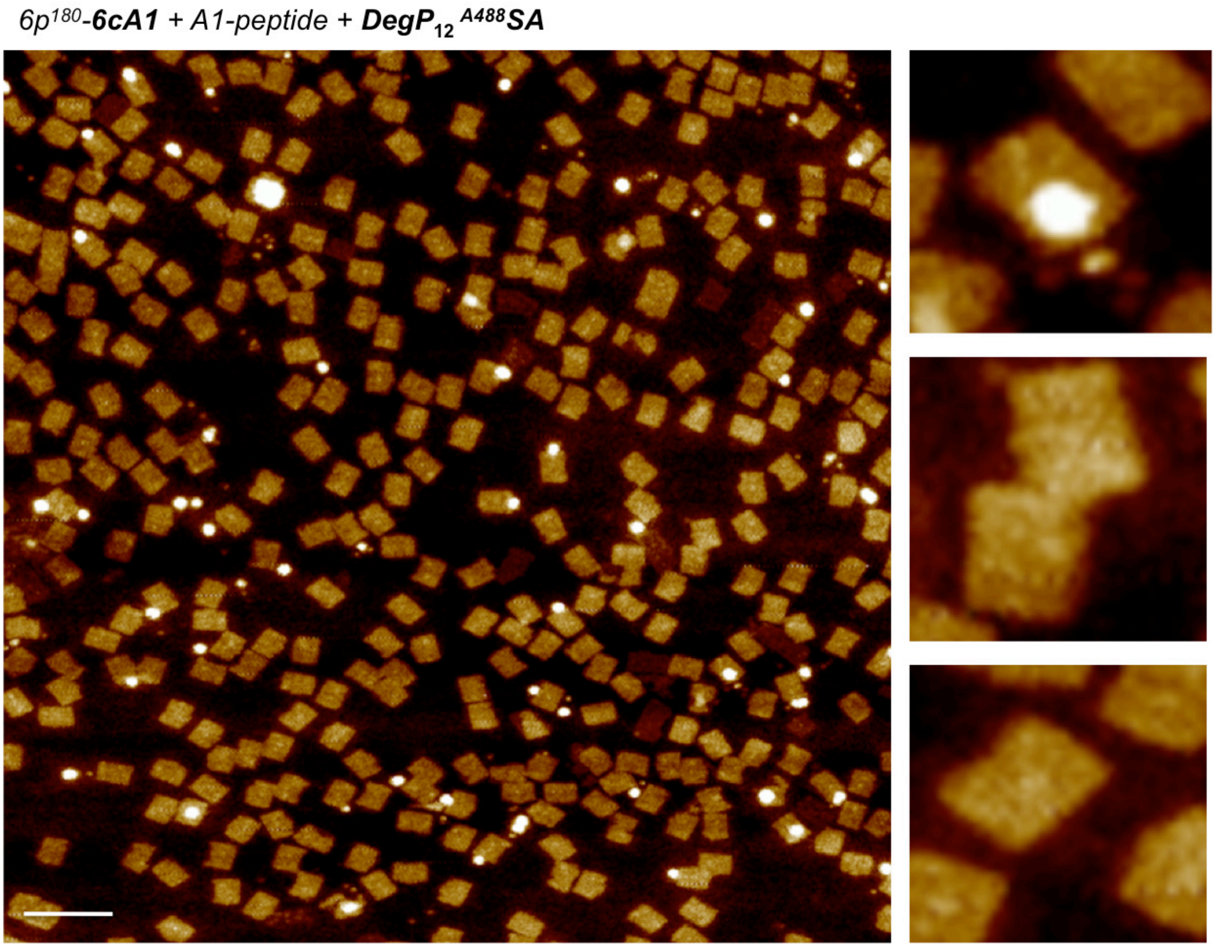

Supplementary Figure 49. AFM imaging of $DegP_{12}^{A488}SA$ binding to $6p^{180}$-6cA1. Scale bar is 200 nm.

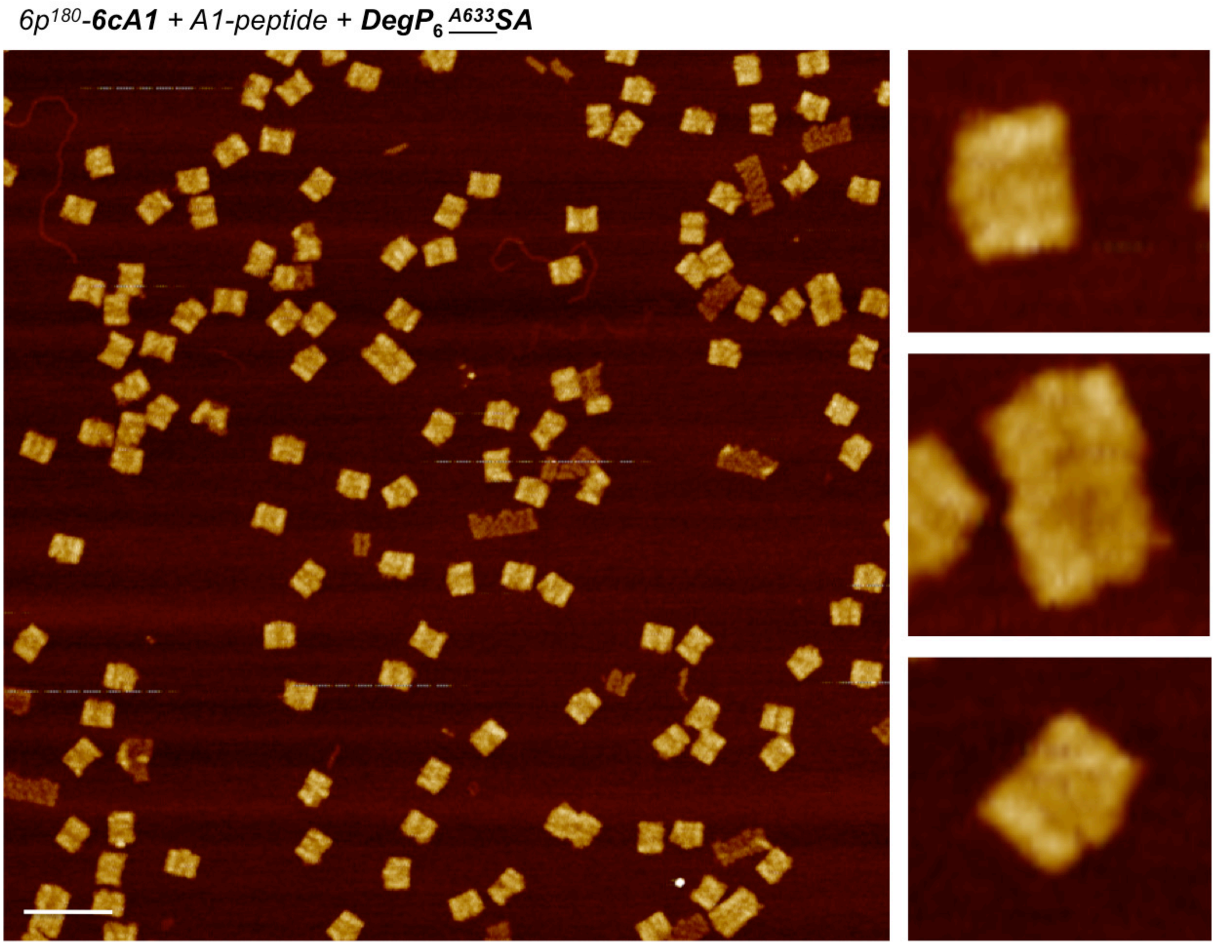

Supplementary Figure 50. AFM imaging of DegP$_6$$\underline{^{A633}}$SA binding to 6p^{180}-6cA1. Scale bar is 200 nm.

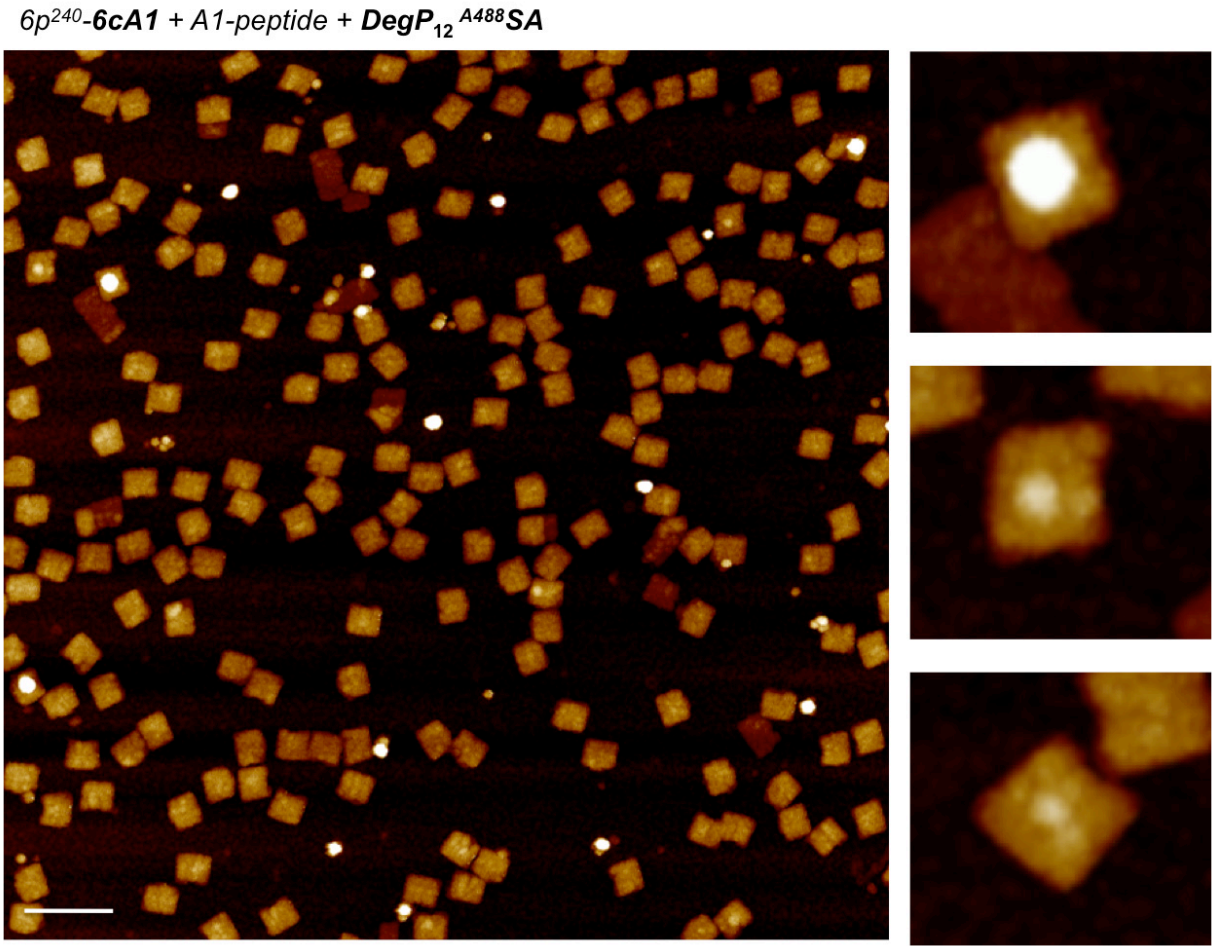

Supplementary Figure 51. AFM imaging of DegP$_{12}$^{A488}SA binding to 6p^{240}-6cA1. Scale bar is 200 nm.

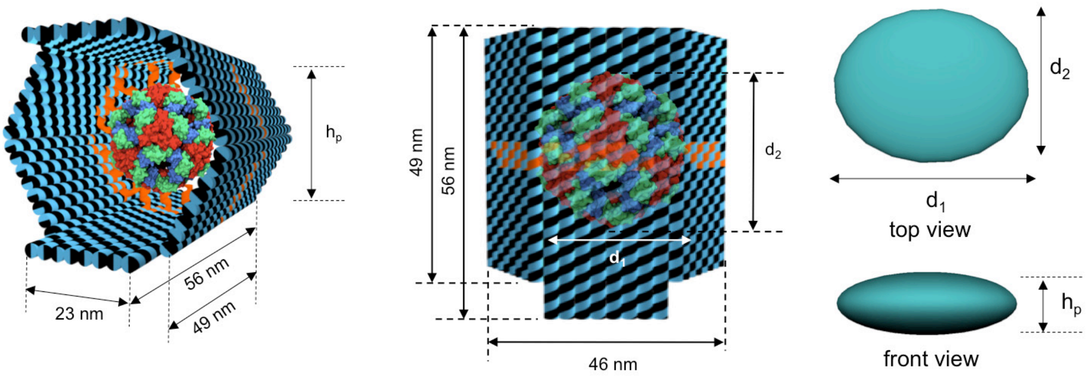

	theoretical values			$6p^{120}$-18cA1 + A1-peptide		
	6-mer	***12-mer***	***24-mer***	***6-mer***	***12-mer***	***24-mer***
d_1 (nm)	9	15	19	26 ± 5	32 ± 5	32 ± 5
d_2 (nm)				22 ± 4	29 ± 4	29 ± 4
h_p (nm)				4 ± 1	6 ± 1	7.5 ± 1
vol (nm^3)	4/3 π 91	4/3 π 422	4/3 π 857	4/3 π 286	4/3 π 696	4/3 π 870

Supplementary Figure 52. Analysis of DegP-loaded 6p constructs under AFM imaging in air. Loading the host with DegP leads to increase in the height profile of the structure. The contribution given by encapsulation of the protein is h_p. The total height profile of the complex (as in Figure 4f of the main manuscript) has been calculated as $h_p + h$, where h is the height profile of the empty cage (3.1 nm, Supplementary Fig. 44). We also measured the dimensions of the protein along the two axes (d_1 and d_2) and observed an ellipsoidal shape that may be attributed to the high compression forces in the imaging conditions. Analysis of the results allowed distinguishing three distributions, which may be reasonably attributed to the DegP in the three oligomerization states. The volumes occupied by the proteins have been calculated as $4/3\pi(d_1 d_2 h_p)$. The observed discrepancies between the theoretical ($4/3\pi r_{prot}^3$) and the experimentally observed volumes can be due to the extremely different conditions in which the dimensions of the proteins have been calculated. From the one side, theoretical dimensions are derived from X-ray structures; on the other side, AFM-observed values are very much dependent on the presence of the DNA envelope and surrounding peptide ligands, as well as on deformation forces.

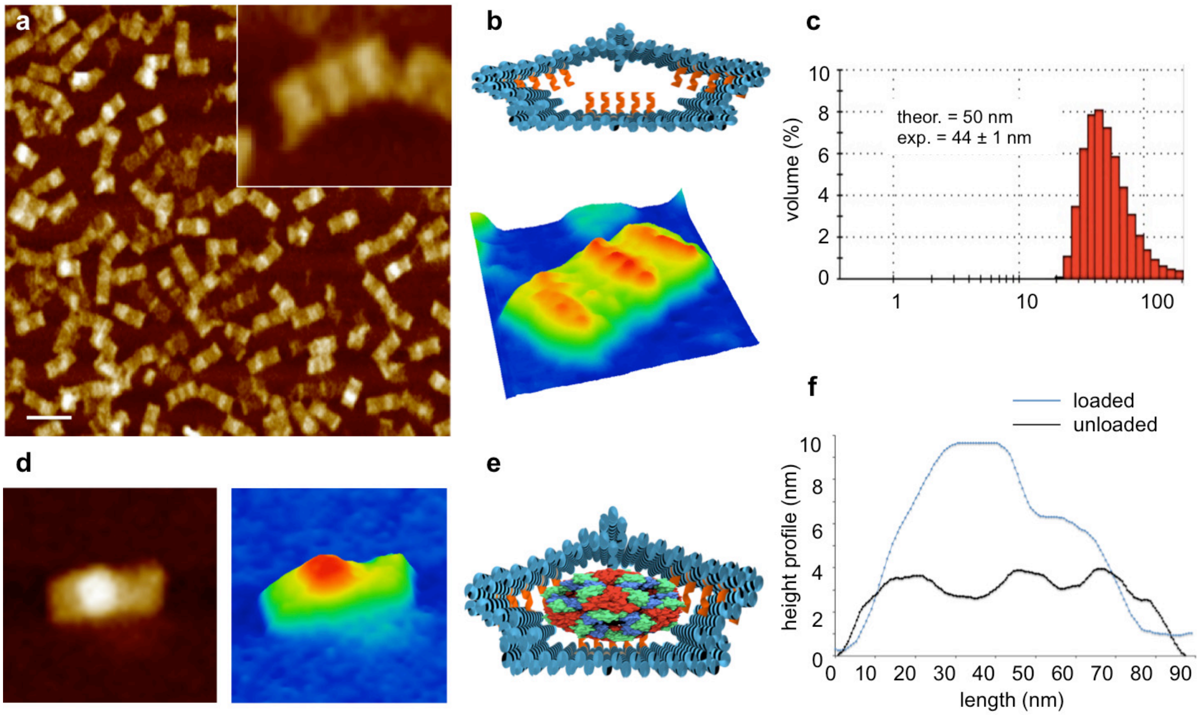

Supplementary Figure 53. AFM and DLS characterization of unloaded and DegP loaded 3p hosts. AFM characterization of the empty DNA chamber (**a**) results in compression of the structure, giving rise to a rectangular shape of variable width, same length as the 3prism and a height profile of ca. 3 nm displaying three features about 4 nm high (**b** and black curve in **f**). Nevertheless, DLS experiments indicate that the structure adopts the expected hollow shape in solution (**c**). When loaded with DegP, the structure displays a brighter spot in its center (**d**) associated to a higher height profile (blue curve in **f**), which we attribute to successful incorporation of the protein inside the chamber (**e**). This latter might be facilitated by the capability of the chamber to deform. Scale bar is 200 nm.

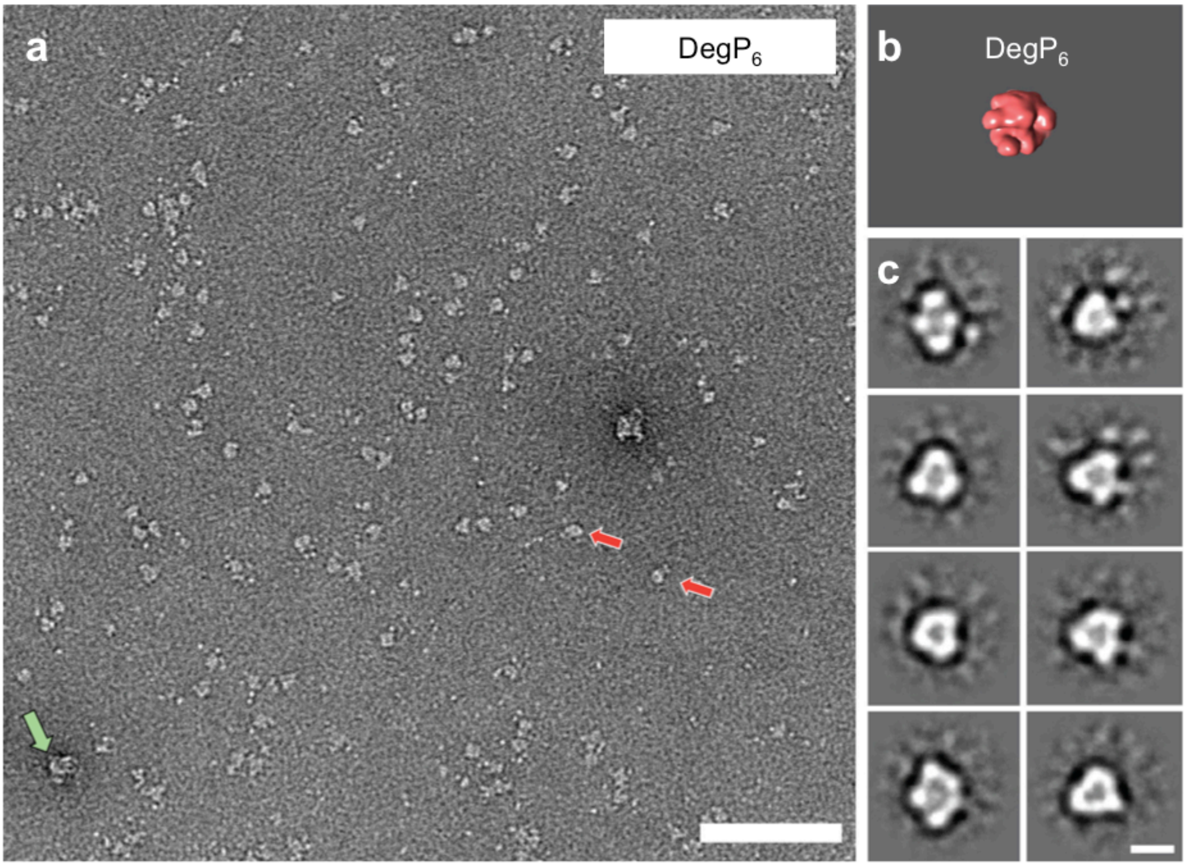

Supplementary Figure 54. TEM characterization of DegP$_6$. (**a**) Representative digital micrograph of negatively stained DegP$_6$. Scale bar 100 nm. (**b**) Simulated 3D model of DegP$_6$ from the respective crystal structure downfiltered to a resolution of 15 Å (PDB-ID 1ky9) and (**c**) representative class averages. Scale bar 10 nm.

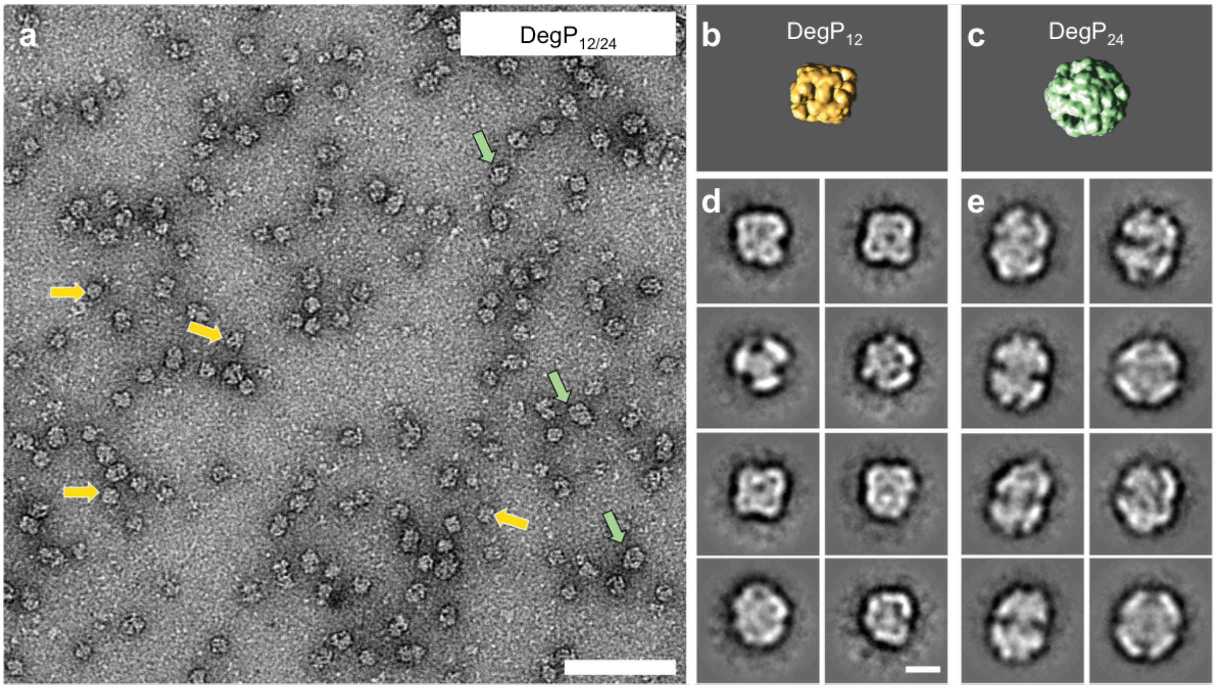

Supplementary Figure 55. TEM characterization of DegP$_{12/24}$. (**a**) Representative digital micrograph of negatively stained DegP$_{12/24}$. Scale bar 100 nm. Simulated 3D models of DegP$_{12}$ (**b**) and DegP$_{24}$ (**c**) from the respective crystal structures downfiltered to a resolution of 15 Å (DegP$_{12}$, PDB-ID 2zle; DegP$_{24}$, PDB-ID 3cS0) and representative class averages (respectively, in **d** and **e**). Scale bar 10 nm.

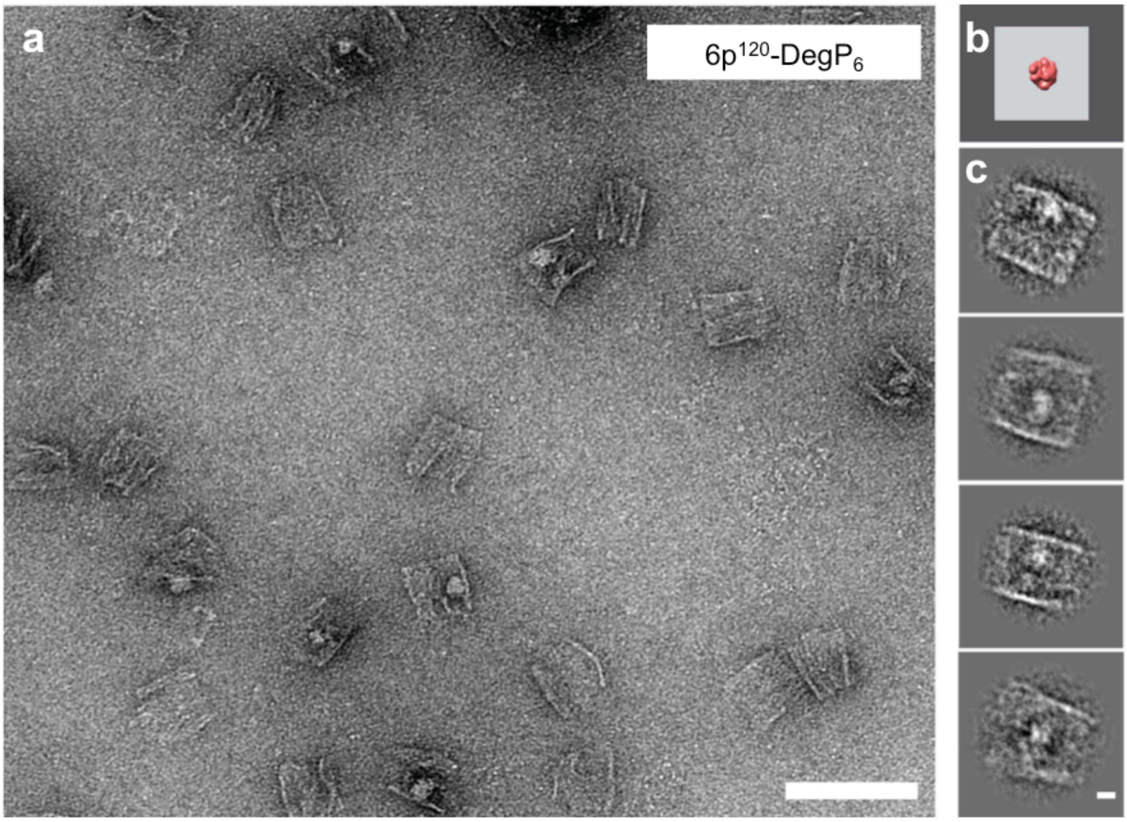

Supplementary Figure 56. TEM characterization of $DegP_6$ loaded DNA hosts. (**a**) Representative digital micrograph of negatively stained $DegP_6$-loaded 6p constructs. Scale bar 100 nm. (**b**) Simulated 3D model of the $6p^{120}$-$DegP_6$ complex and (**c**) representative class averages. Scale bar 10 nm.

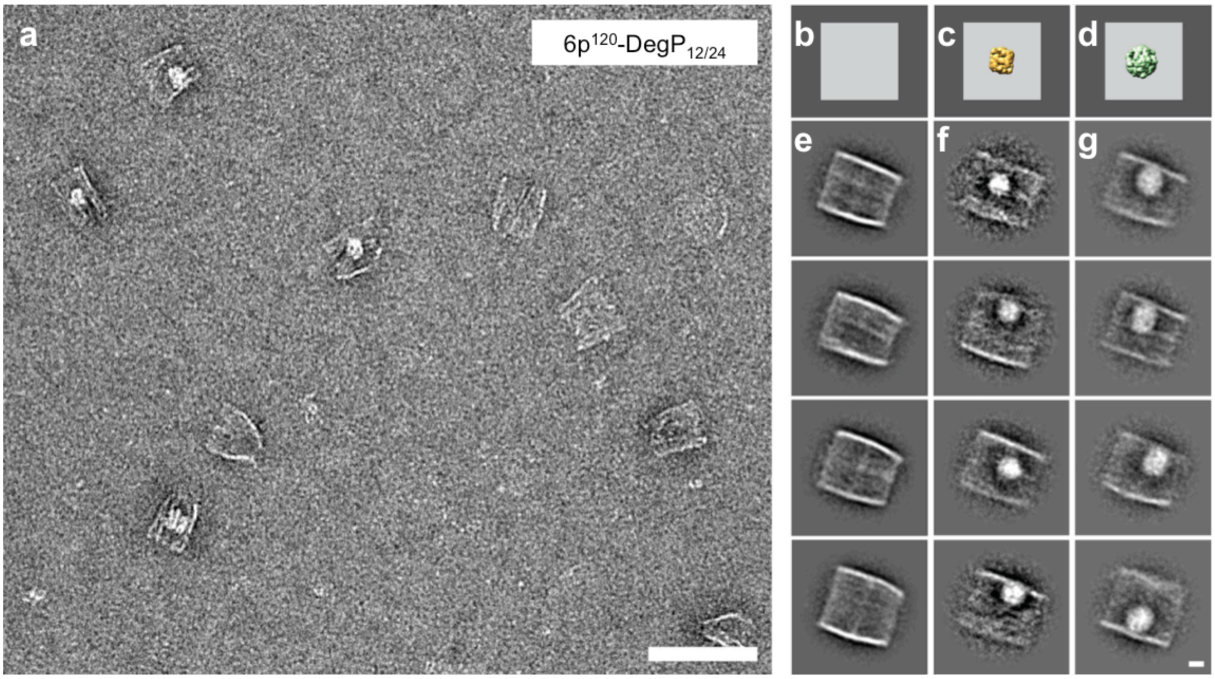

Supplementary Figure 57. TEM characterization of empty and $DegP_{12/24}$ loaded DNA hosts. (**a**) Representative digital micrograph of negatively stained $DegP_{12/24}$-loaded 6p constructs. Scale bar 100 nm. Simulated 3D models of the empty $6p^{120}$ (**b**), $6p^{120}$-$DegP_{12}$ complex (**c**) and $6p^{120}$-$DegP_{24}$ complex (**d**) and representative class averages (respectively, in **e**, **f** and **g**). Scale bar 10 nm.

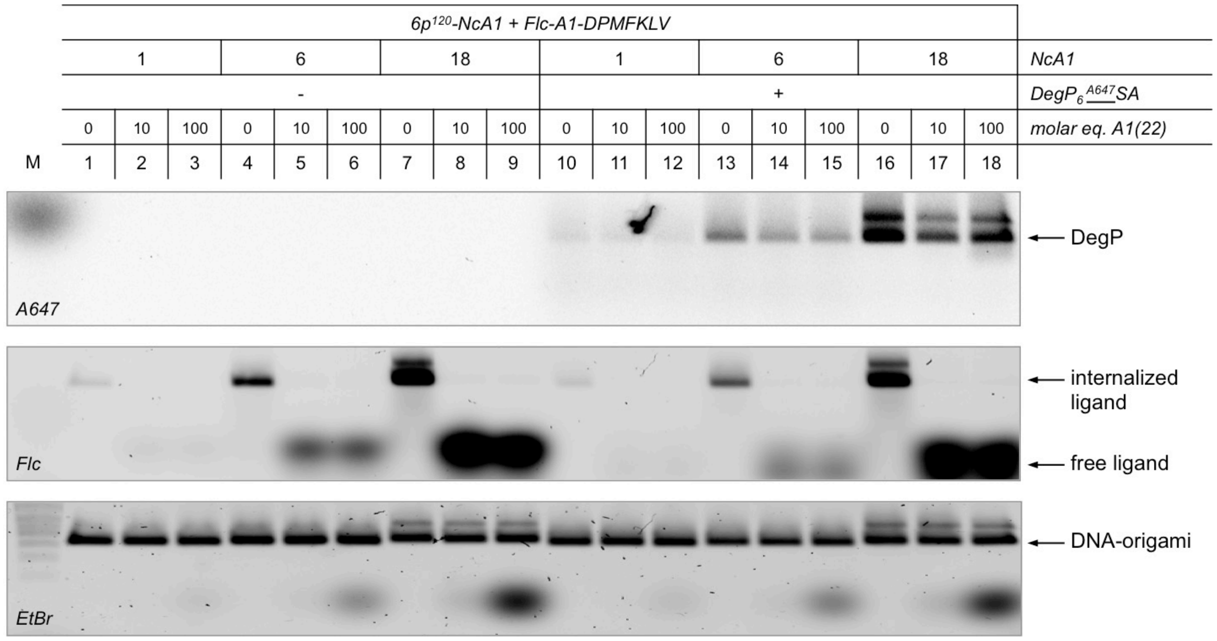

Supplementary Figure 58. Single-strand displacement on DegP$_6^{A647}$SA bound to distinct 6p^{120} hosts. Upon DNA origami assembly and loading with DegP, the samples were purified by agarose gel extraction and treated with 0, 10 or 100 equimolar amounts of A1(22) in respect to the number of cA1 arms present in the construct. The reaction was let at 30 °C overnight and the products were finally analyzed by agarose gel electrophoresis. Both in absence (lanes 1 to 9) and presence (lanes 10 to 18) of loaded DegP protein, displacement of the Flc-labeled DNA-peptide conjugates bound into the inner cavity of the host takes place completely, already upon addition of 10 equimolar amounts of A1 actuator strands (Flc-signal, lanes 2, 5 and 8, for the unloaded 6prism and lanes 11, 14 and 17 for the loaded 6prism). The successful occurrence of single-strand displacement is visible by the disappearance of the fluorescein signal from the origami band and appearance of a low molecular weight band, associated to the fluorescently labeled DNA-peptide conjugates free in solution. Interestingly, despite removal of the ligands, the DegP protein still remains trapped inside the DNA cage (A647 signal, lanes 10 to 18). In addition, *protein trapping is independent on the number of cA1 anchoring points* (lanes 11 and 12, for 1cA1; lanes 14 and 15 for 6cA1; and lanes 17 and 18 for 18cA1) *and from the added equivalents of A1(22)* (lanes 11-12, 14-15 and 17-18, for 10 and 100 equivalents, respectively for the 1cA1, 6cA1 and 18cA1 constructs). One can notice a slight decrease in the signal intensity of the protein upon addition of A1(22) effectors (cfr. lane 13 with lanes 14-15, or lane 16 with lanes 17-18), which might indicate a partial release of the protein from the DNA host; nevertheless most of the protein still keeps inside the DNA cage. Note that all DNA constructs have been prepared and loaded in the same initial concentrations and volumes as indicated by the identical intensity of the origami bands visible after ethidium bromide staining. A rough comparative estimation of the band intensities of the different products is therefore meaningful. Gel running conditions: 0.75% agarose in 1X TBEMg, at 80V for 2 hrs at 4°C. The gel was scanned with a Typhoon FLA 9000 at different wavelengths to record the presence of DegP protein (A647-labeled) and peptide ligand (Flc-labeled). DNA was visualized upon staining with ethidium bromide. Lane M contained a 1 kbp DNA ladder. The DNA origami migrates between the 1.5 and 2.0 kbp bands of the ladder.

6p120-NcA1 + Flc-A1-DPMFKLV												
1						6						NcA1
-			+			-			+			$DegP_6^{A633}SA$
0	10	100	0	10	100	0	10	100	0	10	100	molar eq. A1(22)
1	2	3	4	5	6	7	8	9	10	11	12	

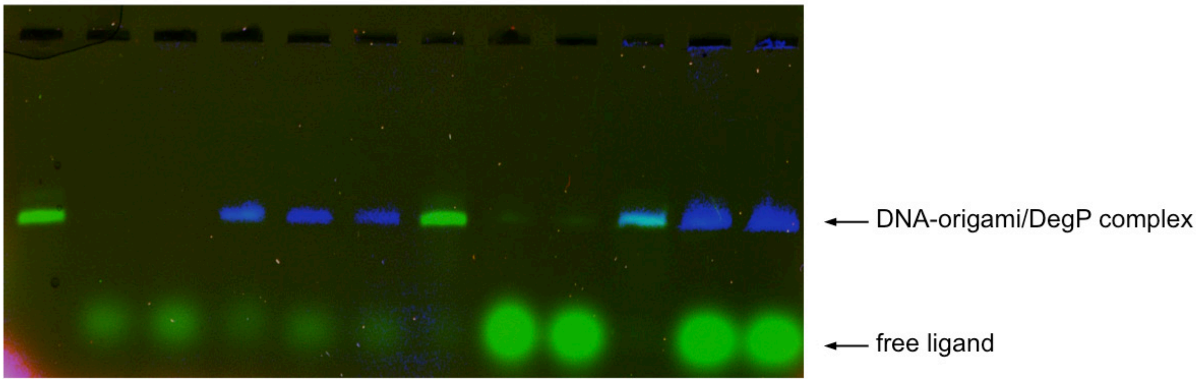

Supplementary Figure 59. Single-strand displacement on $DegP_6^{A633}SA$ bound to distinct $6p^{120}$ hosts. The same experiment as in Supplementary Fig. 58 was performed here, using $DegP_6$ labeled at selected cysteine residues with A633 instead of A647. This was done to verify whether the dyes – and thus their charge properties – might have an effect on the delivery of the internalized protein. The results demonstrate that this is indeed not the case. Both in presence of 1 (lanes 1-6) or 6 (lanes 7-12) inner pointing ligands, and both in presence of 10 (lanes 2, 5, 8 and 11) or 100 (lanes 3, 6, 9 and 12) equimolar amounts of A1(22) effector strands, the protein remains trapped inside the cage (blue bands in lanes 5, 6 and 11, 12). Gel composition and running conditions as in Supplementary Fig. 58.

6p^{120}-18cA1 + TAMRA-A1-DPMFKLV										
-		+								DegP$_6^{A647}$SA
-		-		+		-		+		50 eq. tweezers
-		-		-		+		+		50 eq. DPMFKLV
-	+	-	+	-	+	-	+	-	+	180 eq. A1(22)
1	2	3	4	5	6	7	8	9	10	

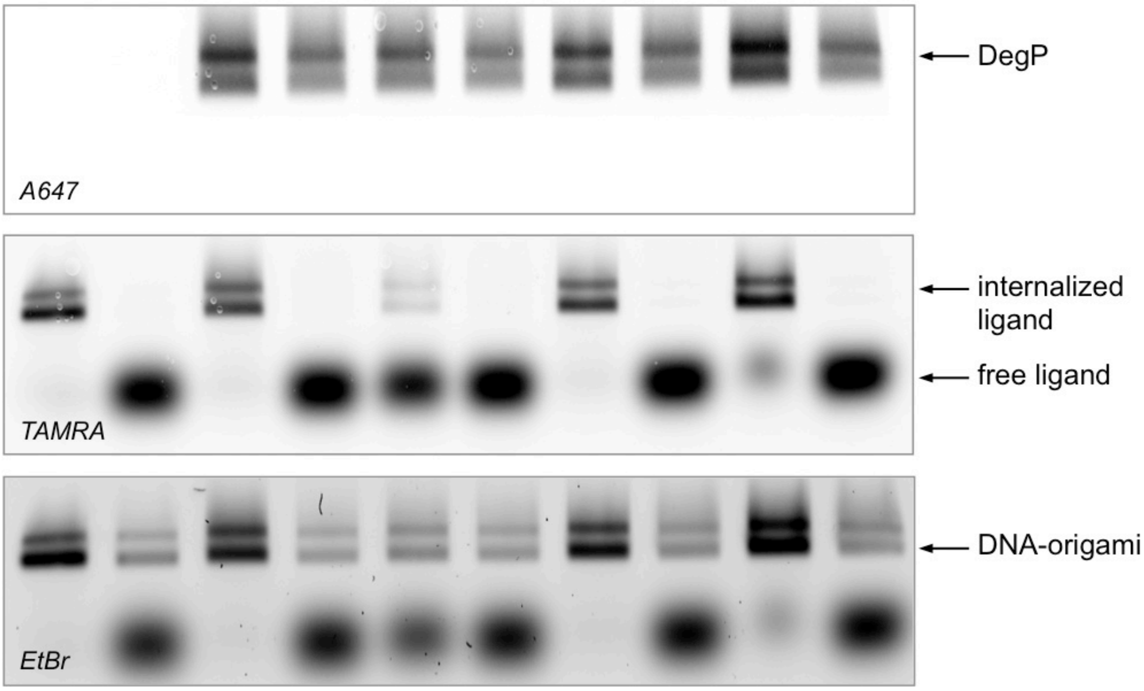

Supplementary Figure 60. Single-strand displacement in presence of added peptide substrate or tweezers. Upon DNA origami assembly and loading with DegP as usual, the samples were purified by agarose gel extraction and treated with 0 or 180 equimolar amounts of A1(22) effector strands. The displacement of the inner bound ligands was performed either in absence (lane 4) or presence (lane 6) of lysine-specific molecular tweezers (50 eq.); alternatively 50 eq. of the DPMFKLV peptide were also added to the solution mixture either in absence (lane 8) or presence (lane 10) of tweezers. As shown by the A647 signal, the protein is still trapped inside the cage, although the decreased band intensity suggests its partial escape. Remarkably, ligand displacement successfully occurs in all cases, as demonstrated by the disappearance of the TAMRA signal from the DNA origami band and simultaneous appearance of a new TAMRA-visible band with low molecular weight and higher electrophoretic mobility (lanes 4, 6, 8 and 10). The loss of the DNA-peptide conjugates from the inner cavity of the host is also visible from the decreased intensity of the DNA origami bands upon ethidium bromide staining and appearance of a low molecular weight band associated to the free ligands in solution (lanes 2, 4, 6, 8 and 10). This experiment demonstrates that *even in presence of Lys-specific molecular tweezers and competitive PDZ1 linkers, the delivery of the protein from the DNA host is still not complete*. This suggests that other sets of forces keep the protein firmly anchored to the inner surface of the DNA cage and that these forces are not depleted in the conditions used until now. Gel composition and running conditions as in Supplementary Fig. 58.

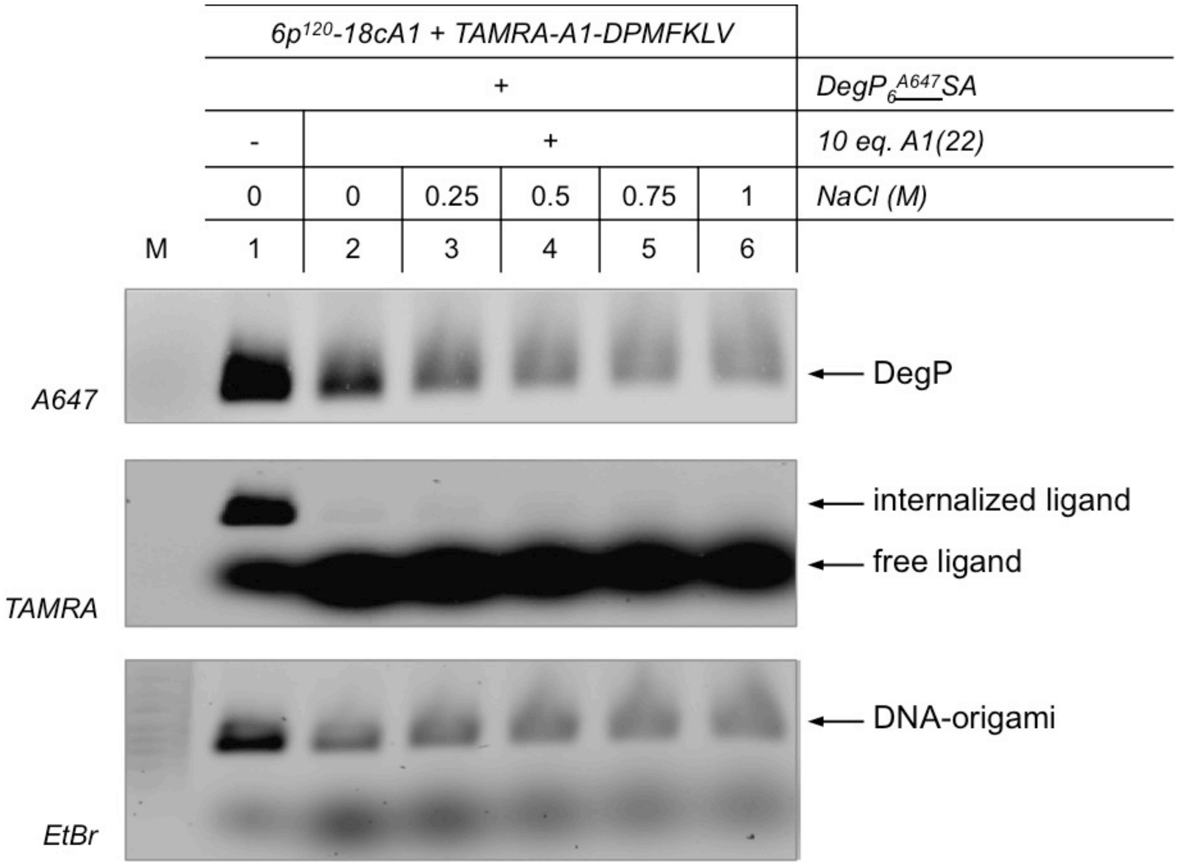

Supplementary Figure 61. Single-strand displacement at increasing NaCl concentrations. In this experiment we tested the effect of increased ionic strength on the displacement reaction. Again, addition of 10 equimolar amounts of A1(22) in absence of sodium ions results in complete release of the TAMRA-labeled inner ligands (cfr. lanes 1 and 2, TAMRA signal). This is still valid for increasing sodium concentrations, from 0.25 M to 1 M (lanes 3 to 6). Nevertheless, the DegP protein signal is still present (A647 panel, lanes 3 to 6) indicating incomplete protein escape. Note that the presence of high salt concentrations in the reaction mixture affects the migration properties of the DNA/protein samples, leading to broader and less-defined gel bands. In spite of this, one can conclude that *even at high ionic strength conditions, protein delivery does not occur in a satisfactory yield*. Lane M contained a 1 kbp DNA ladder. The DNA origami band (lane 1) migrates between the 1.5 and 2.0 kbp bands of the ladder. Gel composition and running conditions as in Supplementary Fig. 58.

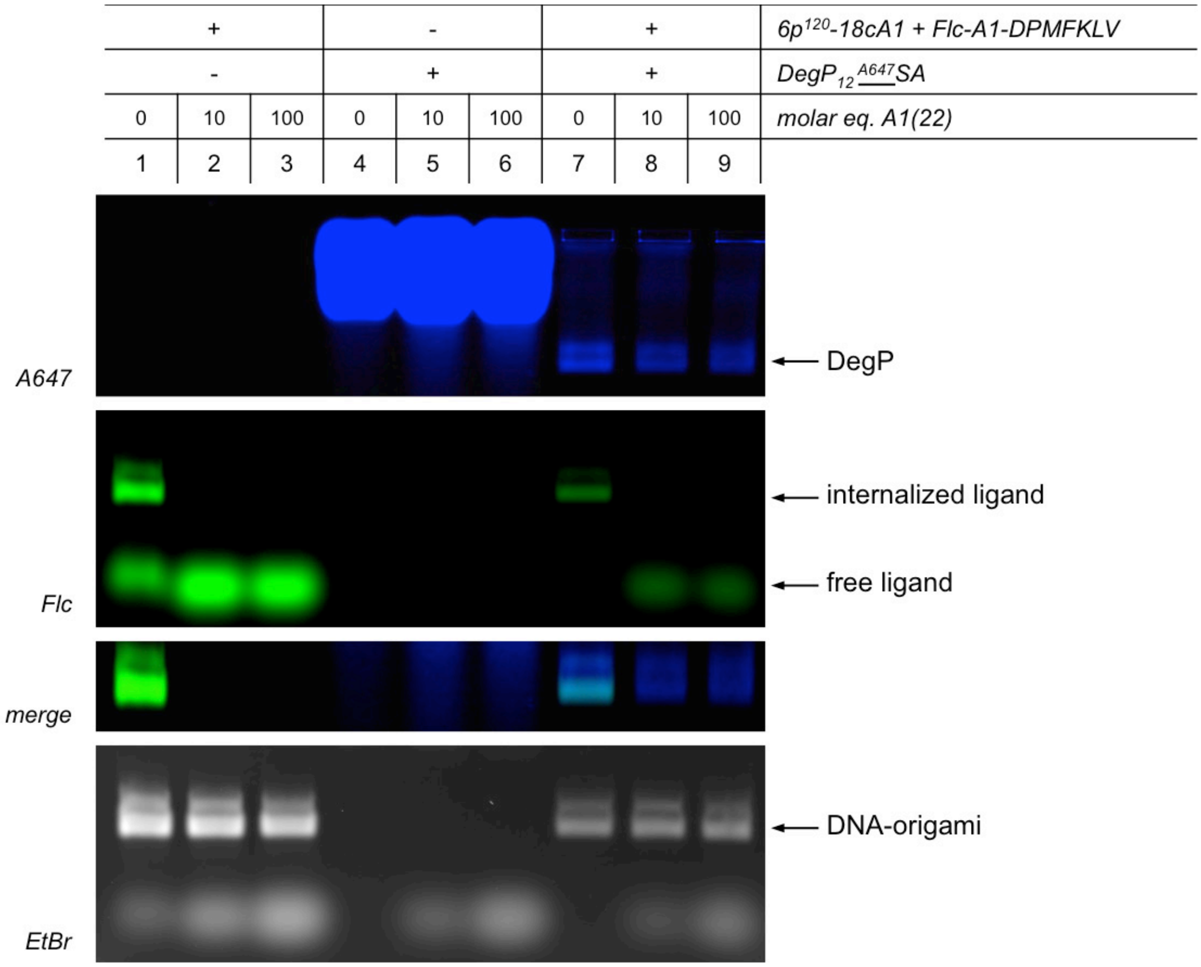

Supplementary Figure 62. Single-strand displacement on $DegP_{12}$$^{\underline{A647}}$SA bound to $6p^{120}$-18cA1. In this experiment we wanted to test whether a protein of larger size can be more easily displaced. At this purpose, the $DegP_{12}$ protein was encapsulated inside the 6p construct, the complex was then purified and treated with 0, 10 or 100 molar equivalents of A1(22) actuator strands (lanes 7 to 9). Control samples lacking the protein (lanes 1-3) or the DNA host (lanes 4-6) were also analyzed for comparison. The results show that *protein size does not matter: although ligands are fully displaced, the protein keeps trapped inside the host* (A647 panel, lanes 8 and 9). Gel composition and running conditions as in Supplementary Fig. 58.

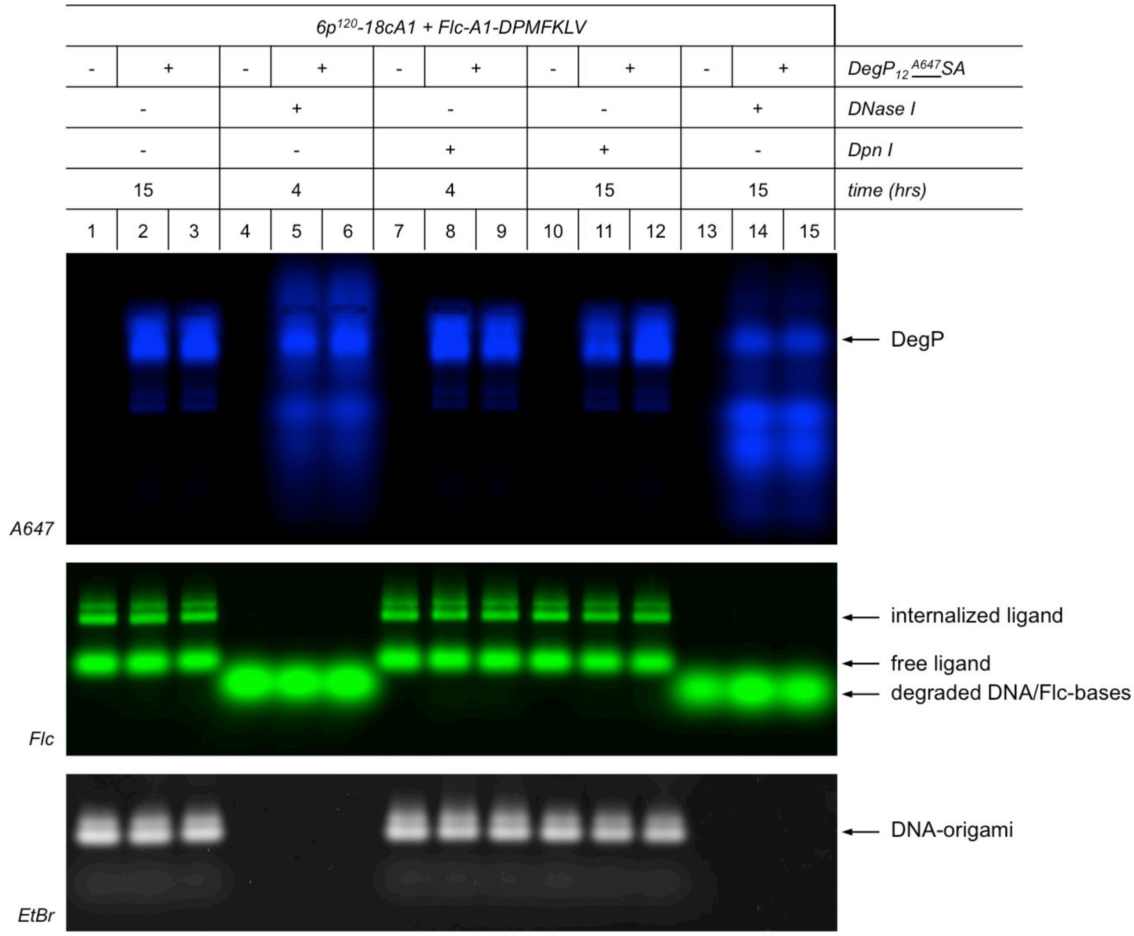

Supplementary Figure 63. Digestion of the DNA cage by DNaseI (I). A 6p^{120}-18cA1 construct internally modified with Flc-labeled DNA-peptide ligands either in absence (lane 1) or presence of the DegP$_{12}$$\underline{^{A647}}$SA (lanes 2 and 3) was treated for 4 hrs with DNaseI (lanes 4 to 6) or DpnI (lanes 7 to 9). The same reaction mixture was analyzed after 15 hrs incubation (DpnI: lanes 10 to 12 and DNaseI: lanes 13 to 15). Samples containing the enzymes were done in duplicates to check for reproducibility of the results. The data obtained show that DpnI has no effect on both the DNA and the protein (lanes 7-12). This is expected, as DpnI digests only methylated DNA. On the other hand, addition of DNaseI leads to full degradation of the DNA origami cage, as evidenced by the disappearance of the bands corresponding to the DNA origami constructs (lanes 4-6 and 13-15, upon ethidium bromide staining). The Flc molecules attached to the terminal nucleobases of the DNA-peptide linkers are visible as fast migrating bands (Flc panel, lanes 4-6 and 13-15). Interestingly, treatment with DNaseI leads to the appearance of several DegP subproducts of faster migration rate, whose intensity increases after 15 hours incubation (cfr. lanes 5 and 6 with 14 and 15). We attribute this to a partial degradation of the protein in the experimental conditions used, which could be a consequence of the specific DNaseI buffer. To better understand this point, another digestion experiment was performed using the protein alone as control (Supplementary Fig. 64).

+	-	+	+	-	+	-	+	*$6p^{120}$-18cA1 + Flc-A1-DPMFKLV*
-	+	+	+	+	-	+	+	*$DegP_{12}$ $\underline{A647}$SA*
-			+					*DNase I*
0			4		15			*time (hrs)*
1	2	3	4	5	6	7	8	

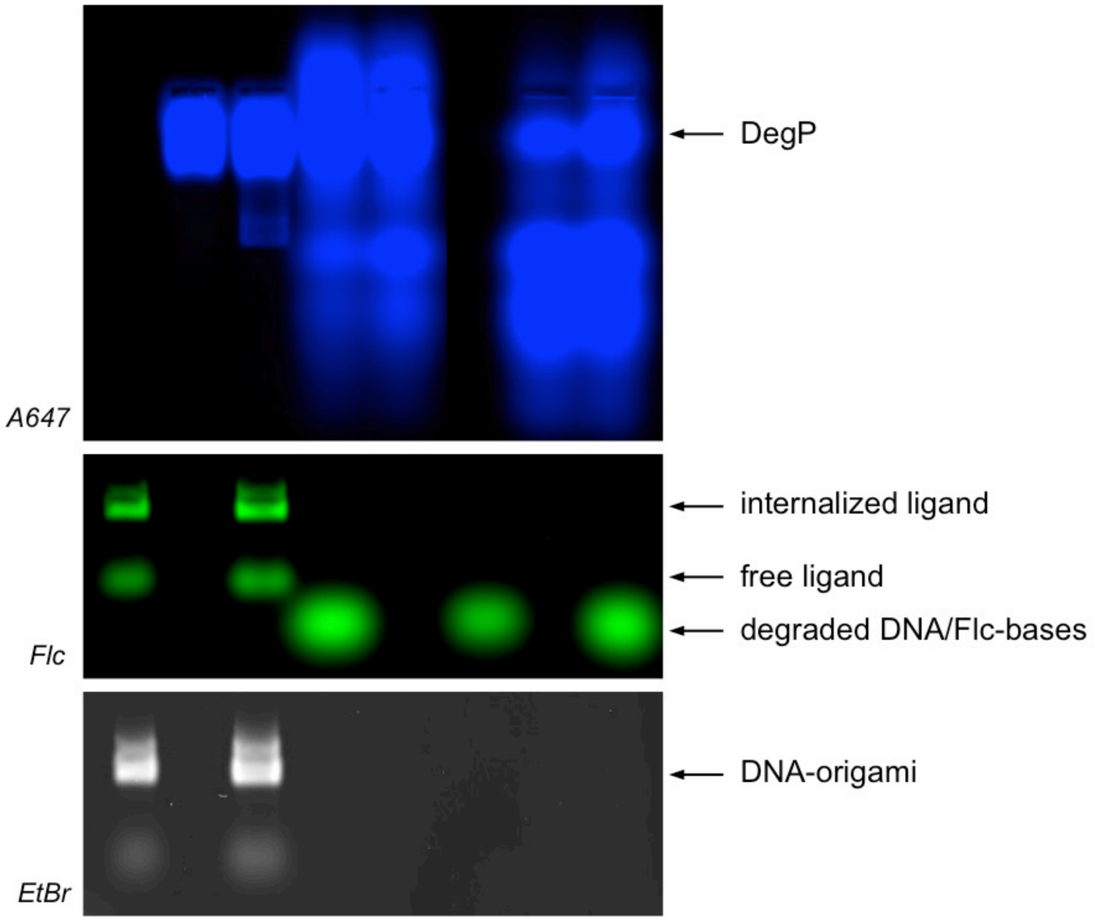

Supplementary Figure 64. Digestion of the DNA cage by DNaseI (II). The same experiment as described above in Supplementary Fig. 63 was performed here, adding the suitable controls: the DNA origami cage, DegP protein and their complex, both in absence (respectively, lanes 1 to 3) and presence (lanes 6 to 8) of DNaseI (after 15 hours incubation). Both the DNA-DegP complex and the protein alone were also analyzed after 4 hours enzymatic treatment (lanes 4 and 5). As visible in lanes 5 and 7, the DegP protein degrades when treated with DNaseI, probably as a consequence of the salts present in the buffer. This degradation increases with time as evidenced by the appearance of a higher amount of low molecular weight products after 15 hrs treatment (cfr. lanes 4 and 5 with 7 and 8). Nevertheless, the DNA origami host is completely digested already after only 4 hours (lane 4). Although more investigations are necessary to understand and improve this issue, *the enzymatic digestion of the DNA cage could indeed represent a method to liberate the protein*.

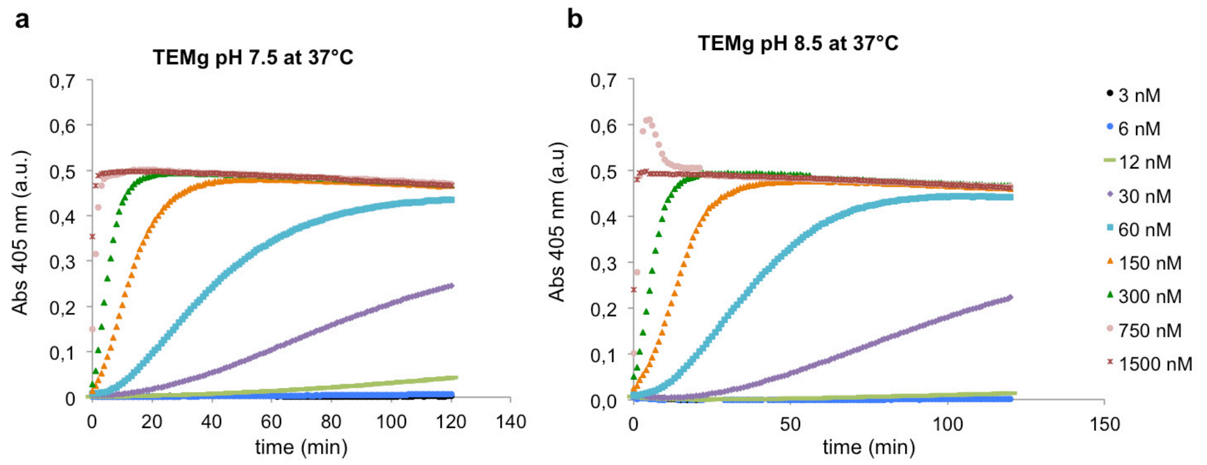

Supplementary Figure 65. Activity of DegP-WT at different protein concentrations. DegP was diluted in the buffer used for DNA origami assembly and manipulation (TEMg 1X) and adjusted to pH 7.5 (**a**) or 8.5 (**b**). The enzymatic reaction was performed at 37 °C using different protein concentrations, ranging from 3 nM to 1500 nM, and was initiated upon addition of 1 µL of 50 µM peptide substrate DPMFKLV-pNA, resulting in a final substrate concentration of 500 µM. DMSO in the reaction mixture was 1%. The results obtained showed no evident difference between the two buffers used. In addition, below 12 nM protein concentration no significant signal is detectable after 2 hours (*green curves*). Best values of protein concentration for reliable activity tests are around 60-150 nM (*blue and orange curves*). This would correspond to a final concentration of DNA-$DegP_6$ (1:1) complex of about 25 nM. Current protocols for purification of DNA origami structures allow to reach final concentrations till 50-100 nM, although often in limited volumes (ca. 100 µl). The problem could be easily surpassed by large-scale origami preparation; nevertheless two major drawbacks still need to be faced: the low yield in formation of DNA-protein complexes and their purification, preserving not only the "weak" non-covalent binding interactions but also protein activity. Please note that in these assays the nominal protein concentration refers to the concentration of the monomer, such that for example 150 nM DegP would correspond to 25 nM of the protein in its hexameric form. Also, DegP in its active form and upon substrate binding undergoes conformational changes, which are believed to start oligomerization transformations into higher order structures. Thus, although starting from a known oligomerization state of DegP, this may change during the course of the enzymatic reaction into a mixture of different oligomers. In the following we will therefore refer to it simply as DegP-WT.

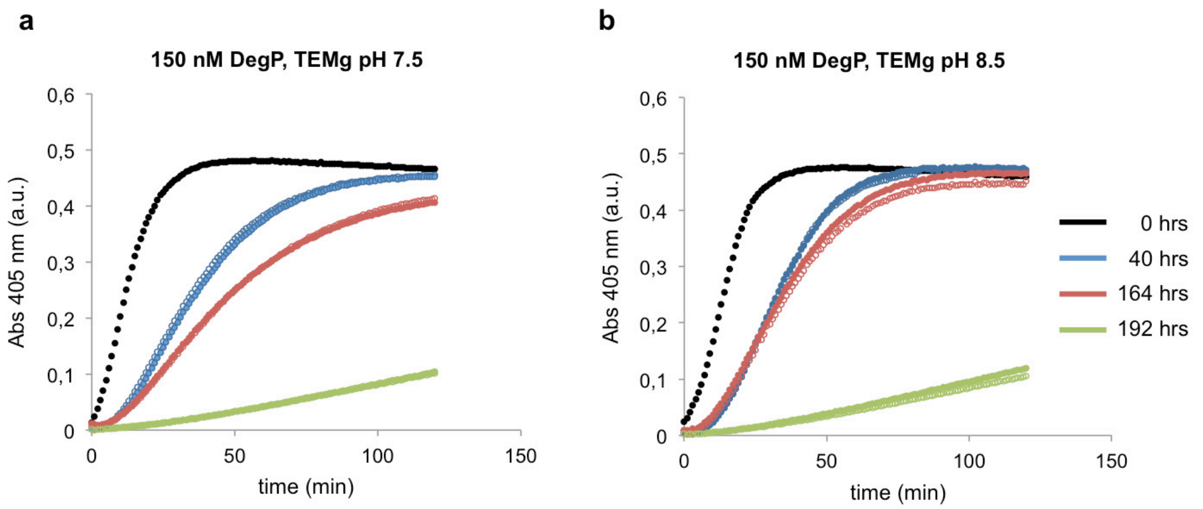

Supplementary Figure 66. Activity of DegP-WT at different time points. In this experiment we tested the stability of DegP over time, in order to set up the conditions for future assays. At this purpose, DegP was diluted in TEMg 1X, at pH 7.5 (**a**) or 8.5 (**b**) and incubated at RT. Aliquots were taken at different time points and the enzymatic activity was measured for 2 hrs at 37 °C upon addition of DPMFKLV-pNA substrate. These results indicate that a sufficiently high concentration in DegP (around 150 nM) is necessary to measure a proteolytic activity after 2-3 days incubation (*blue and red curves*). This is in general the minimal manipulation time required to load the DNA origami host with the protein, purify the complex and characterize it.

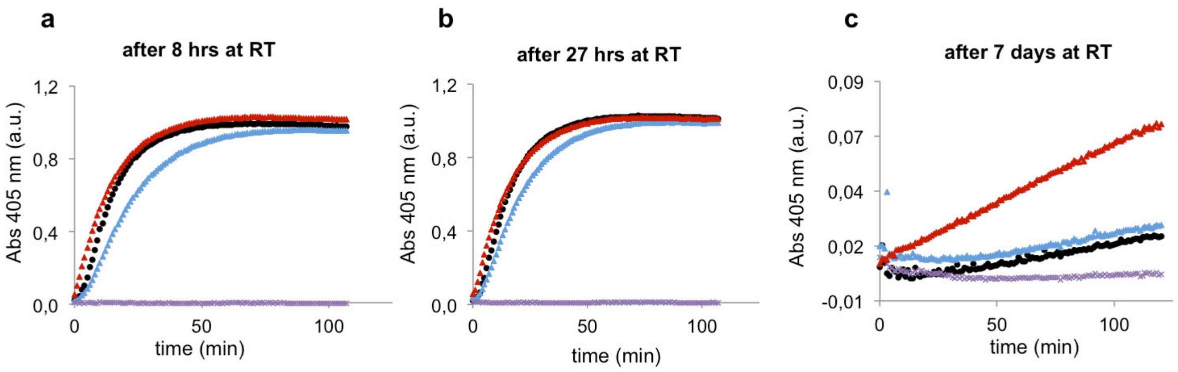

	specific activity (nmol mg^{-1} min^{-1})			
	DegP-WT			
	+			-
**time**	-	$6p^{120}$-18cA1	$6p^{120}$-18cA1/ A1-DPMFKLV	$6p^{120}$-18cA1 + A1-DPMFKLV
8 hrs	1713.4	901.6	1548.3	0
27 hrs	1548.9	1063.7	1481.4	0
7 days	6.3	6.7	18.9	0

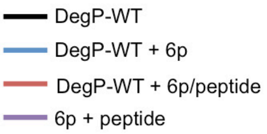

Supplementary Figure 67. Stability of DegP-WT both in presence and absence of the DNA host. 150 nM DegP was incubated at RT either in absence (*black* curve) or presence of the 6p host, which was lacking (*blue*) or bearing (*red*) the peptide linkers for protein recognition and loading. As negative control a mixture of 6p and linkers was used (*violet* curve). The activity of DegP at different time points (8 hrs, 27 hrs and 7 days; **a** to **c**) was measured for 2 hrs at 37 °C upon addition of DPMFKLV-pNA substrate. The results showed a similar behavior for all DegP-containing samples in the first 2 days incubation (a and b), indicating that within this time range the stability of DegP (at 150 nM concentration) is not affected by the presence and/or binding to the DNA origami host. Interestingly, after one-week incubation, the stability of the DegP protein is notably enhanced by the binding to the 6p construct functionalized with the peptide linkers (*red curve* in c). This suggests that encapsulation of the protein inside the DNA cage might indeed protect it from degradation. The stability of the protein in these conditions appears to be about three-fold higher (18.9 nmol mg^{-1} min^{-1}) than that of the protein alone (6.3 nmol mg^{-1} min^{-1}) or in presence of the DNA cage lacking the recognition motifs (6.7 nmol mg^{-1} min^{-1}). Although already promising, these results should be verified again for reproducibility and DNA samples analyzed should be previously purified and their concentration exactly determined. Only in this way, a full quantitative analysis can be performed and differences in behavior better evidenced. Nevertheless, such preliminary assays show that in the conditions tested the DNA host system indeed affects and improves DegP stability.

Supplementary Tables

Supplementary Table 1. **DegP proteins used in this work.** The different types of DegP proteins used are distinguished according to their oligomerization state (indicated by a subscript number: 6, 12 or 24), as well as by the absence (WT) or presence (SA) of the S210A mutation at the active site. WT indicates the catalytically active, wild-type form; whereas, SA indicates the mutated inactive form. Additionally, fluorescently labeled proteins will be indicated by an A488, A633 or A647 at the apex, corresponding respectively to the Alexa-like fluorophores with 488, 633 and 647 excitation wavelength (the color code used to highlight the name of the protein corresponds to the color of the dye under visible light illumination and is consistently used in the description of every agarose gel). Underlined fluorophore names denote attachment of the dye at genetically introduced cysteine (rather than lysine) residues.

**name**	***n*-mer**	**mutations**	**labeled aa**	**dye**		
				A488	A633	A647
DegP$_{6}^{A488}$WT	6-mer	-	Lys	+	-	-
DegP$_{6}$SA		S210A (protease)	-	-	-	-
DegP$_{12}$SA	12-mer		-	-	-	-
DegP$_{12}^{A488}$SA			Lys	+	-	-
DegP$_{24}^{A488}$SA	24-mer			+	-	-
DegP$_{6}^{\underline{A633}}$SA	6-mer	S210A<br>C57A<br>C69A<br>N146C (protease)<br>N296C (PDZ1)<br>A414C (PDZ2)	Cys	-	+	-
DegP$_{6}^{\underline{A647}}$SA				-	-	+
DegP$_{12}^{\underline{A488}}$SA	12-mer			+	-	-
DegP$_{12}^{\underline{A647}}$SA				-	-	+
DegP$_{24}^{\underline{A488}}$SA	24-mer			+	-	-
DegP$_{24}^{\underline{A647}}$SA				-	-	+

Supplementary Table 2. **Nomenclature of the DNA hosts used in this work.** Two kinds of protein cages have been analyzed: a hexagonal (6p) and a triangular (3p) prism. The angle imposed between adjacent faces has been indicated by an apex. For the 6p, 120 and 240 indicate, respectively, an orientation of the protruding arms (PAs) towards the interior or the exterior of the cavity. 180 describes instead an undefined orientation of the PAs. The 3p construct can fold only with fixed 60° angles between adjacent faces, due to the different pattern of crossovers used in the design of this structure. The number of PAs (i.e. cA1 sequences) available for functionalization of the chambers were for the 6p: 0, 6 (one per face) or 18 (three per face); while for the 3p were 6 (two per face) or 15 (five per face).

	name	face-to-face connections	n° cA1
6 prism	6p-nc	not connected	0
	$6p^{120}$-0cA1	120°	0
	$6p^{120}$-6cA1		6
	$6p^{120}$-18cA1		18
	$6p^{180}$-0cA1	120°/240°	0
	$6p^{180}$-6cA1		6
	$6p^{180}$-18cA1		18
	$6p^{240}$-0cA1	240°	0
	$6p^{240}$-6cA1		6
3 prism	$3p^{60}$-6cA1	60°	6
	$3p^{60}$-15cA1		15

Supplementary Table 3. Selected distances between the PDZ1 domains and the bound peptide. Binding of the peptide to the PDZ1 domain occurs by β-sheet extension. The distances $V7_{OT}$ – $G266_{HN}$ and $K5_{HN}$ – $G269_{O}$ describe the interaction between the peptide in the extended β-sheet and the backbone atoms of the PDZ1 domain. The distance $K5_{NZ}$ – $Q271_{CD}$ describes the salt bridge formed between K5 of the peptide and Q271 of the PDZ1 domain. See Supplementary Fig. 22 for details about the composition of systems 22c, 22l, 32c and 32l.

Distance	22c				
$V7_{OT}$ – $G266_{HN}$	2.45 ± 0.61	2.07 ± 0.48	2.04 ± 0.42	2.95 ± 1.38	-
$K5_{HN}$ – $G269_{O}$	1.94 ± 0.14	1.96 ± 0.18	2.69 ± 0.80	2.27 ± 0.56	-
$K5_{NZ}$ – $Q271_{CD}$	3.32 ± 0.15	3.36 ± 0.18	11.31 ± 3.50	3.48 ± 0.27	-
	22l				
$V7_{OT}$ – $G266_{HN}$	1.94 ± 0.20	2.04 ± 0.24	2.08 ± 0.34	2.02 ± 0.31	-
$K5_{HN}$ – $G269_{O}$	1.88 ± 0.12	1.96 ± 0.14	1.94 ± 0.17	1.98 ± 0.16	-
$K5_{NZ}$ – $Q271_{CD}$	3.42 ± 0.41	3.53 ± 0.22	7.52 ± 1.99	3.30 ± 0.13	-
	32c				
$V7_{OT}$ – $G266_{HN}$	1.91 ± 0.25	2.03 ± 0.25	1.91 ± 0.22	1.96 ± 0.20	1.95 ± 0.21
$K5_{HN}$ – $G269_{O}$	2.03 ± 0.20	1.97 ± 0.17	2.01 ± 0.27	1.92 ± 0.15	1.92 ± 0.16
$K5_{NZ}$ – $Q271_{CD}$	8.06 ± 4.17	3.38 ± 0.17	4.56 ± 1.94	3.60 ± 0.14	3.53 ± 0.21
	32l				
$V7_{OT}$ – $G266_{HN}$	1.99 ± 0.25	2.18 ± 0.51	1.99 ± 0.42	1.90 ± 0.16	2.43 ± 0.37
$K5_{HN}$ – $G269_{O}$	1.90 ± 0.14	1.89 ± 0.15	1.89 ± 0.12	2.14 ± 0.24	1.92 ± 0.15
$K5_{NZ}$ – $Q271_{CD}$	3.55 ± 0.16	8.10 ± 2.58	3.65 ± 0.21	13.46 ± 2.72	5.66 ± 0.97

Supplementary Table 4. Estimated values of I (protein particles sec^{-1}). These values were calculated for the three DegP oligomers diffusing inside the 6p or 3p constructs and considering the free room available in the host cavity. Note that in the 3p construct, as $R_{free} < r_{prot}$, the observed binding events can be explained only by the partial deformation of the DNA origami host.

	**$DegP_6$** r_{prot} = 4.5 nm	**$DegP_{12}$** r_{prot} = 7.5 nm	**$DegP_{24}$** r_{prot} = 9.5 nm
**6prism** R_{free} = 9.6 nm	354	212	167
**3prism** R_{free} = 3 nm	111	66	52

Supplementary Table 5. DNA-ligand conjugates and corresponding protein targets used in this work.

ligand	target
A1-DPMFKLV	DegP
Flc-A1-DPMFKLV	
TAMRA-A1-DPMFKLV	
A1-biotin	streptavidin
A1-Flc	anti-Flc^{CF594}
	anti-Flc^{CF640R}

Supplementary Table 6. AFM and TEM yields of structure formation and protein loading.

		120°			180°			240°	
ncA1		*0*	*6*	*18*	*0*	*6*	*18*	*0*	*6*
AFM yield (%)	no DegP	92.5	-	-	82.7	-	-	94.9	-
	$DegP_6$	-	5.8	6.9	-	0	-	-	0
	$DegP_{12/24}$	-	12	20	-	2.9	-	-	1
TEM yield (%)	no DegP	quantitative	-	-	-	-	-	-	-
	$DegP_6$	-	-	4	-	-	-	-	-
	$DegP_{12/24}$	-	-	26	-	-	-	-	-

Yields expressed in percentage were calculated by manually counting the target structures over the total of (correctly) folded structures, as described in Supplementary Methods.

Supplementary Note 1. Theoretical considerations on molecular diffusion

Molecular diffusion within the inner cavity of the DNA origami chamber is a fundamental prerequisite for efficient protein loading. Considering the protein as a spherical particle of radius r_{prot}, its diffusion coefficient (D) is described by the Stokes-Einstein equation:

$$D = \frac{K_B T}{6\pi\eta r_{prot}} \qquad (\mathrm{m^2\ s^{-1}}) \qquad (1)$$

where K_B is the Boltzmann's constant (1.38×10^{-23} J K^{-1}), T is the absolute temperature in Kelvin (298 K) and η is the viscosity of water at room temperature (0.001 kg s^{-1}m^{-1}). Of course, the smaller the hydrodynamic radius of the molecular cargo, the higher will be its diffusivity. In a typical experiment (30 μl of a 20 nM origami solution), 10-fold equivalent of protein are added for encapsulation inside the tubular structure, leading to a concentration C of about 10^{20} protein particles m^{-3}. The flux (**I**) of protein particles through the two extremities of the tubular structure is therefore described as:

$$I = \frac{-DC}{R_{free}} 2A \quad (\text{particles s}^{-1}) \qquad (2)$$

where R_{free} is the accessible ("free") inner radius of the tubular structure and A is the surface area of its entrance (approximated as πR_{free}^2). Substituting with Supplementary Equation (1) and numerical values above, one gets:

$$I = 166 \frac{R_{free}}{r_{prot}} \quad (\text{particles s}^{-1}) \qquad (3)$$

From Supplementary Equation (3) it is therefore clear that at defined conditions of temperature and buffer, the flux of protein particles through the tubular structure is proportional to the size of the tubular entrance and to the inverse of the protein radius. This flux of particles, in turn, affects the number of binding events occurring within the interior of the nanochannel in a certain interval of time. The estimated values of **I** for the three different oligomers of DegP in the two channels are reported in Supplementary Table 4.

Considering the free room available upon functionalization of the inner cavity, the 3prism would in principle exclude the binding of all three DegP forms. Nevertheless, binding of the $DegP_{12}$ inside the 3p construct was indeed observed (see Supplementary Figs. 42 and 53). We therefore believe that the DNA origami cages and the inner DNA-linker corona possess a certain degree of structural flexibility that enables their partial deformation and thus better adaptation to the size of the protein cargo. In spite of this, the 6p construct showed size selectivity towards the 12-mer, suggesting that such deformation is limited to a certain extent and does not lead to complete destruction of the structure.

Supplementary Discussion

Face-to-face connecting staples

Three-dimensional hollow structures made of DNA origami can be in principle constructed by linking together the edges of adjacent DNA origami sheets at defined dihedral angles, in order to achieve the desired target geometry with sufficient structural stability. Some examples of origami nanochannels have been reported in the literature and include cubes,[1] barrels[2,3] and prisms[4] of different cross sections and lengths. Among these, multilayer structures are particularly robust, due to the extended three-dimensional network of crossovers connecting the constituent faces of the chamber.[3] Because the M13 scaffold used for folding has a limited length (7249 bases), using most of it for creating multilayer faces inevitably reduces the size of the inner cavity to only few nanometers, thus limiting the dimension of the internalized molecule as well as its diffusion stream (see Supplementary Note 1). Monolayers structures would instead permit the constructions of tubular shapes with larger inner volumes and thus allow for the encapsulation of larger proteins with a higher chance of binding.

To achieve large cavities, individual planar sheets are normally connected at their edges by flexible hinges. These are short segments of thymine spacers, which allow the faces to self-orient according to the most favorable angle (Supplementary Fig. 10a). Although widely used for construction of tubular prism-like structures, this strategy presents a major drawback: a complete lack of control over the “angular orientation” between connected faces. In other words, considering that each DNA origami face has two distinct sides, a front and a back, the use of flexible hinges does not allow to distinguish between faces connected at an angle α or (360°-α), thus leading to an equal probability of hollow structures with all front (or back) sides pointing inwards or outwards.

With the aim to create DNA hosts whose inner cavity is functionalized for encapsulation purposes, full control of face orientation is necessary. To achieve this goal, we therefore designed two additional sets of out-of-plane crossovers. The first set links the edges of neighboring origami domains from base 0 of one helix to base 14 of the adjacent helix (Supplementary Fig. 10b). This results in a relative orientation of 120° between connected faces. When observing the origami plane in the direction perpendicular to the helical axes and such that the 5’- and 3’-temini of the staples point towards the observer, such a design will ensure that extended staples (colored in orange) are eventually enclosed within the cavity of the host.

Finally, the opposite situation in which extended staples are instead located on the outer surface of the host was achieved by positioning the edge crossovers 7 bp away one from the other, such to allow adjacent faces to be connected at 240° (Supplementary Fig. 10c). Contrarily, when using flexible hinges (Supplementary Fig. 10a), the lack of an imposed angle between adjacent faces will instead result in a mixture of the two possible orientations (120° and 240°), with the protruding arms oriented either inside or outside. Successful achievement of the desired structures using the above-mentioned design strategy has been proven by gel electrophoresis and AFM analysis of biotin-modified DNA hosts loaded with streptavidin (Supplementary Figs. 12-19).

The 3p structure was instead designed according to the honeycomb lattice strategy, with interhelical connections relatively oriented at 120° and periodically repeated every 2 helical turns (i.e. every 21 bp). Also in this case, almost the entire scaffold was used (9 bases left) with a six-helix bundle at each edge of the prism, which ensures a fixed 60° angle between two adjacent faces (Supplementary Fig. 8b, 11b, 20 and 21).

Gel electrophoresis analysis of protein loading

Our study focused on the binding of DegP to a DNA origami host "*ad-hoc*" designed, but in general the procedure can be extended to other proteins, once the specific ligand is known. We noticed that the loading efficiency of the protein might be highly dependent from a series of different factors, which we identified by performing a systematic analysis of binding reactions in distinct experimental conditions (Supplementary Figs. 34-43). In few cases, we decided to omit "uninformative" bands related to the excess of unreacted products and to focus only on informative bands concerning unbound and bound proteins and/or origami structures.

In all gels performed, we observed that the lanes containing the labeled DegP protein (for example, lanes 3 to 5 and lane 7 in Supplementary Figs. 34 and 35) display a large band directly on the loading pocket and slightly above it. This band is visible only upon illumination at the excitation wavelength of the fluorophore used for protein labeling (488 nm in Supplementary Fig. 34 and 633 nm in Supplementary Fig. 35), thus indicating that the protein migrates very slowly and slightly against the applied electric field. The diffusion properties of the DegP protein, when embedded in an agarose gel matrix, are beyond the scope of this work and were not investigated in detail. Nevertheless, we calculated the isoelectric point of the DegP protein in its hexameric form and found a value of about 8 (http://protcalc.sourceforge.net/cgi-bin/protcalc). This would result in a slightly positive net charge (1.0) at the conditions used for gel separation (TBEMg buffer pH 8.0), with a

consequently weak migration rate in the direction opposite to the applied field. However, one should note that this pI estimate assumes that all residues have pKa values that are equivalent to the isolated residues. This is normally not valid for a folded protein and may be also affected by the presence of an undefined number of fluorophore molecules on the protein surface, which is indeed our case.

It is worth noting that the binding of the protein to the peptide-modified DNA origami structure did not result in any remarkable gel mobility shift, although their co-migration clearly proved their mutual interaction (cfr. lane 6 with 7 in Supplementary Figs. 34-35). The gel mobility of the DNA-origami protein complex (and in general of every molecular species) is mainly the result of two contributions: its net charge and total mass. Assuming that in the conditions used the net charge of the DegP protein is almost neutral, its full encapsulation within the DNA host will lead to a negligible variation of the negative surface charge density of the DNA origami structure, thus explaining its almost unaffected migration rate. Finally, one should also consider that the increase in the molecular weight of the DNA origami (ca. 5 MDa) due to DegP binding is only about 5% for $DegP_6$ (ca. 250 kDa) and roughly 10% for $DegP_{12}$ (ca. 500 kDa). From our experience, such a difference in total mass may be difficult to reveal by agarose gel electrophoresis and is highly "protein-dependent" (see for example STV-bound DNA origami structures in Supplementary Fig. 12 and anti-Flc antibodies binding to 6p DNA host cages in Supplementary Fig. 39). The molecular weight of one streptavidin molecule is about 53 kDa. A total of six proteins attached to the DNA host should thus lead to an increase in mass of about 320 kDa, whereas a total of 18 streptavidin moieties should result in ca. 1 MDa mass increase. This suggests that, using streptavidin as protein guest instead of DegP, a comparable increase in the total mass of the complex as with DegP indeed results in a slower rate of the migration band (Supplementary Fig. 12). One should note that this is valid both for proteins bound within the cavity of the host and on its external surface. A similar behavior was observed when loading the 6p host with fluorescein-tagged DNA strands complementary to the inner protruding arms and further reacted with antibodies raised against fluorescein molecules (Supplementary Fig. 39).

To better understand the role of the protein surface charge on its binding to the DNA origami host, we performed a series of loading experiments (Supplementary Figs. 34-43), whose results can be summarized as follows: the encapsulation of the DegP protein into the DNA origami host is ligand specific and the loading efficiency increases with the number of peptide ligands. In addition, *suppression of the net positive charge of the protein* (either increasing the pH of the reaction mixture, or by fluorescent labeling of the exposed amino

acid residues or adding lysine-specific molecular tweezers) *enhances the yield of protein encapsulation*. Protein binding occurs already after few hours and is independent of the oligomerization state of the protein, its mutagenic form (among those here analyzed), labeling grade and labeling chemistry. Finally, DegP binding occurs also within other DNA hollow chambers demonstrating the generality of the method proposed.

Gel analysis of single-strand displacement reactions

We performed a series of experiments to test whether protein delivery could be dependent on defined parameters of the system or specific experimental conditions, such as the size of the loaded protein, the number of inner catching arms, the type of fluorophore used for labeling, the addition of lysine-specific molecular tweezers or competitive peptide substrates, as well as the addition of a large excess of DNA actuator strands or high concentrations of sodium chloride. All gels concerning these experiments are reported in Supplementary Figs. 58-62 and all lead to the same conclusion: *independently of the conditions tested here, once the protein is internalized within the DNA origami host, it cannot be fully released by single-strand displacement reactions*.

In general, upon displacement of the internalized ligands, we indeed observed a slight decrease in the intensity of the previously encapsulated protein (cfr. for example lanes 5 and 6 with lane 4 of Figure 6 of the main manuscript), but we never observed its complete disappearance, thus indicating protein "trapping" inside the DNA cage even in absence of specific peptide ligands. We are still currently investigating the reasons for such an unexpected and interesting behavior. The results obtained until now suggest that the electrostatic interactions taking place between the external protein surface and the inner side of the DNA host are strong enough to keep the two components together even in absence of specific interactions. *DNA-protein electrostatic interactions therefore appear to play a double role: from the one side, their partial depletion seems to be necessary to allow ligand-specific protein encapsulation. From the other side, once encapsulation has taken place, the strength of the electrostatic interactions seems to be sufficient for trapping the protein inside the DNA cage, despite full removal of the ligands*. In other words, whereas the ligands are necessary to induce protein encapsulation, once the complex is formed, their removal does not induce protein release. Clearly, further investigations are necessary to elucidate this point, which promises to open new avenues and opportunities in the construction and control of DNA-protein complexes.

First studies on DegP activity and stability – challenges and future perspectives

The ambition and long-term goal of our work is to investigate the effect of DNA caging on the stability and enzymatic activity of the hosted protein. At this purpose, proteins with a short lifetime and complex activity are ideal guest candidates. In fact, being able to prolong the lifetime of unstable proteins and to control their enzymatic activity would be an enormous step forward in the realization of artificial protein machines. The choice of the DegP protein is in this sense very lucky, although challenging. Besides its special structural properties that we have advantageously used to set up our approach, the DegP protein displays a number of functional characteristics, which make it an interesting candidate to test the utility and potentiality of our method.

DegP is a serine protease that displays an autoproteolytic activity. Cellular stress response leads to elevated levels of DegP in the periplasm, and after the abberant/misfolded substrates have been digested and no more substrates are available, DegP digests itself. This process is believed to represent a way for regulating the amount of DegP in the cell. Although efficient from a cellular perspective, the short lifetime of DegP and the current lack of substrates with a high turnover rate make the system extremely challenging to analyze in activity assays.

Thus, the very first step towards our goal is to establish appropriate assay conditions. Only in this way, we can test whether encapsulation of DegP within the origami cages would lead to its stabilization and therefore also to a reduced autoproteolytic activity. We already started to perform several experiments; nevertheless, the following obstacles still need to be solved. First, the yield of DegP „loaded“ origami structures should be improved. Second, the concentration of DegP within the origami host should be exactly determined. Finally, due to the low amount of material available for activity tests, a high affinity substrate in combination with a high sensitivity output signal (e.g. FRET) would be necessary. In this sense, single-molecule FRET assays would be advantageous.

At the moment, the standard substrate for DegP activity assays is the *p*-nitroaniline derivative of the DPMFKLV peptide (DPMFKLV-pNA), which we used to determine the minimum DegP concentration necessary for long-term experiments and sensible signal detection (Supplementary Figs. 65-67).

Supplementary Methods

Materials and chemicals

All unmodified oligonucleotides were purchased from Sigma-Aldrich as desalted products and delivered lyophilized in 96-well plates. Fluorescein (Flc), carboxytetramethylrhodamine (TAMRA), biotin and thiol-modified oligonucleotides were purchased from Sigma Aldrich in HPLC purification grade and used without further treatment. Single-stranded M13mp18 DNA, propagated in E.coli XL1-Blue (Agilent technologies; cat. # 200249) was produced from phage DNA (Affymetrix; cat. # 71706) as previously reported.[5] Ultrafree-DA Amicon centrifugal filter devices (100,000 Da MWCO, cat. # UFC5003BK; 50,000 Da MWCO, cat. # UFC5050BK; 3,000 Da MWCO, cat. # UFC5050BK) were purchased from Millipore. Vivaspin centrifugal filters (100,000 Da MWCO; cat. #VS0142 and 3,000 Da MWCO; cat. # VS0192) were supplied from Sartorius Biolab Products. NAP™-5 and NAP™-10 columns (cat. # 17-0853-02 and 17-0854-02) were obtained from GE Healthcare. Water was purified on a Milli-Q® Integral Water Purification System (cat. # Z00QSVC01) and further filtered on 0.22 μm membrane filters (cellulose acetate, sterile, cat. # 28145-477) supplied by VWR. Acrylamide/bis solution (19:1; 40% w/v) and ammonium persulfate (APS) were acquired from Sigma Aldrich, tetramethylethylendiamine (TEMED) and 1 kbp DNA ladder from Roth and 10bp DNA ladder from GeneON. Agarose was purchased from Biozym (cat. # 84004), SYBR Gold nucleic acid gel staining from Life technologies and ethidium bromide staining solution from Merck (cat. # 1116080030). Gel extraction of the DNA-origami and their protein complexes was performed using quantum Prep Freeze 'N Squeeze DNA gel extraction spin columns supplied by BioRad (cat. # 732-6165). Monoclonal mouse anti-fluorescein IgG (H+L), CF™ 594 antibody (cat. # SAB4600115) and CF™ 640R antibody (cat. # SAB4600169) were obtained from Sigma-Aldrich. Streptavidin and DegP proteins were produced in our laboratories. Buffers used were 1X TEMg (20 mM Tris base, 2 mM EDTA, 12.5 mM $MgCl_2$, pH 7.6), 1X TBEMg (40 mM Tris base, 20 mM boric acid, 2 mM EDTA, 12.5 mM Mg acetate, pH 8.0) and 1X PBS (3.5 mM NaH_2PO_4, 8 mM Na_2HPO_4, 50 mM NaCl, pH 8.6). For the solid phase peptide synthesis the following chemicals were used (all purchased from Sigma-Aldrich): ACN (acetonitrile), DCM (Dichlormethane), DIPEA (*N,N*-Diisopropylethylamine), DMF (*N,N*-Dimethylformamide), EDT (1,2-Ethanedithiol), HBTU (*N,N,N',N'*-Tetramethyl-*O*-(1*H*-benzotriazol-1-yl)uranium hexafluorophosphate), HOBt (1-Hydroxybenzotriazole), Maleic anhydride (2,5-Furandione), Methanol, Piperidine, TFA (Trifluoroacetic acid), TIS (Triisopropylsilane). Fmoc-protected aminoacids were: Fmoc-

Asp(OtBu)-OH, Fmoc-Leu-OH, Fmoc-Lys(Boc)-OH, Fmoc-Met-OH, Fmoc-Phe-OH, Fmoc-Pro-OH and Fmoc-Val-OH.

Mutation, expression and purification of the DegP protein

Mutagenesis and expression of DegP

All plasmids were derivatives of pCS20 expressing wild-type *degP* with a C-terminal His tag. All constructs were verified by DNA sequencing and point mutants were constructed by oligonucleotide-directed mutagenesis according to standard procedures. For the mutagenesis the following primers were used:

C57A; GATGATTCTCCGTTCAGCCAGGAAGGTTCTC
C69A; GAGCTCTCCGTTCGCCCAGGGTGGCCAG
N146C; CAAATCCAGAACCCGAAATGCCTGACCGCAATTAAGATG
N296C; GTAAGCCAGGTTCGTCCTTGTTCCTCCGCTGCAAAAG
A414C; GGCGCGAACCAGCAGTGTGTGAAAAACATCGCTGAACTG
pPS1_ΔSS For(*BglII*); AAAAAACCATGGCTAGACTTCTTCAGCAAGAC
pPS1_ ΔSS Rev (*Nco*I); AAAAAAAGATCTCTGCATTAACAGGTAGTAG
S210A GATCAACCGTGGTAACGCCGGTGGTGCGCTGG

The mutagenesis of C57A, C69A and S210A was performed via single site directed mutagenesis (PfuUltraHF, Agilent). The catalytically inactive mutant (Δss_3 x cys_SA) was generated by the substitution of the serine at position 210 by alanine. To avoid mislabeling, native cysteine residues in the LA-Loop (C57 and C69) were also mutated to alanine. A schematic representation of all mutations inserted in DegP_Δss_3xCys_SA is given in Supplementary Fig. 1. The amino acids N146, N296, A414 were chosen to be mutated to cysteine for protein labeling with maleimide-activated fluorophores. Sequence alignment showed that the selected amino acids are weakly conserved and expressed on the protein surface both in the 6-mer and 24-mer. Furthermore, the distance between their side chains is larger than 10 nm, preventing formation of disulfide bonds. In addition, each point mutation is located in a distinct protein domain (Supplementary Fig. 2). The mutations N146C, N296C, A414C were introduced with the Change-IT multiple mutation site directed mutagenesis Kit (Affymetrix).

Protein expression, cell fractionation and purification

These steps were performed as described before,[6] adding 1 mM TCEP to each buffer.

MA001 cells expressing the Δss_3xCys_SA plasmid were grown in SOB medium supplemented with 200 μg ml^{-1} ampicillin and 100 μM IPTG at 28°C. After 6 h the cells were harvested by centrifugation (10 min, 6000 rcf, 4°C). The pellets were dissolved in buffer A (50 mM NaH_2PO_4, 300 mM NaCl, 1 mM TCEP, pH 8) and lysed via French press (20000 Pa). The cellular debris was centrifuged (53000 rcf, 40 min, 4°C) and the cell pellet discarded. Samples of the supernatant were mixed with SDS-PAGE sample buffer and analyzed using 10% acrylamide SDS-PAGE. Gels were stained with Coomassie blue. Protein purification was carried out under non-denaturing conditions as previously described.[7] After fractionation, the obtained cell extract was applied on a Ni-NTA column, equilibrated with buffer B (50 mM NaH_2PO_4, 300 mM NaCl, 20 mM imidazole, 1 mM TCEP, pH 8) and eluted with a gradient (13 %, 20 %, 33 %, 40 %, 100 %) of buffer C (50 mM NaH_2PO_4, 300 mM NaCl, 150 mM imidazole, 1 mM TCEP, pH 8). Afterwards, protein purification was performed on a Superdex gel filtration column equilibrated with buffer D (150 mM NaCl, 50 mM NaH_2PO_4, 1 mM TCEP, pH 8).

Protein labeling

Purified samples of DegP Δss_3xCys_SA were labeled with DyLight™ 488 Maleimide, Dylight™ 633 Maleimide or Dylight™ 647 Maleimide (ThermoFisher). The DegP-SA (i.e. mutated only at the catalytic triad) was instead modified at the lysine residues with Alexa 488 N-hydroxysuccinimide ester. Excess label was removed with Pierce™ Dye Removal Columns (Thermofisher) according to manufacturers manual. Although the chemical formulas of Dylight™ fluorophores are not available, their spectral properties are similar to the corresponding Alexa-type dyes. For this reason, all fluorophores will be indicated in the manuscript by an "A" followed by the associated excitation wavelength.

Various combinations of unlabeled or fluorescently labeled DegP proteins in different oligomerization states and genetic forms were screened for their binding to the DNA cage, in order to estimate the role of different factors on the encapsulation properties of the guest (Supplementary Figs. 34-43). All protein forms used in this work, are summarized in Supplementary Table 1.

Synthesis of the DNA-peptide conjugate

Solid phase peptide synthesis (SPPS) was carried out on a Wang resin applying standard Fmoc/tBu chemistry. The peptide Asp-Pro-Met-Phe-Lys-Leu-Val (DPMFKLV) was synthesized according to the following procedure (Supplementary Fig. 4).

Wang resin functionalization and SPPS

The resin was suspended in a minimal amount of CH_2Cl_2/DMF (9:1, v/v) and 4.0 equivalent of Fmoc-Val-OH:HOBt (1:1) in DMF were added. Subsequently, DIC and DMAP were added to the reaction mixture in 4 and 0.1 equimolar amounts (eq.), respectively. The resin suspension was shaken for 24 hrs at room temperature in a flask equipped with a drying tube. Unreacted hydroxyl groups were capped with 2 equivalents of acetic anhydride in presence of pyridine for 30 min. The reaction mixture was then transferred into a typical SPPS reactor and the resin was washed 2 times with DCM/DMF. The yield of resin functionalization was determined spectrophotometrically, by measuring the amount of Fmoc released from a known amount of dried resin. The aminoacids were coupled according to standard Fmoc/tBu SPPS procedures: typically, the Fmoc protecting group was removed in 20% piperidine/DMF for 20 min and, upon thorough resin washing, the successive Fmoc-aminoacid (4 eq) was added in presence of HOBt:HBTU:DIPEA (4:4:4). The mixture was then shaken for 30 min at room temperature.

Functionalization of the N-terminus with a maleimide group

The N-terminus of the peptide was functionalized with a maleimide group for further Michael addition with the thiol function at the 5'-terminus of the oligonucleotide. Maleic anhydride was added to the resin mixture (4 eq.) and let react until completion (checked by HPLC). Upon washing, cyclization was achieved by adding HOBt:HBTU:DIPEA (4:4:4). The resin was washed again 2 times with DCM/DMF, the peptide was cleaved from the resin in presence of TFA:H_2O:EDT:TIS (v/v 92.5:2.5:2.5:2.5), for 2 hrs at room temperature and the solvent was removed under vacuum. The resulting solid product was dissolved in a mixture of ACN:H_2O (1:1, v/v) and purified by HPLC, using a gradient of acetonitrile (ACN) /water (ACN + 0.1% TFA (A); H_2O + 0.1% TFA (B)) from 25% B to 35% B in 60 minutes. After removing the solvent, the product was lyophilized and immediately used for covalent binding to the thiol-modified oligonucleotide.

Peptide coupling to the oligonucleotide

The 5' thiol-modified oligonucleotide (named as A1: GTGGAAAGTGGCAATC) was dissolved in PBS 1X, treated with 50 equivalents of TCEP and further purified by gel filtration using NAP™-5 and NAP™-10 columns. The solution was then concentrated to ca. 20 μL using a 3000 Da MWCO ultra centrifugal filter unit. The lyophilized peptide was then dissolved in dd H_2O:ACN (1:10, v/v) to a concentration of 126 mM and mixed with 0.02 equimolar amounts of the thiol-modified oligonucleotide. 100 μL of dd H_2O:ACN (1:1, v/v) were added to a final concentration of 29 mM. The reaction mixture was let running for 48 hrs at 30 °C, then concentrated to a final volume of about 20 μL using a 3000 Da MWCO ultra centrifugal device and the target product was purified by denaturing PAGE (25% acrylamide in TBE 1X buffer at 220 V for 45 minutes at room temperature).

The purified DNA-peptide conjugate was finally characterized by denaturing gel-electrophoresis, using a Typhoon FLA 9000 from GE healthcare Life Sciences (Supplementary Fig. 5) and by MALDI spectrometry (Supplementary Fig. 6). We finally obtained the following conjugates: A1-DPMFKLV (120 μM), Flc-A1-DPMFKLV (78 μM) and TAMRA-A1-DPMFKLV (174 μM).

Assembly of the DNA origami host

Unless stated differently, the 6prism origami structures were assembled using a 1:10 molar ratio between the M13mp18 viral DNA (20 nM) and each of the staple strands, in 1X TEMg buffer (20 mM Tris, 2 mM EDTA, 12.5 mM $MgCl_2$, pH 7.6). Thermal annealing was performed by decreasing the temperature from 90 °C to 20°C at -1°C min^{-1} on a Thermocycler Mastercycler nexus gradient (Eppendorf). For the 3prism chambers, a screening of different magnesium buffer concentrations and annealing temperatures was performed in order to find the optimal conditions for folding (Supplementary Fig. 21). Best yields were obtained at 12 mM magnesium concentration and a thermal annealing from 90°C to 45°C (-1°C min^{-1}) followed by a slow cooling from 44°C to 20°C (-1°C every 150 min).

Both kinds of DNA origami hosts were decorated in their inner cavity with a distinct number of DNA strands of identical sequence (named as cA1: CTTCACGATTGCCACTTTCCAC), which were partially complementary to the A1-tagged peptide (full nomenclature of the DNA hosts used in this work is given in Supplementary Table 2). Besides the A1-peptide conjugate for specific encapsulation of the DegP protein, biotin- and fluorescein-modified A1 sequences were also used to catch, respectively, streptavidin molecules (STV) or monoclonal antibodies

raised against fluorescein (anti-Flc^{CF594} and anti-Flc^{CF640R}). A full list of the linkers and corresponding targets used in this work is given in Supplementary Table 5. If not stated differently, A1-tagged molecules were hybridized inside the cavity of the channels in a one-pot reaction mixture, using a molar ratio of M13 scaffold : staples : A1-conjugate = 1 : 10 : x (with x = 60, 150 or 180, respectively for the 6cA1, 15cA1 or 18cA1 constructs). The so-functionalized DNA origami structures were then purified and finally reacted with the corresponding proteins as described below.

Loading the DNA origami host with DegP or other proteins

Loading of DegP protein

After thermal annealing, the excess of staples and unreacted DNA-peptide conjugates were removed from the peptide-modified origami solution by thorough washing. At this purpose, typically 200 μl of the assembled origami solution were concentrated to about 10 μl in an Amicon centrifugal filter device (MWCO 100,000 Da) by 3 min centrifugation at 4500 rcf and 20 °C. The filtrate was then washed in 300 μl TEMg 1X and concentrated to minimal volume. This step was done twice. Alternatively, the desired band was extracted from the gel and the product was recovered by Freeze 'N Squeeze™ spin columns. Finally, the so-purified peptide-modified origami structure was reacted with 25 molar equivalents of DegP protein for at least 3-4 hrs (till max 12 hrs) at room temperature under constant shaking by 200 rpm. The reaction mixture was then analyzed by agarose gel electrophoresis using 0.75% agarose in 1X TBEMg buffer and running the gel at 80 V for ca. 2 hrs at 4°C. The gel was scanned at a Typhoon FLA 9000 using selected excitation wavelengths and optical filters to record the presence of the DegP protein (labeled with A488, A633 or A647) and the DNA-peptide conjugate (labeled at its 3'-terminus either with a TAMRA or a Flc molecule). The desired band was excised with a clean scalpel from an identical gel lacking ethidium bromide (to avoid structure deformation due to dye intercalation) and loaded into a Freeze 'N Squeeze™ spin column. The target compound was recovered after centrifugation at 7500 rcf for 2 min at 4 °C and directly used for AFM, TEM characterization or for further experiments. A detailed description of the effect of different factors on the loading efficiency of DegP protein is given in the Supplementary Discussion. These factors include the reaction time, the molar excess of DegP, its oligomerization state, mutagenic form, pH of the reaction mixture, type of fluorescent dyes used for protein labeling as well as the presence or absence of lysine-specific molecular tweezers.

Loading of other protein cargos

Besides DegP, additional proteins have been used as cargos and loaded inside the 6p DNA hosts: namely, streptavidin (STV), CF594 and CF640R monoclonal mouse anti-fluorescein antibodies (anti-Flc^{CF594} and anti-Flc^{CF640R}).

After purification by ultracentrifugation, the biotin-modified origami were directly reacted with 1.5 equimolar amounts of streptavidin, let react for 2 hrs at room temperature and immediately analyzed either by agarose gel electrophoresis (Supplementary Fig. 12) or by atomic force microscopy (Supplementary Figs. 13-19). Fluorescein-tagged origami solutions were instead reacted overnight with 15-fold excess of anti-Flc^{CF594} or anti-Flc^{CF640R} at room temperature under constant shaking at 200 rpm. The complexes were then analyzed by agarose gel electrophoresis (Supplementary Fig. 39).

Calculation of the yield by gel electrophoresis

The yield of protein binding has been calculated using ImageJ, marking the bands of interest with an identical rectangular shape and measuring the area under the intensity curve. Relative intensities corresponding to the products analyzed under the same illumination conditions have been compared. For errors inferior to 10%, we considered reasonable to quantify the signals visible under different wavelengths and calculated the relative yield of protein loading as P/D = intensity protein band / intensity DNA band. Similarly, the yield of DNA labeling has been calculated as L/D = intensity labeled-peptide band / intensity DNA band (see example in Supplementary Fig. 43).

Molecular dynamics simulations and atomistic models

Computational details

Initial coordinates for the 24-mer, 12-mer and hexamer forms of DegP ($DegP_{24}$, $DegP_{12}$ and $DegP_6$ respectively) were taken from the Protein Data Bank (PDB codes 3CS0, 3OTP and 1KY9, respectively).[8-10] The DNA strands were generated from the sequence as linear segments with a B helix type. Coordinates for the peptide were minimized after being generated from the sequence in a β-strand conformation since binding of the peptide to the PDZ1 domain of DegP takes place by β-sheet extension. The origami cage was modeled using only the protruding DNA helices. The distances and relative position of the 5’ residues in the protruding DNA strands were restrained to simulate the DNA strands distribution in the origami. For this purpose the colvar module of NAMD was used.[11]

Since $DegP_{24}$ can simultaneously host more than one DNA helix per binding site in the central cavity, the binding of several (4 or 5) DNA helices to $DegP_{24}$ was investigated using Molecular Dynamics simulations (Supplementary Fig. 22). The missing parameters for the MD simulations were generated using Swissparam.[12] Missing loops were built with Modeller9.10.[13] All simulations were performed using NAMD2.9[14] with the temperature set to 300 K and a time step of 2 fs for simulation times of up to 10 ns. The CHARMM36 force field, which has shown to reproduce well the properties of DNA origami structures, was used together with the TIP3P water model.[15,16] The simulations were carried out in the NPT ensemble.[17] The resulting systems contained about 1.5 millions atoms.

The multimeric forms of DegP

$DegP_{24}$, $DegP_{12}$ and $DegP_6$ have been characterized using X-Ray crystallography (Fig. 1a of the main manuscript).[8-10] In all three states the monomers are arranged around a central cavity of increasing size. This arrangement in $DegP_6$ places the PDZ1 domains in positions pointing toward the solvent. The PDZ1 domains in $DegP_{12}$ and $DegP_{24}$ are located at the entrances of the central cavity. In $DegP_{12}$, the PDZ1 domains are grouped in units of three arranged in a tetrahedral orientation. The resulting space between the PDZ1 domains only allows the entrance of one DNA helix. Conversely, $DegP_{24}$ features a cube with six possible entrances to the central cavity, formed each by four PDZ1 domains. The entrances to the central cavity are big enough to host up to three DNA helices simultaneously without compromising the binding of the incoming peptide to the PDZ1 domain. Our MD simulations show that, after 10 ns there is a proper binding of the peptide to the PDZ1 domain independently of the number of DNA helices simultaneously present in the cavity (Supplementary Table 3).

Atomistic models for the 6p construct

The structures represented in Supplementary Figs. 24-33 are geometrical models representing how DegP might be found inside the 6prism origami. Three models for the origami host were considered, a regular hexagon (h), a distorted hexagon (hr) and a rectangle (r). The origami is depicted by the vertices of the geometric figures describing it and the points where the protruding DNA strands are located (Supplementary Fig. 23). The first point is placed always in the lower left corner and points are numbered counterclockwise. The code for each structure is as follow:

1p1b4l-24hr1

1p: only 1 protein in the model

1b: only 1 DNA helix binding to the origami and the protein

4l: this field applies to multiple ligands and it specifies which origami faces are involved in the binding. A value of 4l means that face 1 and 4 are involved in ligand binding. A value of 2l means protein binding to adjacent faces of the origami. When there is only one binding site and one protein, this field assumes the default value of 0l

24: the oligomerization state of DegP in the model

hr: the geometric model applied to the origami structure: i.e. a deformed hexagon in this case

1: in case more binding modes are possible, these are numbered as 1 or 2 .

Atomic force microscopy imaging and data analysis

The sample was deposited on freshly cleaved mica surface (Plano GmbH) and adsorbed for 3 min at room temperature. After washing with ddH_2O, the sample was dried under gentle argon flow and scanned in ScanAsyst Mode using a MultiMode™ microscope (Bruker) equipped with a Nanoscope V controller. 0.4 N/m force constant cantilevers with sharpened pyramidal tips (ScanAsyst-Air tips, Bruker) were used for scanning. After engagement the peak force setpoint was typically 0.02 volt and the scan rates about 1 Hz. Several AFM images were acquired from different locations of the mica surface to ensure reproducibility of the results. All images were analyzed using the Gwyddion software.

Statistical analysis of AFM images

At least three independent preparations of each sample were analyzed by AFM and several images were acquired from different regions of the mica surface (typically 3 μm x 3 μm). The results obtained were analyzed as follows: for the unmodified DNA-origami, the yield of origami formation ($P_{origami}$) was calculated as the percentage of well-formed structures ($N_{well\text{-}formed}$) over the total number of structures observed (N_{tot}).

$P_{origami} = (N_{well\text{-}formed}/ N_{tot}) \times 100$

The yield of protein binding to the DNA origami structures ($P_{origami\text{-}protein\ complex}$) was instead calculated as the percentage of loaded structures (detectable from the increased height profile) over the amount of well-formed structures:

$P_{origami\text{-}protein\ complex} = (N_{loaded\ structures}/ N_{well\text{-}formed}) \times 100$

All results obtained are summarized in Supplementary Table 6.

Negative stain electron microscopy (EM) and data analysis

Grid preparation and image recording

Protein and protein-DNA origami samples, were prepared for EM as previously described.[18,19] Briefly, 4 μl sample droplets were applied on glow discharged copper grids (Agar Scientific; G2400C), covered by a 8 nm thick continuous carbon film. After 60 sec, excess of buffer was blotted from the side with a piece of filter paper (Whatman no. 4). In case of DNA origami samples, this procedure was repeated in order to increase the concentration of the particles on the grid. The grid was washed twice with two drops of ddH_2O and subsequently stained with two drops 1% Uranyl acetate. All images were taken with a FEI Tecnai G2 Spirit electron microscope equipped with a Lab_6 cathode at an operation voltage of 120 kV. Digital micrographs were recorded with a 4k x 4k CMOS Camera F416 (TVIPS) using low-dose conditions.

EM of negatively stained $DegP_6$ and $DegP_{12/24}$

Digital micrographs of $DegP_6$ and $DegP_{12/24}$ were recorded at a nominal magnification of 30.000x and 67.000x, resulting in a pixel size of 0.358 and 0.166 nm, respectively. For $DegP_6$, we analyzed approximately 10000 particles from 121 images, for $DegP_{12/24}$ 9804 particles from 28 images. Single particles were boxed out manually using e2boxer[20] and aligned and classified using reference-free alignment and k-means clustering procedures as implemented in the SPARX software package.[21] Class-averages of the $DegP_{12/24}$ dataset were visually inspected and separated into two subsets, showing typical views of $DegP_{12}$ and $DegP_{24}$, respectively. A second round of classification was then performed within each subset using the ISAC approach of the SPARX software package.[22] The final class-averages of $DegP_6$, $DegP_{12}$ and $DegP_{24}$ include 50-100 members. Selected class averages were then clipped to a box-size of 256 pixels, scaled to a final pixel size of 0.358 nm, filtered to a nominal resolution of 0.2 nm and masked with a soft Gaussian round mask, in order to allow a better direct comparison with the class averages of the origami complexes.

EM of negatively stained $DegP_{12/24}$ loaded $6p^{120}$-18cA1 host

Digital micrographs of $DegP_{12/24}$ filled cages were recorded at a nominal magnification of 30.000x, resulting in a pixel size of 0.358 nm. For $DegP_{12/24}$ loaded cages we analyzed approximately 18084 particles from 1304 images. Single particles were boxed out manually using e2boxer and aligned and classified using a user defined rectangular mask, excluding the interior of the cage. Classification was performed by k-means clustering procedures as implemented in the SPARX software package. Class-averages of $DegP_{12/24}$ filled cages were

visually inspected and separated into two subsets, showing filled and unfilled cages, respectively. A second round of classification was then performed within each subset using the ISAC approach of the SPARX software package. Class-averages of filled cages obtained after the second round were again visually inspected and separated into two subsets, showing $DegP_{12}$ and $DegP_{24}$ filled cages, respectively. Final class-averages of the empty cages, $DegP_{12}$ and $DegP_{24}$ filled cages include 15-50 members.

EM of negatively stained $DegP_6$ loaded $6p^{120}$-18cA1 host

Digital micrographs of $DegP_6$ loaded cages were recorded at a nominal magnification of 30.000x, resulting in a pixel size of 0.358nm. For the $DegP_6$ loaded cages, 32976 particles from 645 images were analyzed. Single particles were boxed out manually using e2boxer and aligned and classified using a user-defined mask, as described above. Clustering was performed by k-means clustering procedures as implemented in the SPARX software package. Class-averages of protein filled cages were visually inspected and separated into three subsets, showing $DegP_6$, and few exemplars of $DegP_{12}$ and $DegP_{24}$ filled cages, respectively. A second round of classification was then performed within each subset using ISAC. Final class-averages of $DegP_6$, $DegP_{12}$ and $DegP_{24}$ filled cage include 15-50 members.

Supplementary References

1. Andersen, E.S., *et al.* Self-assembly of a nanoscale DNA box with a controllable lid. *Nature* **459**, 73-76 (2009).
2. Douglas, S.M., Bachelet, I. & Church, G.M. A logic-gated nanorobot for targeted transport of molecular payloads. *Science* **335**, 831-834 (2012).
3. Zhao, Z., Jacovetty, E.L., Liu, Y. & Yan, H. Encapsulation of gold nanoparticles in a DNA origami cage. *Angew. Chem. Int. Ed.* **50**, 2041-2044 (2011).
4. Endo, M., Hidaka, K., Kato, T., Namba, K. & Sugiyama, H. DNA prism structures constructed by folding of multiple rectangular arms. *J. Am. Chem. Soc.* **131**, 15570-15571 (2009).
5. Castro, C.E., *et al.* A primer to scaffolded DNA origami. *Nat. Methods* **8**, 221-229 (2011).
6. Merdanovic, M., *et al.* Determinants of structural and functional plasticity of a widely conserved protease chaperone complex. *Nat. Struct. Mol. Biol.* **17**, 837-843 (2010).
7. Spiess, C., Beil, A. & Ehrmann, M. A temperature-dependent switch from chaperone to protease in a widely conserved heat shock protein. *Cell* **97**, 339-347 (1999).
8. Kim, S., Grant, R.A. & Sauer, R.T. Covalent linkage of distinct substrate degrons controls assembly and disassembly of DegP proteolytic cages. *Cell* **145**, 67-78 (2011).
9. Krojer, T., Garrido-Franco, M., Huber, R., Ehrmann, M. & Clausen, T. Crystal structure of DegP (HtrA) reveals a new protease-chaperone machine. *Nature* **416**, 455-459 (2002).
10. Krojer, T., *et al.* Interplay of PDZ and protease domain of DegP ensures efficient elimination of misfolded proteins. *P. Natl. Acad. Sci. USA* **105**, 7702-7707 (2008).
11. Henin, J., Fiorin, G., Chipot, C. & Klein, M.L. Exploring Multidimensional Free Energy Landscapes Using Time-Dependent Biases on Collective Variables. *J. Chem. Theory Comput.* **6**, 35-47 (2010).
12. Zoete, V., Cuendet, M.A., Grosdidier, A. & Michielin, O. SwissParam: a fast force field generation tool for small organic molecules. *J. Comput. Chem.* **32**, 2359-2368 (2011).
13. Eswar, N., *et al.* Comparative protein structure modeling using Modeller. *Curr. Protoc. Bioinformatics* **Chapter 5**, Unit 5 6 (2006).
14. Phillips, J.C., *et al.* Scalable molecular dynamics with NAMD. *J. Comput. Chem.* **26**, 1781-1802 (2005).
15. Vanommeslaeghe, K. & MacKerell, A.D., Jr. CHARMM additive and polarizable force fields for biophysics and computer-aided drug design. *Biochim. Biophys. Acta* **1850**, 861-871 (2015).
16. Jorgensen, W.L., Chandrasekhar, J., Madura, J.D., Impey, R.W. & Klein, M.L. Comparison of simple potential functions for simulating liquid water. *J. Chem. Phys.* **79**, 926-935 (1983).
17. Yoo, J. & Aksimentiev, A. In situ structure and dynamics of DNA origami determined through molecular dynamics simulations. *P. Natl. Acad. Sci. USA* **110**, 20099-20104 (2013).
18. Erkelenz, M., *et al.* A facile method for preparation of tailored scaffolds for DNA-origami. *Small* **10**, 73-77 (2014).

19. Gatsogiannis, C., *et al.* A syringe-like injection mechanism in Photorhabdus luminescens toxins. *Nature* **495**, 520-523 (2013).
20. Tang, G., *et al.* EMAN2: an extensible image processing suite for electron microscopy. *J. Struct. Biol.* **157**, 38-46 (2007).
21. Hohn, M., *et al.* SPARX, a new environment for Cryo-EM image processing. *J. Struct. Biol.* **157**, 47-55 (2007).
22. Yang, Z., Fang, J., Chittuluru, J., Asturias, F.J. & Penczek, P.A. Iterative stable alignment and clustering of 2D transmission electron microscope images. *Structure* **20**, 237-247 (2012).
